# Microvascular and Macrovascular Complications and Their Associated Risk Factors Among Patients with Type 2 Diabetes in Saudi Arabia: A Systematic Review and Meta-Analysis

**DOI:** 10.3390/diagnostics16203433

**Published:** 2026-10-21

**Authors:** Mohammed Z. Alqahtani, Amirah A. Alzaki, Ghala M. Asiri, Mada H. Al Jibril, Bayan M. Alhashidi, Khalid W. Binhamran, Najmah A. Alotaibi, Saad A. Alqahtani, Taif M. Alqahtani, Maymunah M. Alassiri, Assem Gebreal, Sarah A. Al Khalid, Ghada Mahmoud Ismail, Eman A. Alqahtani, Ramy Mohamed Ghazy

**Affiliations:** 1 College of Medicine, King Khalid University, Abha 21589, Saudi Arabia; galaasiri989@gmail.com (G.M.A.); madajbril@gmail.com (M.H.A.J.); saad98wep@gmail.com (S.A.A.); dr.taifii90j@gmail.com (T.M.A.); alassirimaymunah@gmail.com (M.M.A.); saralk2003@gmail.com (S.A.A.K.); 2 College of Medicine, University College Dublin, D04 V1W8 Dublin, Ireland; 3 College of Medicine, King Abdulaziz University, Jeddah 21589, Saudi Arabia; bayanmohammad098@gmail.com (B.M.A.); binhamrankhalid@gmail.com (K.W.B.); 4 Al-Hada Armed Forces Hospital, Taif 21944, Saudi Arabia; najmah-ali@outlook.sa; 5 College of Medicine, Alexandria University, Alexandria 21561, Egypt; assem.ahmed1801@alexmed.edu.eg; 6 Community, Environmental and Occupational Medicine Department, Faculty of Medicine, Ain–Shams University, Cairo 11591, Egypt; ghadaismail@med.asu.edu.eg; 7 Internal Medicine Department, College of Medicine, King Khalid University, Abha 11591, Saudi Arabia; aaamerai@kku.edu.sa; 8 Tropical Health Department, High Institute of Public Health, Alexandria University, Alexandria 21526, Egypt; ramy_ghazy@alexu.edu.eg; 9 Family and Community Medicine Department, College of Medicine, King Khalid University, Abha 21589, Saudi Arabia

**Keywords:** type 2 diabetes mellitus, microvascular complications, macrovascular complications, Saudi Arabia, risk factors, diabetic nephropathy, diabetic retinopathy, diabetic neuropathy

## Abstract

**Background/Objectives**: Type 2 diabetes mellitus (T2DM) is a major public health challenge in Saudi Arabia and is associated with substantial morbidity through vascular complications. This systematic review and meta-analysis estimated the pooled prevalence of diabetes-related complications and examined study-level risk factors among Saudi adults with T2DM. **Methods**: The review followed PRISMA guidance and was registered in PROSPERO (registration number: CRD420251032235). Random-effects models were used, and heterogeneity, risk of bias, small-study effects, subgroup effects, influence, and study-level moderators were assessed. **Results**: Of 743 records identified, 36 unique, non-overlapping datasets contributed to at least one quantitative synthesis. Study quality was good in 25 studies (69.4%). Pooled prevalences were 24% for retinopathy (95% CI 18–30%; 26 studies), 16% for nephropathy (11–23%; 22 studies), 24% for neuropathy (16–34%; 27 studies), 3% for cerebrovascular stroke (2–6%; 10 studies), 10% for myocardial infarction (MI) and broader coronary heart disease/coronary artery disease (CHD/CAD) (6–15%; 16 studies), 5% for peripheral arterial disease (PAD; 2–11%; 10 studies), and 10% for diabetic foot (4–26%; nine studies). Heterogeneity was extreme (I^2^ = 93.5–99.3%), and prediction intervals were wide. Excluding influential outliers left pooled estimates essentially similar but reduced τ^2^. In meta-regression, no moderator was significantly associated with retinopathy, stroke, or diabetic foot prevalence (all *p* > 0.05). For nephropathy, longer diabetes duration (β = −0.254, 95% CI −0.411 to −0.097, *p* = 0.005; 52.5% of between-study variance) and female proportion (β = 0.036, 95% CI 0.005 to 0.067, *p* = 0.026; 18.8%) were significant. For neuropathy, smoking prevalence was significant (β = 0.093, 95% CI 0.001 to 0.185, *p* = 0.047; 18.8%). For PAD, female proportion was inversely associated with prevalence (β = −0.084, 95% CI −0.147 to −0.021, *p* = 0.015; 53.2%). Region explained 84.5% of the heterogeneity in MI/CHD/CAD, with lower estimates in Tabuk and Taif, and HbA1c explained 24.5% of the heterogeneity in stroke (*p* = 0.167). Year of publication explained <10.0% of the heterogeneity in every parsimonious model. Substantial residual heterogeneity remained in all models (residual I^2^ 83.0–99.9%). Individual studies also frequently reported associations of complications with poor glycemic control, longer diabetes duration, hypertension, dyslipidemia, obesity, smoking, and physical inactivity. **Conclusions**: Diabetes-related complications impose a substantial burden in Saudi adults with T2DM, but estimates vary markedly across populations, clinical settings, and diagnostic definitions. The findings support standardized screening and improved primary research using consistent case definitions; study-level meta-regression findings should be interpreted cautiously.

## 1. Introduction

Diabetes mellitus (DM), particularly type 2 diabetes mellitus (T2DM), represents a major global public health challenge [1]. In 2021, an estimated 529 million people worldwide were living with DM, corresponding to an age-standardized prevalence of 6.1%. The highest prevalence rates were reported in North Africa and the Middle East. T2DM accounted for most diabetes cases and disability-adjusted life years (DALYs), with a particularly substantial burden observed among older adults [1,2,3]. According to projections from the International Diabetes Federation (IDF), the global prevalence of diabetes is expected to continue increasing, with more than 1.3 billion people projected to be living with diabetes by 2050 and the age-standardized prevalence expected to exceed 10% [3].

DM is a major public health challenge in Saudi Arabia, which ranks among the five countries with the highest diabetes burden worldwide [3]. The age-standardized prevalence of DM is 23.1% [4], with a pooled national prevalence of 24.1% for prediabetes [5]. Global Burden of Disease (GBD) data from 1990 to 2023 show a 117.3% increase in age-standardized diabetes prevalence, alongside increases in incidence (97.2%), DALYs (112.9%), and mortality (132.5%), with mortality rising from 25.26 to 58.74 per 100,000 population. T2DM accounted for most of this burden, with prevalence increasing by 118.4%. Additionally, sex stratification showed that males consistently had higher rates than females across multiple measures, with the sex disparity widening over time [4].

T2DM is strongly associated with a broad spectrum of vascular complications, which are conventionally classified as microvascular or macrovascular. These complications, including retinopathy, nephropathy, neuropathy, coronary artery disease (CAD), and stroke, contribute substantially to the morbidity and mortality associated with T2DM [6,7]. Importantly, the prevalence, clinical presentation, and risk of these complications may differ between men and women, reflecting differences in biological, hormonal, metabolic, and behavioral factors [8].

Existing studies have reported wide differences in the prevalence of microvascular and macrovascular complications, potentially reflecting differences in geographic location, healthcare settings, study populations, diabetes duration, diagnostic methods, and definitions of complications [9,10]. This variation was particularly evident for microvascular complications, especially diabetic retinopathy, for which prevalence estimates across different regions of Saudi Arabia range from 6.9% to 78.0% [11,12]. Macrovascular complications are also prevalent among people with DM in Saudi Arabia, with reported prevalence of CAD, diabetic foot, and stroke at 3.7%, 13.1%, and 17.0%, respectively [13].

The development and progression of these DM-related complications are associated with longer disease duration, particularly poor glycemic control and longer disease duration, which can further impair patients’ quality of life [13,14,15]. Although age and longer disease duration are non-modifiable risk factors for these complications, their risk can be reduced through optimal management of modifiable risk factors, including glycemic control, blood pressure, body weight, and lipid levels, particularly in older adults and those with longer diabetes duration [16]. Considering these modifiable conditions, the growing burden of diabetes and its related complications highlights an urgent need for robust national data to shape health policy, direct resource allocation, and enable the implementation of effective prevention and management strategies [6]. Understanding the contribution of these factors among the Saudi population is important for identifying patients at higher risk and informing targeted prevention and screening strategies. The substantial burden of these complications underscores the need for targeted interventions, including community-based initiatives and structured screening programs, to reduce their impact on patients’ health-related quality of life [17,18].

Therefore, systematic synthesis of the available evidence is needed to provide more precise estimates of the prevalence of diabetes-related complications and to examine sources of variation between studies. Such an analysis can also help identify consistently reported risk factors and highlight areas where evidence remains limited. This review aimed to estimate the pooled prevalence of microvascular and macrovascular complications among patients with T2DM in Saudi Arabia based on evidence from observational studies. It also sought to assess regional variations in the prevalence of these complications and to identify and synthesize their associated risk factors in the Saudi population. The findings are intended to inform evidence-based health policy, support targeted screening and prevention strategies, and contribute to efforts to reduce the burden of diabetes-related complications in Saudi Arabia.

## 2. Materials and Methods

### 2.1. Protocol and Reporting Guidelines

This systematic review and meta-analysis was conducted following the PRISMA (Preferred Reporting Items for Systematic Reviews and Meta-Analyses) guidelines, and the study protocol was prospectively registered on PROSPERO (registration ID: CRD420251032235).

### 2.2. Study Outcomes

The primary study outcome was to estimate the pooled prevalence of microvascular complications, including diabetic retinopathy, nephropathy, neuropathy, and macrovascular complications, including cardiovascular disease, cerebrovascular disease, and peripheral arterial disease (PAD), as well as diabetic foot disease. The study also aimed to examine factors associated with these complications, including hypertension, glycemic control (HbA1c), and dyslipidemia.

### 2.3. Operational Definitions

- Microvascular complications refer to diabetes-related complications involving small blood vessels and microvascular structures, including diabetic retinopathy, diabetic kidney disease (nephropathy), and diabetic neuropathy.
- Macrovascular complications refer to diabetes-related atherosclerotic disease affecting large and medium-sized arteries, including CAD, cerebrovascular disease, and PAD. These conditions may result in myocardial infarction (MI) and broader coronary heart disease/coronary artery disease (CHD/CAD), stroke, and lower-extremity ischemia.
- Diabetic foot disease refers to a distinct and multifactorial diabetes-related complication of the foot that arises from the interplay of PAD (a macrovascular complication), diabetic peripheral neuropathy (a microvascular complication), and other contributing factors such as infection, biomechanical abnormalities, and impaired wound healing. Accordingly, diabetic foot disease was not classified exclusively as either a microvascular or macrovascular complication but was analyzed as a separate composite outcome reflecting the combined burden of both vascular pathways.

### 2.4. Search Strategy

Search terms were developed and refined in consultation with a research librarian to maximize the retrieval of potentially eligible studies. A comprehensive literature search was conducted using three bibliographic databases: PubMed, Web of Science, and Ovid, supplemented by a search of Google Scholar to identify additional potentially eligible studies. The search was initiated on 1 April 2025 and completed on 5 April 2025. The search strategy combined terms related to T2DM, vascular complications, prevalence, and Saudi Arabia. The search strategy incorporated the following terms: (type 2 diabetes OR T2DM OR diabetes mellitus) AND (vascular complications OR microvascular complications OR macrovascular complications OR retinopathy OR nephropathy OR neuropathy OR cardiovascular disease OR peripheral artery disease OR stroke) AND (prevalence OR epidemiology OR frequency OR burden) AND (“Saudi Arabian population” OR KSA).

Studies were assessed for eligibility according to predefined inclusion and exclusion criteria based on the Condition, Context, and Population (CoCoPop) framework. The condition of interest was diabetes-related complications, including microvascular complications, macrovascular complications, and diabetic foot disease. The context included healthcare and community settings in which these complications were assessed among individuals with diabetes. The population comprised individuals with diabetes who were included in the eligible studies. Backward citation searching was performed during full-text screening to identify additional relevant studies, while forward citation searching was conducted using PubMed and Google Scholar. All retrieved records were imported into a single EndNote 20 library (Clarivate, Philadelphia, PA, USA), and duplicate records were systematically identified and removed before screening.

### 2.5. Eligibility Criteria

Inclusion criteria were (1) studies involving adults (≥18 years) with T2DM who were re-siding in Saudi Arabia; (2) observational studies reporting the prevalence and/or risk factors of one or more microvascular, macro-vascular complications or diabetic foot and evaluating factors associated with vascular complications, including hypertension, glycemia control (HbA1c), and dyslipidemia, with or without a comparison between participants with and without complications; (3) full-text articles published in English; and (4) studies published on or before April 2025.

Exclusion criteria were (1) studies involving non-Saudi populations or mixed populations without separately reported data for Saudi participants; (2) interventional studies, including clinical trials and drug efficacy studies; and (3) reviews, editorials, commentaries, letters, conference abstracts, animal studies, case reports, and case series.

### 2.6. Study Selection Process

Two independent reviewers screened studies in two stages: (1) title and abstract screening, followed by (2) full-text review. Title and abstract screening were conducted using Rayyan (Rayyan Systems Inc., Cambridge, MA, USA; https://www.rayyan.ai/), an online systematic review management platform. When full texts were unavailable, corresponding authors were contacted via email to request the full manuscripts. Study eligibility was assessed independently by two reviewers according to predefined inclusion and exclusion criteria. Inter-reviewer agreement was substantial for title and abstract screening (k = 0.82) and full-text screening (k = 0.88). Discrepancies were resolved through discussion, with arbitration by two expert reviewers when consensus could not be reached.

### 2.7. Data Extraction

All eligible studies were entered into a standardized data extraction form developed in Microsoft Excel. The following information was extracted:

(1) Study characteristics: Last name of the first author and year of publication, study identification number, journal name, region of Saudi Arabia, study design, study setting, study population, and sample size (Supplementary File S1).
(2) Participant and clinical characteristics: Mean age and standard deviation (SD), age range where available, sex distribution, mean duration of diabetes, and relevant comorbidities.
(3) Diabetes and glycemic characteristics: Diagnostic criteria for diabetes and diabetes-related complications, mean HbA1c ± SD or reported range, and glycemic control status. Where reported, participants were categorized as having controlled glycemia (HbA1c ≤ 7%), uncontrolled glycemia (HbA1c > 7–9%), or highly uncontrolled glycemia (HbA1c > 9%).
(4) Risk factors and outcomes: Assessed risk factors, including hypertension, dyslipidemia, body mass index (BMI), and other reported clinical or demographic factors; prevalence estimates and measures of association, including odds ratios (ORs) with corresponding 95% confidence intervals (CIs), where available; and outcomes of interest, including microvascular complications (retinopathy, nephropathy, and neuropathy), macrovascular complications (cardiovascular disease, cerebrovascular disease, PAD), and diabetic foot disease. Data extraction was conducted independently by two reviewers using a predefined data extraction form. All extracted data were cross-checked to minimize errors and duplication. Discrepancies between reviewers were resolved through discussion, with consultation of a third reviewer (RMG) when necessary. See Supplementary File S1.

### 2.8. Risk-of-Bias Assessment

The methodological quality of the included studies was independently assessed by two reviewers using the appropriate Joanna Briggs Institute (JBI) critical appraisal checklist according to study design. Cross-sectional studies were assessed using the 8-item JBI checklist, cohort studies using the 11-item checklist, and case–control studies using the 10-item checklist. Disagreements between reviewers were resolved through discussion, with consultation with a third reviewer (R.M.G) when consensus could not be reached. Each checklist item was classified as “Yes”, “No”, “Unclear”, or “Not applicable”. For descriptive assessment, “Yes” responses were assigned 1 point, whereas “No” and “Unclear” responses were assigned 0 points. Not Applicable items were excluded from the denominator when calculating the proportion of applicable criteria satisfied. The resulting percentages were categorized as Good (≥75%), Fair (50–74%), or Poor (<50%) according to predefined thresholds. These categories were used descriptively to summarize methodological quality and were not used to exclude studies or assign differential weights in the meta-analysis. The completed appraisal results were recorded in a structured Supplementary File S1. All 37 eligible reports underwent critical appraisal; 36 unique datasets contributed to quantitative synthesis because the overlapping Alwakeel (2009) [19] report was excluded from all meta-analyses.

### 2.9. Statistical Plan

Publication bias and potential small-study effects were assessed using visual inspection of funnel plots and statistically evaluated using Begg’s rank correlation test and Egger’s regression test for funnel plot asymmetry. Funnel plot asymmetry was considered suggestive of potential small-study effects and possible publication bias and was interpreted alongside the results of the statistical tests. For both Begg’s and Egger’s tests, a *p*-value < 0.10 was considered indicative of statistically significant funnel plot asymmetry. The trim-and-fill results were used as a sensitivity analysis to assess the robustness of the pooled estimates to potential missing studies.

Pooled prevalences were estimated using a random-intercept logistic regression model, a generalized linear mixed model (GLMM) with a logit link, fitted with the meta package (metaprop, sm = “PLOGIT”, method = “GLMM”), which calls metafor::rma.glmm. This model analyses the study-level event counts and denominators directly under a binomial distribution and therefore does not require continuity correction or a variance-stabilizing transformation. It handles studies with zero or very low event counts and very small or very large proportions better than inverse-variance logit pooling. Between-study variance (τ^2^) was estimated using restricted maximum likelihood (REML), and confidence intervals and moderator tests used the Knapp–Hartung adjustment. Results were backtransformed to proportions for presentation. All models used complete cases for the variables required by that analysis; the number of contributing studies is reported for every pooled estimate and meta-regression model. A 95% prediction interval was calculated when estimable to describe the expected range for the true prevalence in a comparable future study. Random-effects models were used for all outcomes irrespective of the statistical significance of Cochran’s Q because clinical and methodological heterogeneity was anticipated a priori.

The heterogeneity was assessed using Cochran’s Q, I^2^, and Tau^2^ statistics to assess the percentage of overall variation across studies due to heterogeneity, which was categorized as low (0% to 25%), moderate (26% to 75%), or substantial (76% to 100%). A leave-one-out sensitivity analysis was conducted. This involved systematically removing one study at a time and recalculating the pooled proportion to evaluate the influence of individual studies on the overall results.

A sensitivity analysis was conducted by identifying and removing influential studies or potential outliers from the meta-analysis. The pooled estimates were recalculated after excluding these studies, and a new forest plot was generated to compare the pooled estimates before and after their removal. This analysis was used to assess the robustness of the pooled estimates to the influence of individual studies.

Influence diagnostics were additionally generated to quantify the impact of each individual study on the pooled estimate and on model fit. For each study, we calculated and plotted studentized residuals (rstudent), difference in fits (dffits), Cook’s distance (cook.d), the covariance ratio (cov.r), leave-one-out heterogeneity statistics (tau2.del and QE.del), leverage (hat), and study weight (weight). Studies were considered potentially influential if they exceeded the thresholds indicated by the dashed horizontal lines in the diagnostic plots (e.g., absolute studentized residuals > 1.96 or 3, or Cook’s D exceeding established cut-offs).

A Baujat plot was used to identify potentially influential studies based on their contribution to overall heterogeneity and their influence on the pooled effect estimate. Influential studies identified through this analysis were excluded, and the pooled estimates were recalculated.

Subgroup analyses were conducted to explore potential sources of heterogeneity according to study quality, study design, geographical region within Saudi Arabia, and diagnostic modality. Studies were grouped according to their methodological quality, study design (e.g., cross-sectional, cohort, or case–control), region of Saudi Arabia, and the diagnostic modality used to ascertain each complication (e.g., fundus photography/imaging vs. clinical examination for retinopathy; albuminuria- vs. estimated glomerular filtration rate (eGFR)-based definitions for nephropathy; validated instruments vs. clinical examination for neuropathy; ankle-brachial index (ABI) < 0.9 vs. other thresholds for PAD). Pooled estimates were calculated separately for each subgroup and compared to assess whether the observed prevalence and associations varied across these characteristics.

Meta-regression was performed to examine whether study-level characteristics were associated with variation in the pooled prevalence of microvascular and macrovascular complications. Potential moderators included continuous study-level variables-mean HbA1c, mean diabetes duration, mean age, female proportion, obesity prevalence, hypertension prevalence, dyslipidemia prevalence, smoking prevalence, and year of publication-and categorical variables-geographical region within Saudi Arabia, study design, risk-of-bias category (JBI quality rating), and diagnostic modality used to ascertain each complication.

Study-level association for the parsimonious multivariable meta-regression was selected in a data-driven, four-step procedure. First, univariate meta-regressions were fit for all available moderators, and any study-level association with *p* < 0.10 was flagged as a candidate. Second, categorical moderators (region, study design, JBI tool, and risk of bias) were excluded from the multivariable step to avoid multicollinearity and reference-level complications; only the nine continuous predictors remained eligible. Third, any candidate with more than 30% missing value was dropped to prevent model instability from complete-case collapse. Fourth, if two or more study-level associations survived these filters, they were combined into a single multivariable model; if exactly one survived, it was used alone; if none survived, the model defaulted to publication year as a reference.

All statistical analyses were performed using R version 4.3.2 in RStudio (version 2023.12.1+402, R Foundation for Statistical Computing, Vienna, Austria). Meta-analyses and meta-regression analyses were conducted using the metafor and meta packages. Forest plots, funnel plots, Baujat plots, and sensitivity analyses were also performed using these packages, as appropriate.

## 3. Results

Study Selection

**Figure 1:**
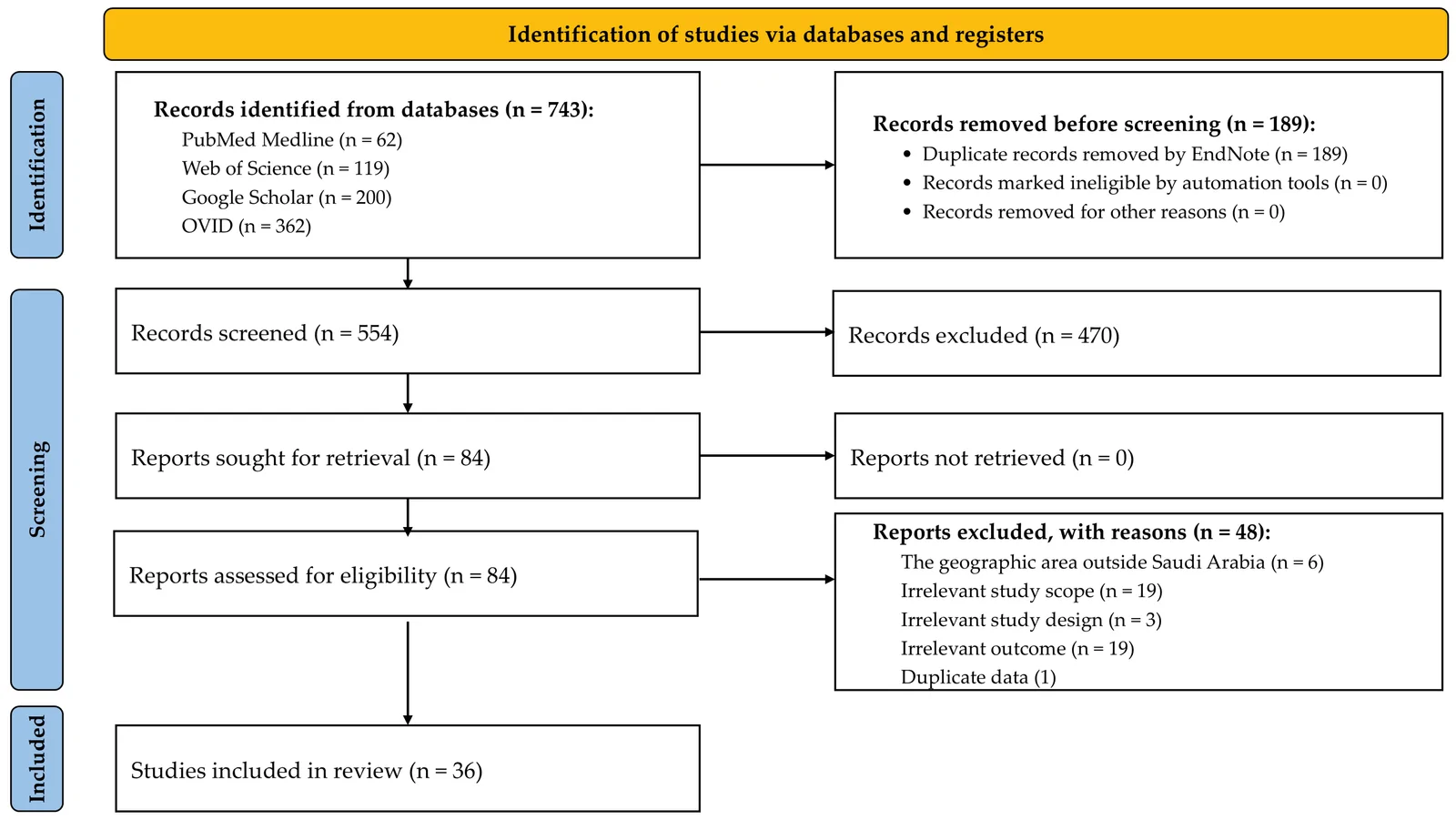
PRISMA flow diagram showing the identification, screening, eligibility assessment, and inclusion process.

A total of 743 records were identified through PubMed (*n* = 62), Google Scholar (*n* = 200), Web of Science (*n* = 119), and Ovid (*n* = 362). Before screening, 189 duplicate or automation-flagged records were removed, leaving 554 records for title and abstract screening. Of these, 470 were excluded as irrelevant. Eighty-four reports were sought out and retrieved for full-text assessment. Forty-eight full-text reports were excluded because of ineligible scope, outcome, design, population, or setting, or duplicates; the number excluded for each reason is reported in the PRISMA flow diagram. Figure 1 summarizes the study selection process. Thirty-seven reports met the inclusion criteria. Because Alwakeel (2009) [19] was judged to overlap with the population reported by Alwakeel (2008) [20], it was excluded from every quantitative synthesis; therefore, 36 unique, non-overlapping datasets contributed to at least one meta-analysis.

**Risk of Bias Assessment of Included Studies**

**Figure 2:**
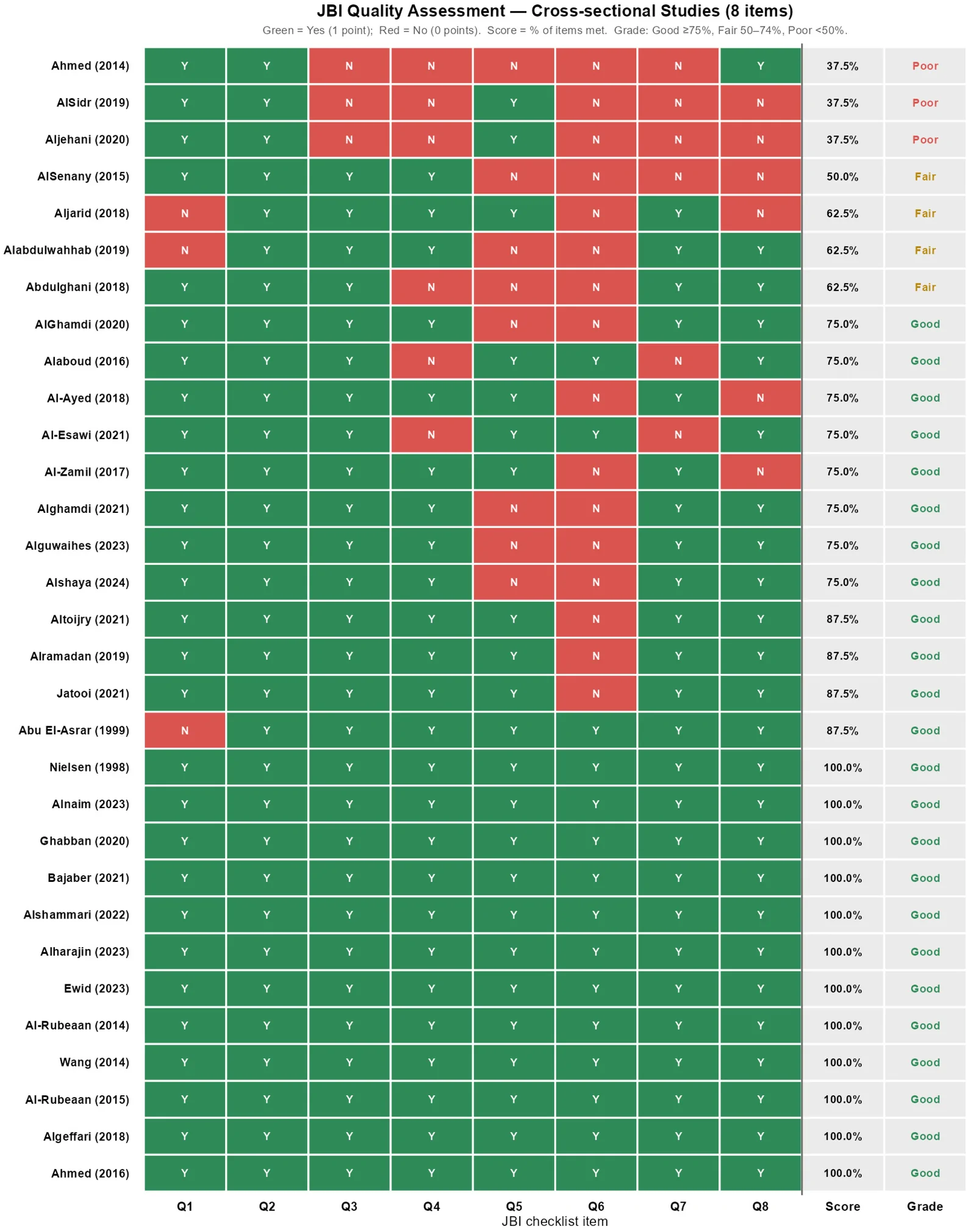
Risk of bias assessment of cross-sectional studies using the Joanna Briggs Institute (JBI) critical appraisal checklist (8 items) [6,7,11,12,13,14,16,21,22,23,24,25,26,27,28,29,30,31,32,33,34,35,36,37,38,39,40,41,42,43,44].

Cross-sectional studies (31 reports): There was a considerable variation in methodological quality across the included studies. Of the studies assessed, 24 (77.4%) were rated as having good methodological quality, four (12.9%) as fair, and three (9.7%) as poor. The quality scores ranged from 37.5% to 100%. The three studies classified as poor were Ahmed (2014) [21], AlSidr (2019) [22], and Aljehani (2020) [23], each scoring 37.5%. Four studies were classified as fair, with scores ranging from 50.0% to 62.5%. These included AlSenany (2015) [24], Aljarid (2018) [25], Alabdulwahhab (2019) [26], and Abdulghani (2018) [27]. Eight studies achieved scores of 75%, four achieved 87.5%, and twelve achieved 100%. Across the checklist items, Q1 and Q2 were met consistently by most studies. In contrast, Q6 showed the greatest pattern of non-compliance, particularly among studies with lower quality scores. Q4 and Q5 also showed some variation across studies (Figure 2).

**Figure 3:**
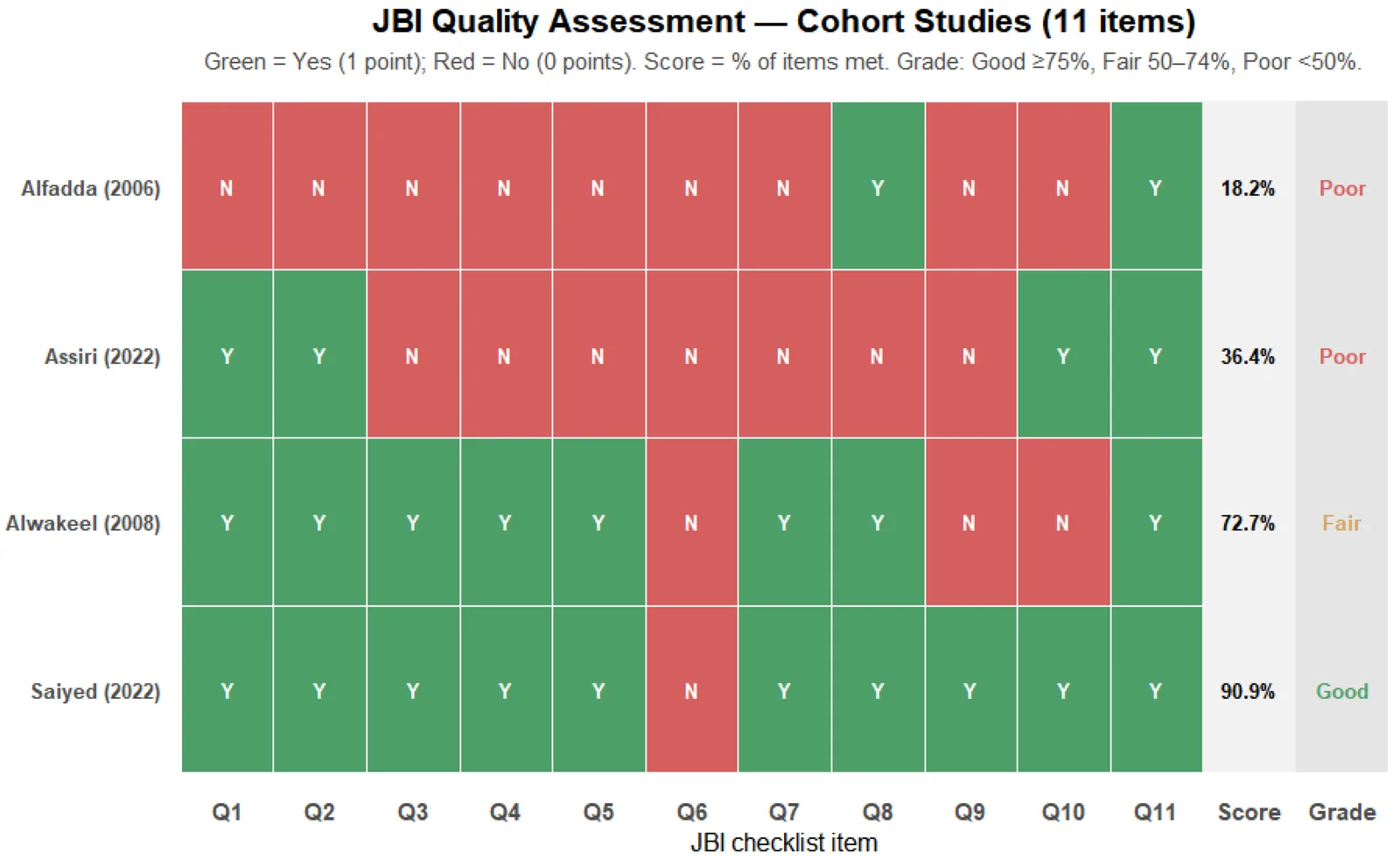
Risk of bias assessment of cohort studies using the Joanna Briggs Institute (JBI) critical appraisal checklist (11 items) [20,46,47,48].

Cohort studies (4 reports) and case–control study (1 report): Four reports were classified as retrospective cohort studies and were appraised with the 11-item JBI cohort checklist; Bokhary (2019) [45] was appraised with the 10-item JBI case–control checklist. Quality varied across the cohort reports. Saiyed (2022) [46] received a good rating (90.9%); Alwakeel (2008) [20] was rated as fair where 50–74% of applicable criteria were met; lower-scoring reports were rated Poor. Alwakeel (2009) [19] was appraised because it met qualitative eligibility criteria but was excluded from all quantitative syntheses owing to probable population overlap with Alwakeel (2008) [20] (Figure 3).

**Figure 4:**
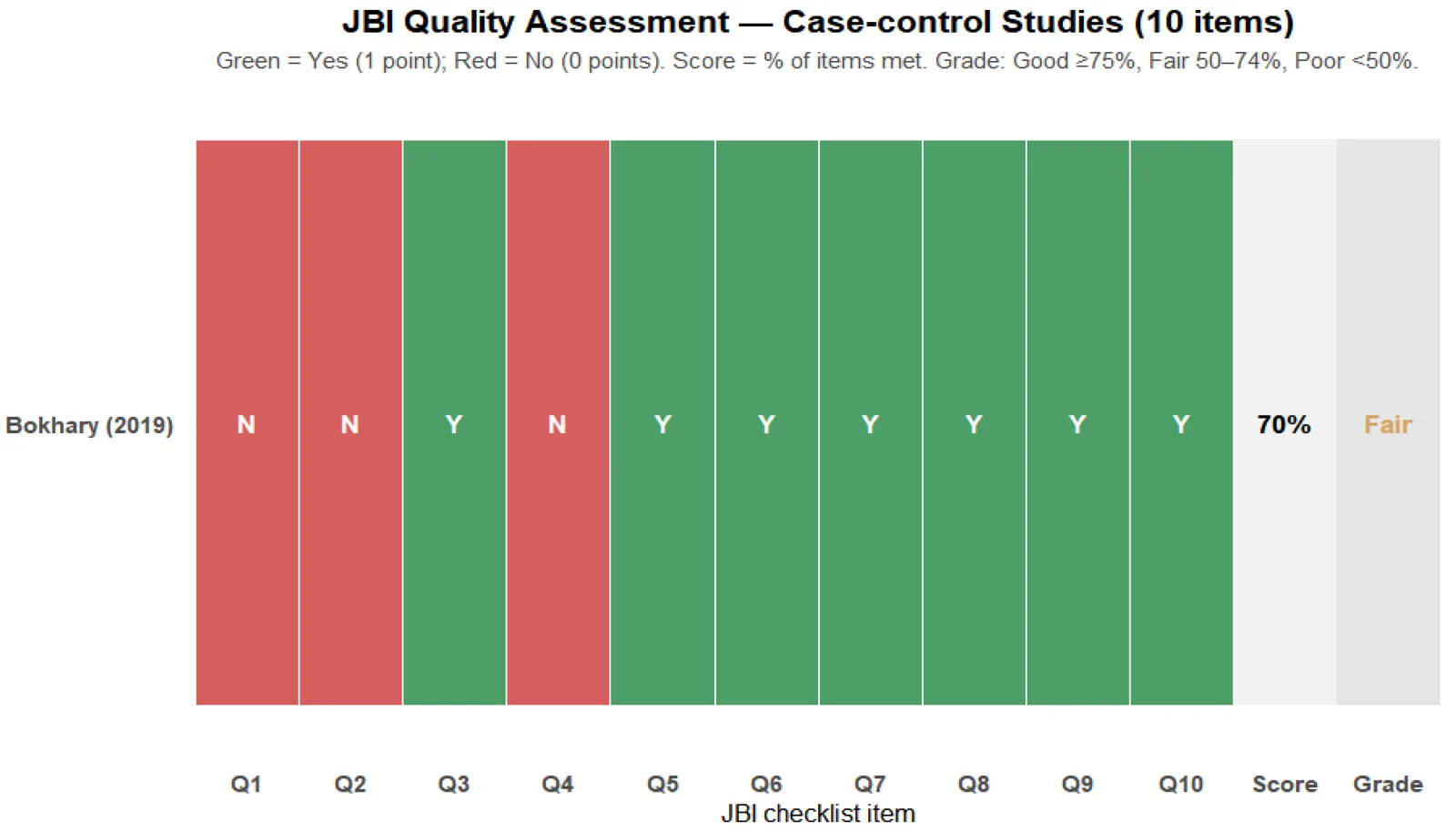
Risk of bias assessment of the case–control study using the Joanna Briggs Institute (JBI) critical appraisal checklist (10 items) [45].

**Case–Control:** Bokhary (2019) [45] achieved a “Fair” rating with a score of 70.0%. Its performance was mixed; while it successfully met seven of the ten criteria, it failed the initial three items (Q1, Q2, and Q4), which typically relate to the fundamental definitions of cases and controls and the selection process, while the overall grade was “Fair” (Figure 4).

**Study Characteristics**

Thirty-six eligible reports were included in the qualitative review. The evidence base comprised cross-sectional, retrospective cohort, and case–control designs; the design assigned to each report and the corresponding JBI checklist are shown in Table 1 and the quality-assessment figures. Across the 36 reports, 121,980 participant records were described before outcome-specific overlap and eligibility restrictions. Accordingly, outcome-specific study and participant totals, not the overall total, are used for each pooled analysis.

**Sociodemographic Parameters of Included Participants**

Table 1 shows the sociodemographic parameters of the included participants. Notably, the mean of study-level age means was approximately 57.2 ± 4.2 years, with the individual study means spanning from 48.5 years in Alharajin (2023) [28] to 64.5 ± 15 years in AlGhamdi (2020) [29]. Notably, gender was explicitly reported for most included studies (reported in 33 studies). Of those, 67,180 were men (55.1%), and 54,817 were women (44.9%). Most of the studies presented mixed-sex cohorts, but one investigation by Al-Esawi (2021) [30] consisted of entirely male participants. The single smallest diabetic cohort was Alsidr (2019) [22] with 26 adults (59.7 ± 6.4 years), while the Saudi National Diabetes Registry analyses by the Al-Rubeaan (2014) [6] and the Al-Rubeaan (2015) [16] contributed the largest gender-balanced populations with 54,670 and 50,464 patients, respectively, anchoring the overall estimates. For the Al-Rubeaan (2015) [16] cohort of 50,464 patients, men accounted for 28,238 (56.0%) and women for 22,226 (44.0%). In the Al-Rubeaan (2014) [6] registry analysis of 54,670 patients, men numbered 30,342 (55.5%) while women numbered 24,328 (44.5%).

**Comorbidity Burden**

Regarding BMI, data were available from 16 studies (N = 112,686). The weighted mean prevalence of obesity (BMI ≥ 30 kg/m^2^) was 48.0%, peaking at 71.8% in Aljehani, 2020 [23] and reaching its lowest at 13.4% in Bokhary (2019) [45]. Among the studies reporting a full BMI distribution, normal-weight (BMI 18.5–24.9 kg/m^2^) and overweight (BMI 25–29.9 kg/m^2^) categories accounted for notable proportions for instance, 18.0% and 33.8%, respectively, in the large-scale national registry by Al-Rubeaan et al. (2014) [6], while obesity (≥30 kg/m^2^) consistently emerged as the dominant weight category across the majority of cohorts. Furthermore, broader clinical analyses were presented in Al-Rubeaan (2014) [6]. Hypertension was a major comorbid condition across the reviewed studies, with prevalence rates varying widely. Proportions ranged from a low of 7.0% in Alharajin (2023) [28] (and 7.9% in Algeffari (2018) [31]) to 77.8% in Al-Ayed (2019) [12], and up to 78.1% in Alwakeel (2008) [20]. Furthermore, prevalence frequently exceeded 70% in specialized or tertiary-care cohorts such as Alnaim (2023) [32] (71.9%), Aljehani (2020) [23] (76.3%), AlSidr (2019) (76.9%) [22], and Al-Ayed (2019) [12] (77.8%), underscoring a heavy vascular comorbidity burden in these populations. Tobacco smoking was reported in 18 studies, with a weighted mean prevalence of 7.6% and a wide range from 3.0% in Alghamdi (2021) [33] to 47.0% in Al-Jarid (2018) [25]. Smoking prevalence ranged from a low of 3.0% in Alghamdi (2021) [33] and 4.0% in Nielsen, 1998 [34], up to notable highs of 30.8% in Al-Esawi (2021) [30] and 47.0% in Aljarid (2018) [25]. Physical inactivity was reported in only 4 studies. Nonetheless, sedentariness was substantial where measured, ranging from 9.6% in Aljehani (2020) [23] and 17.9% in Alguwaihes (2023) [35] up to 63.1% in Ahmed (2014) [21] and 74.1% in Al-Esawi (2021) [30]. Dyslipidemia was reported across multiple studies, with study-level proportions ranging from 20.2% in Alharajin (2023) [28] and 20.3% in Alguwaihes (2023) [35] up to 86.3% in Alnaim (2023) [32] and 81.0% in Ewid (2023) [36]. Furthermore, cardiovascular disease was documented in select studies, with reported prevalence ranging from 9.6% in Aljehani (2020) [23] up to 54.0% in Alaboud (2016) [37] and 49.1% in AlGhamdi (2020) [29].

**Different Epidemiological Parameters of T2DM-Related Variables**

Diabetes duration was reported across the studies, with numeric means or medians provided in multiple cohorts. Study-level means ranged from 6.2 years in Aljehani (2020) [23] to 17.33 ± 4.48 years in Al-Shammari (2022) [39]. Several studies also reported categorical distributions, with a substantial proportion of patients having a disease duration greater than 10 years (reaching up to 85.2% in Al-Ayed (2019) [12]. Glycemic control was assessed via HbA1c values across multiple studies, with mean levels generally reflecting poor control ranging from 8.04 in Al-Naim (2023) [32] up to 10 in Al-Shammari (2022) [39]. Categorized reporting confirmed that up to 54.1% of patients achieved guideline targets (≤7%), while ‘highly uncontrolled’ strata (>9%) frequently exceeded 40% of the sample, reaching 58.3% in the painful-neuropathy group studied by Al-Geffari (2018) [31], and 50.5% in Alfadda (2006) [47].

**Diagnostic Criteria and Assessment Methods**

In terms of diagnostic criteria, assessment methods varied but followed recognized clinical standards: retinal complications were evaluated through clinical examinations and fundus grading (e.g., Abu El-Asrar (1999) [38]; Al-Rubeaan (2015) [16]); neuropathy was assessed using tools such as the Michigan Neuropathy Screening Instrument (e.g., Al-Geffari (2018) [31]); nephropathy was tracked via albuminuria and eGFR thresholds per American Diabetes Association (ADA) criteria (e.g., Al-Rubeaan (2014) [6]); and PAD was measured via the ankle-brachial or toe-brachial index (e.g., Altoijry (2021) [41]) (Table 2).

**Risk Factors of Microvascular Complications**

**Diabetic Retinopathy [Table 3]**

Across studies, the most frequently reported risk factor for diabetic retinopathy was poor glycemic control, documented in 13 studies including studies (Abdulghani (2018) [27], Abu El-Asrar (1999) [38], Ahmed (2016) [7], Al-Esawi, 2021 [30], Al-Zamil (2017) [11], Al-Rubeaan (2014) [6], Al-Rubeaan (2015) [16], Alaboud (2016) [37], Alabdulwahhab (2019) [26]; Aljehani (2020) [23], Bajaber (2021) [14], Bokhary (2019) [45], and Alghamdi (2021) [33]). This was closely followed by longer diabetes duration, also reported in nine studies (Ahmed (2016) [7], Al-Esawi (2021) [30], Al-Rubeaan (2014) [6], Al-Rubeaan (2015) [16], Aljehani (2020) [23], Abu El-Asrar (1998) [38], Abdulghani (2018) [27], Bajaber (2021) [14], and Alramadan (2019) [13]). Older age (>60 years) was reported in four studies (Abu El-Asrar (1999) [38], Al-Esawi (2021) [30], Alramadan (2019) [13], Aljehani (2020) [23]), while hypertension and insulin use were each identified in multiple studies (hypertension: Al-Rubeaan (2014) [6], Ahmed (2016) [7], Alramadan 2019 [13]; insulin use: Abu El-Asrar (1999) [38], Alramadan 2019 [13]). Coexisting nephropathy or neuropathy was reported in three studies (Al-Rubeaan (2015) [16], Ahmed (2016) [7], Ewid (2023) [36]). Obesity or dyslipidemia were reported in three studies (Al-Rubeaan (2015) (protective) [16], Alghamdi (2021) [33], Ewid (2023) [36]), while male sex or smoking was noted in two studies (Al-Rubeaan (2014) [6], Al-Rubeaan (2015) [16]).

**Diabetic nephropathy**

The most frequently reported determinant factors for diabetic nephropathy included longer diabetes duration and poor glycemic control, documented across three cohorts (e.g., Al-Rubeaan (2014) [6], Alramadan (2019) [13], Al-Esawi (2021) [30]). Hypertension and older age were also identified as major contributors (hypertension: Alramadan (2019) [13]; older age: Aljehani (2020) [23]). Furthermore, coexisting microvascular complications such as retinopathy or neuropathy were highlighted (Al-Rubeaan (2014) [6], Ahmed (2016) [7]), alongside metabolic and lifestyle factors including dyslipidemia, insulin use, and physical inactivity (Alramadan (2019) [13]).

**Diabetic peripheral neuropathy (DPN)**

The most frequently reported risk factors for diabetic peripheral neuropathy were longer diabetes duration and poor glycemic control, each documented across multiple studies (duration: Nielsen (1998) [34], Alharajin (2023) [28], Jatooi (2021) [43]; glycemic control/hyperglycemia: AlSidr (2019) [22], Al-Esawi (2021) [30], Alramadan (2019) [13]). Obesity or central obesity, dyslipidemia, smoking, and older age or male sex were each reported in multiple cohorts (obesity: Wang (2014) [44], Alramadan (2019) [13]; dyslipidemia: Alshammari (2022) [39]; smoking: Alramadan (2019) [13], Al-Esawi (2021) [30]; age or sex: Alshammari (2022) [39], Alramadan (2019) [13], Nielsen (1998) [34]). Medication non-adherence was noted in two studies (Alharajin (2023) [28], Algeffari (2018) [31]), while high fasting glucose was reported in two studies (Wang (2014) [44], Jatooi (2021) [43]).

**Table 3:** Risk factors of diabetic microvascular complications in Saudi Arabia.

Author	Key Microvascular Risk Factors
**Abdulghani, 2018 [27]**	Retinopathy: highly uncontrolled HbA1c; diabetes duration > 10 years.
**Abu El-Asrar, 1999 [38]**	Retinopathy: age > 60 years, insulin use, diabetes duration > 10 years, poor glycemic control, nephropathy.
**Ahmed, 2016 [7]**	Retinopathy: older age, younger age at onset, longer disease duration, poor glycemic control, hypertension, insulin use, neuropathy, nephropathy. Nephropathy: retinopathy.
**Al-Esawi, 2021 [30]**	Retinopathy, nephropathy, neuropathy: uncontrolled HbA1c, age > 60 years, smoking, physical inactivity, diabetes duration.
**Al-Rubeaan, 2014 [6]**	Retinopathy: diabetes duration, hypertension, hyperlipidemia, poor glycemic control, age > 45 years, male sex, smoking. Nephropathy: diabetes duration, retinopathy, neuropathy, hypertension, age > 45 years, hyperlipidemia, male sex, smoking, poor glycemic control.
**Al-Rubeaan, 2015 [16]**	Overall: diabetes duration, age, nephropathy, neuropathy, insulin use, poor glycemic control, hypertension, male sex. Retinopathy: additional smoking, hyperlipidemia, obesity reported as protective.
**Al-Zamil, 2017 [11]**	Retinopathy: uncontrolled HbA1c.
**Alabdulwahhab, 2019 [26]**	Overall/retinopathy: high HbA1c.
**Alaboud, 2016 [37]**	Overall, retinopathy, nephropathy, neuropathy: age, diabetes duration, HbA1c, controlled hypertension.
**Algeffari, 2018 [31]**	Neuropathy: high HbA1c, poor treatment compliance.
**AlGhamdi, 2020 [29]**	Neuropathy: higher HbA1c, higher blood glucose.
**Alghamdi, 2021 [33]**	Retinopathy: high HbA1c and LDL.
**Alharajin, 2023 [28]**	Neuropathy: treatment non-compliance, female sex, comorbidities, diabetes duration > 15 years.
**Aljehani, 2020 [23]**	Overall: age > 60 years, diabetes duration > 10 years, HbA1c > 7%. Retinopathy: age, diabetes duration, high systolic blood pressure. Nephropathy/neuropathy: age, diabetes duration.
**Alnaim, 2023 [32]**	Overall: older age (>55 years), retinopathy screening, injectable therapy.
**Alramadan, 2019 [13]**	Retinopathy: longer diabetes duration, age > 70 years, hypertension, low physical activity, insulin use, lower education, family history of diabetes. Nephropathy: hypertension, low HDL, high triglycerides, prolonged sitting, insulin monotherapy, older age. Neuropathy: smoking, low physical activity, prolonged sitting, obesity, HbA1c ≥ 9%, low income, male sex, family history, insulin use.
**Alshammari, 2022 [39]**	Neuropathy: male sex, fasting glucose, cholesterol, triglycerides, LDL.
**AlSidr, 2019 [22]**	Overall: high glucose combined with hypertension associated with nephropathy and retinopathy. Neuropathy: magnitude and duration of hyperglycemia.
**Bajaber, 2021 [14]**	Retinopathy: diabetes duration 11–16 years, missed medications, no annual fundus examination, uncontrolled diabetes, higher BMI.
**Bokhary, 2019 [45]**	Overall: HbA1c, sex, hematocrit, history of hypertension. Retinopathy: hypertension and diabetic foot ulcer.
**Ewid, 2023 [36]**	Overall: hyperlipidemia, adherence to recommendations, physical activity. Retinopathy: hyperlipidemia, creatinine, neuropathy. Nephropathy: adherence, education, physical activity. Neuropathy: similar associations.
**Ghabban, 2020 [42]**	Overall, retinopathy, nephropathy, neuropathy: longer disease duration and combined insulin plus oral-drug use associated with poorer glycemic control and microvascular complications.
**Jatooi, 2021 [43]**	Nephropathy: high fasting blood glucose and diabetes duration > 1 year were significant predictors of DPN.
**Nielsen, 1998 [34]**	Neuropathy: longer diabetes duration, older age, lower proportion of smokers.
**Wang, 2014 [44]**	Neuropathy: longer diabetes duration, abdominal obesity, higher fasting glucose, creatinine and WBC count. Oral hypoglycemics are associated with lower odds of DPN.

HbA1c = glycated hemoglobin; LDL = low-density lipoprotein; HDL = high-density lipoprotein; BMI = body mass index; WBC = white blood cell; DPN = diabetic peripheral neuropathy. Microvascular complications include diabetic retinopathy, diabetic nephropathy, and diabetic neuropathy. Risk factors are reported as described in the respective studies. Associations described as protective or associated with lower odds are retained as reported by the authors and do not necessarily indicate causality.

**Risk Factors of Macrovascular Complications and Diabetic foot (Table 4)**

**Cerebrovascular Stroke**

The most frequently identified risk factor for cerebrovascular stroke was hypertension, reported in studies such as Alramadan (2019) [13] (alongside Alaboud (2016) [37]). Older age, male gender, and longer diabetes duration were also reported (Alramadan (2019) [13], Al-Esawi (2021) [30]). Lower educational level was reported in Alramadan (2019) [13], and poor glycemic control in Ghabban (2020) [42] and Al-Esawi (2021) [30].

**Myocardial Infarction (MI) and Broader Coronary Heart Disease/Coronary Artery Disease (CHD/CAD)**

The most frequently reported associated factors for MI and CHD/CAD included hypertension, older age, male sex, and longer diabetes duration (reported by Alramadan (2019) [13], Alaboud (2016) [37], and Ghabban (2020) [42]). Additional contributing factors noted across cohorts included insulin use (Alramadan (2019) [13]), smoking (Al-Esawi (2021) [30]), and physical inactivity/prolonged sitting (Al-Esawi (2021) [30], Alramadan 2019 [13]).

**Peripheral Arterial Disease**

The most frequently reported associated factors for PAD are older age, diabetes duration, poor glycemic control, and prolonged sitting or physical inactivity (Aljehani (2020) [23], Alramadan (2019) [13], AlSidr (2019) [22], Altoijry (2021) [41]). Additional factors included smoking, footwear style, and prior vascular disease or neuropathy (Alramadan (2019) [13], Nielsen (1998) [34], AlSidr (2019) [22]).

**Diabetic foot**

The most frequently reported associated factors for diabetic foot were hypertension, previous foot-ulcer history, and poor glycemic control (hypertension: Bokhary (2019) [45]; history of diabetic foot ulcer: Bokhary (2019) [45]; glycemic control: Alramadan (2019) [13]; Al-Esawi (2021) [30]). Longer diabetes duration was reported in one study (Alramadan (2019) [13]), while peripheral neuropathy was consistently recognized across all high-risk cohorts.

**Table 4:** Risk factors for macrovascular complications and diabetic foot disease in Saudi Arabia.

Author	Key Macrovascular Risk Factors
**Al-Esawi, 2021 [30]**	CVD, MI, stroke and diabetic foot: uncontrolled HbA1c, age > 60 years, smoking, physical inactivity, diabetes duration.
**Alaboud, 2016 [37]**	CVD, MI and stroke: age, diabetes duration, HbA1c level, controlled hypertension.
**Aljarid, 2018 [25]**	PAD: older age and history of angioplasty. Female sex, hypertension, oral hypoglycemic drugs, and smoking were also implicated, although some were not statistically significant.
**Aljehani, 2020 [23]**	Diabetic foot: age, diabetes duration, poor glycemic control.
**Alramadan, 2019 [13]**	CVD/MI: hypertension, obesity, prolonged sitting, male sex, older age, longer diabetes duration, insulin use. Stroke: hypertension, lower education, male sex, prolonged sitting. Diabetic foot: current/past/passive smoking, low physical activity, insulin use, low household income, residence in Hofuf region.
**AlSidr, 2019 [22]**	Overall: dyslipidemia contributes to macrovascular disease risk. Diabetic foot: diabetes duration, HbA1c, high blood pressure, PVD, and DPN.
**Altoijry, 2021 [41]**	Diabetic foot: old age, history of coronary artery disease or PAD in the affected limb.
**Ewid, 2023 [36]**	Overall: age (OR 1.050, *p* = 0.029) and presence of retinopathy (OR 3.745, *p* = 0.002).
**Ghabban, 2020 [42]**	Overall, CVD, MI and stroke: longer diabetes duration and combined insulin plus oral-drug use associated with poorer glycemic control and macrovascular complications.
**Nielsen, 1998 [34]**	Diabetic foot: longer diabetes duration; footwear style may be causal.

CVD = cardiovascular disease; MI = myocardial infarction; PAD = peripheral artery disease; PVD = peripheral vascular disease; DPN = diabetic peripheral neuropathy; HbA1c = glycated hemoglobin; OR = odds ratio; *p* = *p*-value. Risk factors are reported as described in the respective studies. Factors described as implicated but not statistically significant are retained where explicitly reported by the authors.

**Meta-Analysis of the Pooled Prevalence of Microvascular and Macrovascular Complications and Their Risk Factors**
**Microvascular Complications**
**Diabetic retinopathy (26 studies, 119,004 patients)**

**Figure 5:**
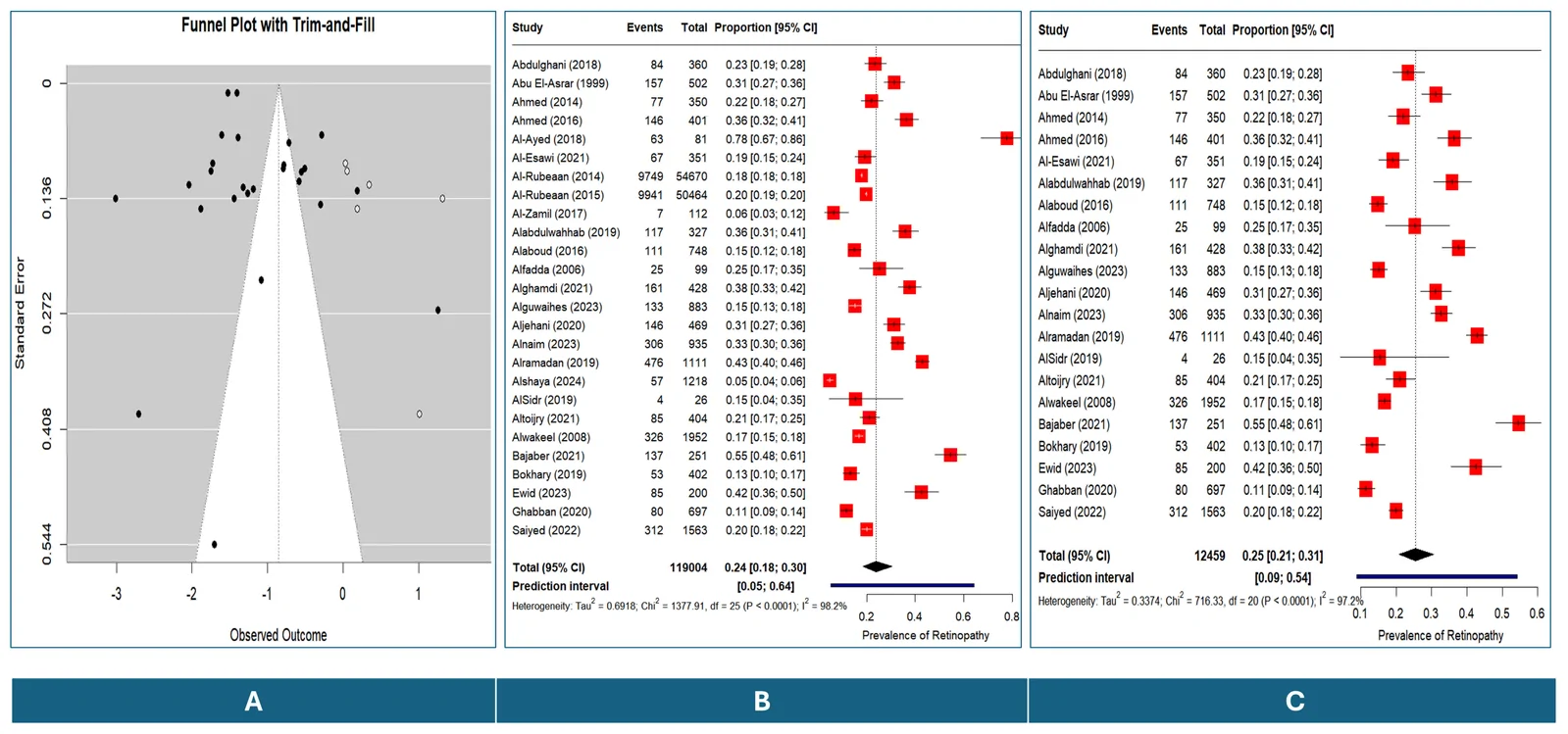
Retinopathy analyses: (**A**) Trim and fill for assessment of small-study effects, each filled black circles (●) represent individual studies included in the meta-analysis while open circles (○) represent filled or imputed studies (**B**) pooled prevalence, the vertical dotted line represents the overall pooled estimate; and (**C**) pooled prevalence after exclusion of influential outliers. Pooled proportions were estimated using an inverse-variance random-effects model with restricted maximum likelihood (REML) and Knapp–Hartung adjustment. Squares represent study estimates, horizontal lines show 95% confidence intervals, and the diamond shows the pooled proportion [6,7,11,12,13,14,16,20,21,22,23,26,27,30,32,33,35,36,37,38,40,41,42,45,46,47].

Publication bias: Supplementary Figure S1X shows the distribution of the 26 retinopathy prevalence estimates (logit-transformed) against their standard errors. The funnel plot shows considerable scatter, with most studies falling outside the 95% pseudo-confidence region. This pattern is consistent with the extreme between-study heterogeneity observed (I^2^ = 98.2%), rather than with publication bias. A small number of studies sit as notable outliers. Al-Ayed (2019) [12] appears on the far right (prevalence 77.8%, logit ≈ +1.25), which is clinically explainable: it was recruited from a diabetic foot clinic, a high-risk population. Al-Zamil (2017) [11] appears on the far left (prevalence: 6.3%, logit ≈ −2.7), consistent with a cross-sectional single-center study with only seven events in 112 patients. Despite the visual scatter, formal tests do not indicate significant asymmetry. Egger’s regression test (z = −0.433, *p* = 0.669) shows that the intercept of the regression of standardized effect on precision is statistically indistinguishable from zero. The direction of the z-statistic (negative) is not interpretable given the non-significant *p*-value. Begg’s rank correlation test (Kendall’s tau = −0.037, *p* = 0.8044) provides a second, methodologically independent confirmation: a tau of −0.037 is essentially zero, and *p* = 0.80 is far from significance. The trim-and-fill algorithm found 6 studies to be added. Visual asymmetry and wide scatter may reflect clinical and methodological heterogeneity; the nonsignificant Egger and Begg tests do not exclude publication bias or other small-study effects (Figure 5A).

Pooled effect: The pooled estimate was 24% (95% CI, 18–30%). However, heterogeneity is extremely high, with I^2^ = 98.2%, τ^2^ = 0.691, and a Q statistic of 1378 (df = 25, *p* < 0.0001). This means that virtually all the variability between studies is due to genuine between-study differences rather than to sampling error. The two mega-studies, Al-Rubeaan (2014) [6] and Al-Rubeaan (2015) [16], together contribute more than 100,000 participants and dominate the pooled estimate, pulling it toward 18–20%, while smaller studies frequently sit between 30% and 50% (Figure 5B).

Pooled effect after removing outliers (21 studies): The same analysis after removing 5 studies identified as outliers by influence diagnostics. The pooled prevalence was essentially unchanged at 25% (95% CI, 21–31%), meaning the outliers were not biasing the central estimate, only inflating its uncertainty. τ^2^ drops from 0.69 to 0.34. Nonetheless, I^2^ remains very high at 97.2%. This is the classic I^2^ paradox: I^2^ is a relative measure, and removing outliers reduces absolute heterogeneity (τ^2^) far more than it reduces the proportion of remaining variability attributed to between-study differences (Figure 5C).

Subgroup analysis: The subgroup analyses, presented in Supplementary Figure S1 (Panels A–D), reveal that the extreme heterogeneity observed in the overall pooled estimate persists across nearly all subgroups, regardless of how the studies are stratified (risk of bias, study design, region, or diagnostic method). The test for subgroup differences was not statistically significant for risk of bias (*p* = 0.0843) or diagnostic method (*p* = 0.372), but it was significant for study design and region (*p* < 0.001). However, most subgroups retained I^2^ values still substantial, suggesting that the clinical and methodological diversity across studies was too profound to be resolved by simple subgrouping. For sensitivity analysis, see Supplementary Figure S1E–H.

**Meta-regression:** None of the evaluated moderators showed a statistically significant association. These included diabetes duration, diagnostic modality, dyslipidemia, female proportion, HbA1c, hypertension, JBI score, mean age, obesity, region, risk of bias, smoking, study design, and year of publication, with all *p*-values greater than 0.05. The proportion of residual heterogeneity explained by individual moderators was generally 0%, with HbA1c and study design explaining 4.7% and 6.1% of the variance, respectively. Despite these analyses, substantial residual heterogeneity remained across all models, with residual I^2^ ranging from 98.4% to 99.9%. The parsimonious meta-regression retained year of publication as the moderator. However, year of publication was not statistically significant (β = −0.0046, 95% CI −0.0679 to 0.0586, *p* = 0.881), explained 0% of the variance, and left substantial residual heterogeneity (I^2^ = 99.7%). See Supplementary Table S1, Supplementary Figure S1I.

**Diabetic nephropathy (22 studies, 117,592 patients)**

**Figure 6:**
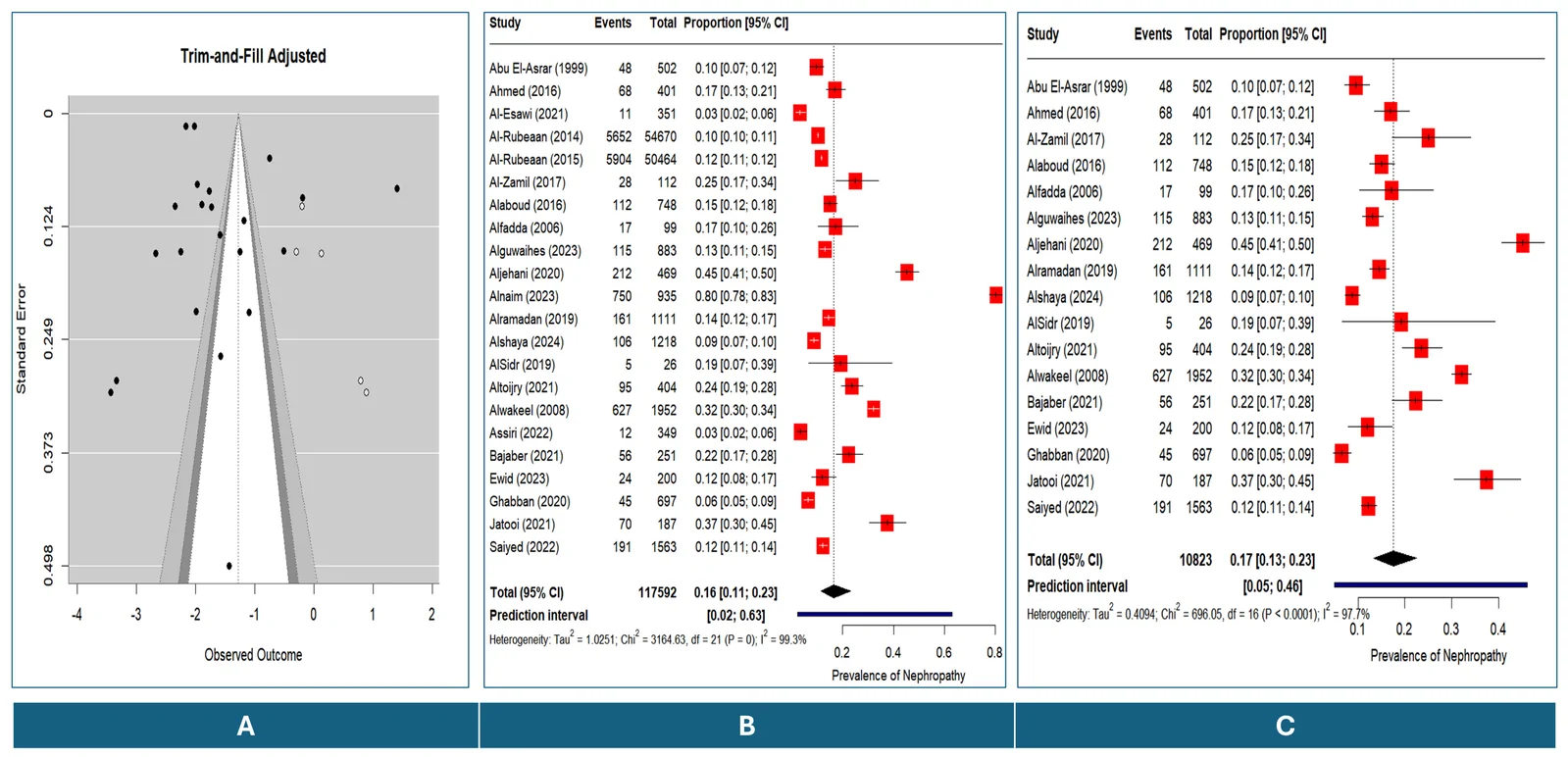
Nephropathy analyses: (**A**) Trim and fill for assessment of small-study effects; each filled black circles (●) represent individual studies included in the meta-analysis while open circles (○) represent filled or imputed studies (**B**) pooled prevalence; the vertical dotted line represents the overall pooled estimate; and (**C**) pooled prevalence after exclusion of influential outliers. Pooled proportions were estimated using an inverse-variance random-effects model with REML and Knapp–Hartung adjustment. Squares represent study estimates, horizontal lines show 95% confidence intervals, and the diamond shows the pooled proportion. The study by Alnaim (2023) [32] reported a very high prevalence of nephropathy at 80%; this study was removed as one of the influential studies [6,7,11,13,14,16,20,22,23,30,32,35,36,37,38,40,41,42,43,46,47,48].

**Publication bias:** The funnel plot displays marked asymmetry, with a dense cluster of studies on the left side and a sparse right side (Supplementary Figure S2X). Notably, the trim-and-fill algorithm identified 5 potentially missing studies on the right side of the funnel, which were imputed to restore symmetry (Figure 6A). However, formal regression-based tests did not indicate significant asymmetry. Egger’s regression test (z = −1.209, *p* = 0.24) shows that the intercept of the regression of standardized effect on precision is statistically indistinguishable from zero. The direction of the z-statistic (negative) is not interpretable given the non-significant *p*-value. Begg’s rank correlation test (Kendall’s tau = −0.126, *p* = 0.4343) provides a second, methodologically independent confirmation: a tau of −0.126 is close to zero, and *p* = 0.43 is far from significance. The discordance between the trim-and-fill result and the regression-based tests is a classic consequence of extreme heterogeneity: when I^2^ approaches 100%, the noise dominates the signal, and Egger’s and Begg’s tests lose power to detect asymmetry.

**Pooled effect:** The pooled prevalence of diabetic nephropathy across all 22 studies was 16.0% (95% CI, 11.0–23.0%). However, heterogeneity is extremely high, with I^2^ = 99.3%, τ^2^ = 1.03, and a Q statistic of 3165 (df = 21, *p* < 0.0001). This means that virtually all the variability between studies is due to genuine between-study differences rather than to sampling error (Figure 6B).

**Pooled effect after removing outliers:** The same analysis after removing studies identified as outliers by influence diagnostics. The pooled prevalence was essentially unchanged at 17.0% (95% CI, 13.0–23.0%), meaning the outliers were not biasing the central estimate, only inflating its uncertainty. The between-study variance: τ^2^ drops from 1.03 to 0.41, a modest reduction, showing that the mega-studies (Al-Rubeaan 2014 [6] and al-Rubeaan 2015 [16]) were responsible for this residual heterogeneity. Nonetheless, I^2^ remains very high at 97.7% (Figure 6C).

The subgroup analyses for diabetic nephropathy, presented in Supplementary Figure S2 (Panels 2A–2D), demonstrate that the extreme heterogeneity observed in the overall pooled estimate (I^2^ = 99%) persists across all stratified subgroups. The test for subgroup differences was statistically significant for risk of bias (*p* < 0.0001), geographic region (*p* < 0.0001), and diagnostic tool (*p* = 0.048) but not for study design (*p* = 0.166). This indicates that methodological quality, rather than study design, explains some of the between-study variance. However, nearly all subgroups retained I^2^ values above 95%, confirming that the clinical and methodological diversity across studies is too profound to be resolved by simple subgrouping. For sensitivity analysis, see Supplementary Figure S2E–H.

Meta-regression: Univariate meta-regression identified statistically significant study-level associations between the diabetic nephropathy estimate and diabetes duration (β = −0.2539, 95% CI −0.4112 to −0.0966, *p* = 0.005) and female proportion (β = 0.0357, 95% CI 0.0048 to 0.0667, *p* = 0.026). Diabetes duration explained 52.5% of the between-study variance, while female proportion explained 18.8%. No statistically significant associations were observed for diagnostic modality, dyslipidemia, HbA1c, hypertension, JBI score, mean age, obesity, region, risk of bias, smoking, study design, or year of publication (all *p* > 0.05). Despite the significant associations observed for diabetes duration and female proportion, substantial residual heterogeneity remained in all models, with residual I^2^ values ranging from 98.9% to 99.9% (Supplementary Table S2, Supplementary Figure S2I).

**Diabetic neuropathy (27 studies, 69,060 patients)**

**Figure 7:**
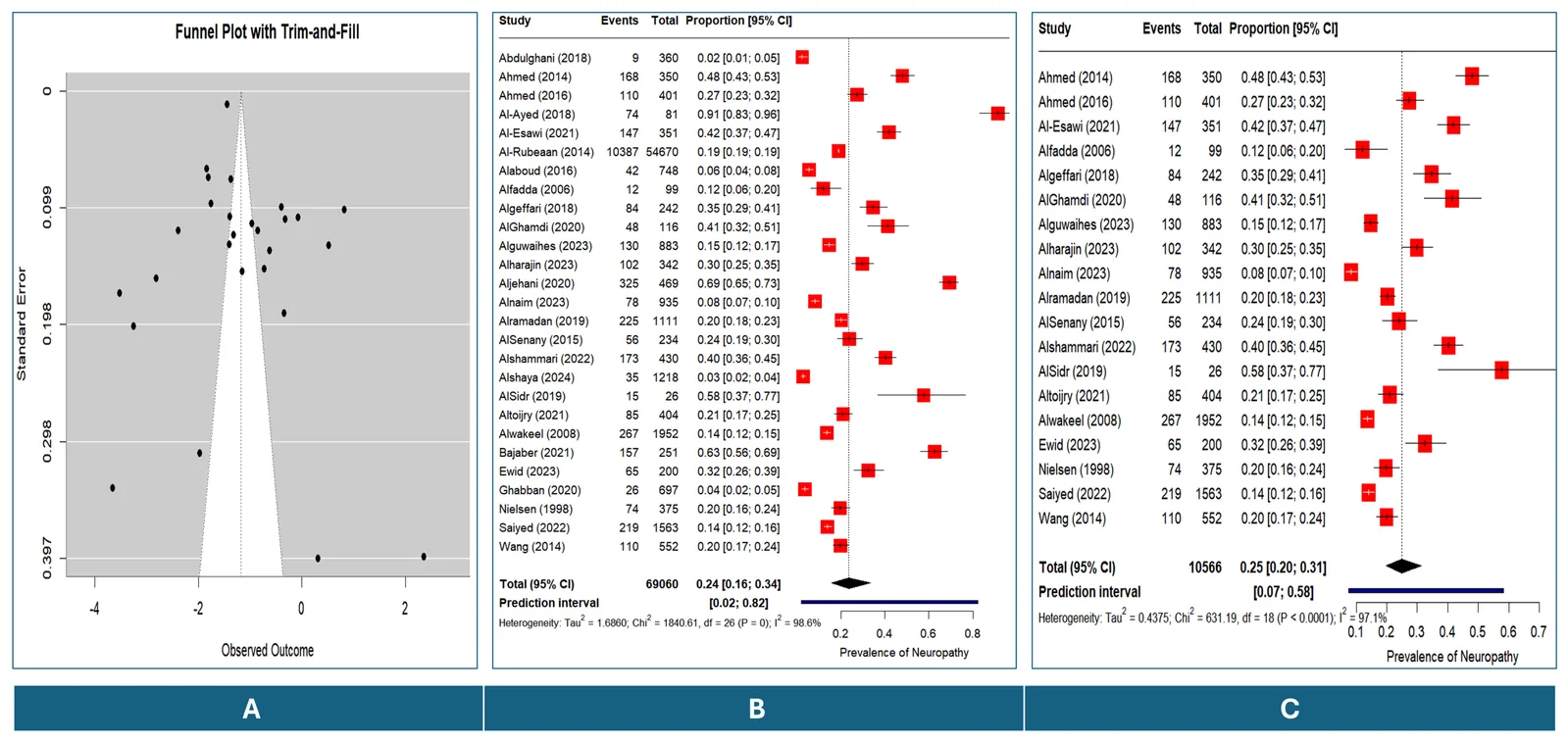
Neuropathy analyses: (**A**) Trim and fill for assessment of small-study effects; each filled black circles (●) represent individual studies included in the meta-analysis (**B**) pooled prevalence; the vertical dotted line represents the overall pooled estimate; and (**C**) pooled prevalence after exclusion of influential outliers. Pooled proportions were estimated using an inverse-variance random-effects model with REML and Knapp–Hartung adjustment. Squares represent study estimates, horizontal lines show 95% confidence intervals, and the diamond shows the pooled proportion [6,7,12,13,14,20,21,22,23,24,27,28,29,30,31,32,34,35,36,37,39,40,41,42,44,46,47].

**Publication bias:** The funnel plot appears visually asymmetric, with a dense cluster of studies on the left side and a sparser on the right-side Supplementary Figure S3X. However, formal testing does not support this observation. Egger’s regression test (z = 0.633, *p* = 0.5320) shows that the intercept of the regression of standardized effect on precision is statistically indistinguishable from zero (*p* = 0.53, well above the conventional 0.10 threshold). The trim-and-fill algorithm added no studies. This means the pooled estimate requires no adjustment for potential publication bias, and the sensitivity analysis using the unadjusted estimate is valid (Figure 7A).

**Pooled effect:** The pooled estimate of diabetic retinopathy prevalence was 24.0% (95% CI, 16.0–34.0%). However, heterogeneity was extreme, with I^2^ = 98.6%, τ^2^ = 1.622, and a Q statistic of 1841 (df = 26, *p* < 0.0001). The prediction interval (2–82%) spans nearly the entire possible range, underscoring the substantial uncertainty in predicting neuropathy prevalence in any individual setting (Figure 7B).

**Pooled effect after removing outliers:** The same analysis after removing 8 studies identified as outliers by influence diagnostics. The pooled prevalence was essentially unchanged at 25.0% (95% CI, 20.0–31.0%). Nonetheless, I^2^ remains very high at 97.0% (Figure 7C).

**The subgroup analyses:** The extreme heterogeneity observed in the overall pooled estimate (I^2^ = 98.5%) persists across all stratified subgroups Supplementary Figure S3A–D. The test for subgroup differences was not statistically significant for risk of bias (*p* = 0.5920), study design (*p* = 0.0826), diagnostic tool (*p* = 0.122), but was significant for geographic region (*p* < 0.001). Nearly all subgroups retained high I^2^ values, confirming that the clinical and methodological diversity across studies is too profound to be resolved by simple subgrouping. For sensitivity analysis, see Supplementary Figure S3E–H.

**Meta-regression:** Univariate meta-regression identified a statistically significant association between smoking prevalence and the effect estimate (β = 0.0932, 95% CI 0.0013 to 0.1852, *p* = 0.0472), explaining 18.8% of the between-study variance. The remaining evaluated moderators were not statistically significant, including (all *p* > 0.05). Residual heterogeneity remained high across all models, with residual I^2^ ranging from 99.1% to 99.7%. The parsimonious meta-regression model retained publication year as the moderator. Publication year was not significantly associated with the effect estimate (β = 0.0134, 95% CI −0.0792 to 0.1060, *p* = 0.7677). The model explained 0% of the between-study variance, with substantial residual heterogeneity remaining (τ^2^ = 1.7822, I^2^ = 99.5%) (Supplementary Table S3, Supplementary Figure S3I).

**Figure 8:**
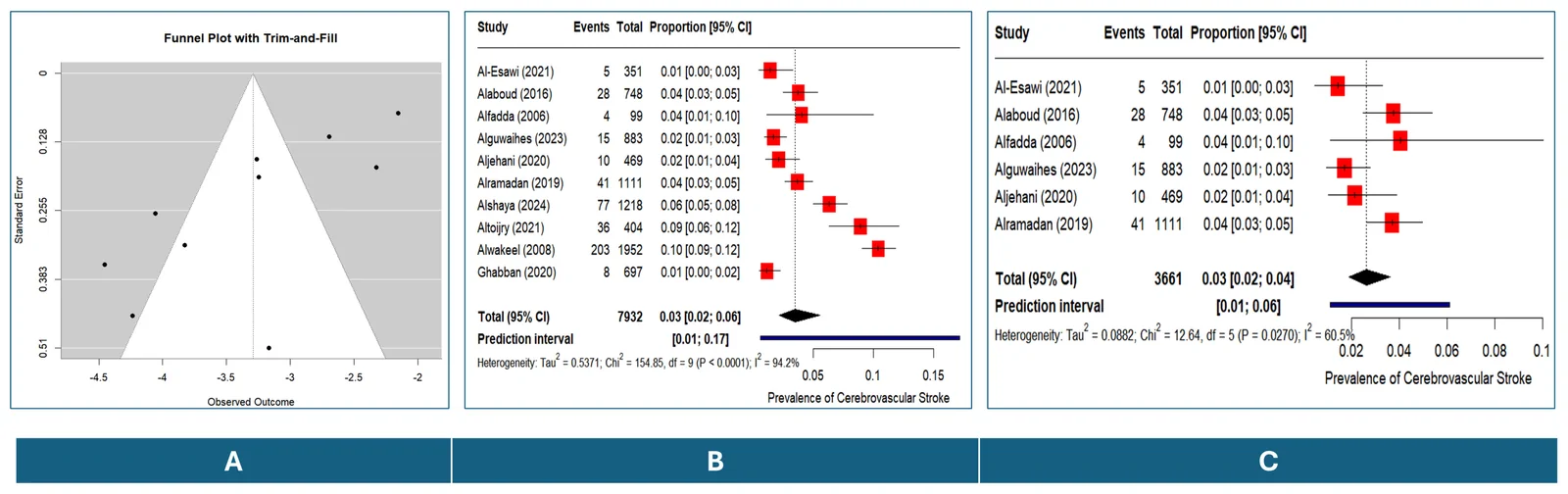
Cerebrovascular stroke analyses: (**A**) funnel plot for assessment of small-study effects; each filled black circles (●) represent individual studies included in the meta-analysis (**B**) pooled prevalence; the vertical dotted line represents the overall pooled estimate; and (**C**) pooled prevalence after exclusion of influential outliers. Pooled proportions were estimated using an inverse-variance random-effects model with REML and Knapp–Hartung adjustment. Squares represent study estimates, horizontal lines show 95% confidence intervals, and the diamond shows the pooled proportion [13,20,23,30,35,37,40,41,42,47].

**Cerebrovascular Stroke (10 Studies, 7932 patients)**
**Publication bias:** Visual inspection of the funnel plot revealed publication bias. Supplementary Figure S4X. This was confirmed by Egger’s test, which suggested statistically significant asymmetry (z = −2.879, *p* = 0.004); however, trim-and-fill imputed no studies Figure 8A.

**Pooled effect:** The pooled prevalence of cerebrovascular stroke disease across all 10 studies is presented in Figure 8B. The pooled estimate was 3.0% (95% CI, 2.0–6.0%). Heterogeneity was extreme, with I^2^ = 94.2%, τ^2^ = 0.54, and a Q statistic of 155 (df = 9, *p* < 0.0001). The prediction interval spans a wide range (1–17%). The two mega-studies, Alwakeel (2008) [20] and Alshaya (2024) [40], together contribute more than 3000 participants and dominate the pooled estimate, pulling it toward 6–10%, while smaller studies frequently sit between 1% and 4% (Figure 8B).

**Pooled effect after removing outliers:** The pooled prevalence was essentially unchanged at 3.0% (95% CI, 2.0–4.0%), compared with the unadjusted estimate, meaning the outliers were not biasing the central estimate, only inflating its uncertainty. What changes dramatically is the between-study variance: τ^2^ drops from 0.54 to 0.09, a more than six-fold reduction, showing that the mega-studies (Alwakeel 2008 [20] and Alshaya 2024 [40]) were responsible for a large share of the residual heterogeneity. Nonetheless, I^2^ remains high at 60.5%. The prediction interval also narrowed (from 0.01–0.17 to 0.01–0.06), indicating that the remaining studies are more consistent with one another, although substantial uncertainty remains (Figure 8C).

**Subgroup analysis:** The subgroup analyses demonstrate that the extreme heterogeneity observed in the overall pooled estimate persists across all stratified subgroups. The test for subgroup differences was not statistically significant for risk of bias (*p* = 0.359) or for diagnostic tool (*p* = 0.209) but was significant for study design (*p* <0.001) and geographic region (*p* = 0.007), though careful interpretation is needed because of the small number of studies per subgroup. Nearly all subgroups retained substantially high I^2^ values. Supplementary Figure S4A–D. For sensitivity analysis, see Supplementary Figure S4E–H.

**Meta-regression:** The greatest proportion of heterogeneity explained by an individual moderator was observed for HbA1c (R^2^ = 24.5%), although the association did not reach statistical significance (β = 0.7199, 95% CI −0.4663 to 1.9061, *p* = 0.167). Region explained 21.3% of the heterogeneity, while study design explained 14.6%. However, neither moderator was statistically significant. Supplementary Table S4, Supplementary Figure S4I.

**Myocardial infarction (MI) and broader coronary heart disease/coronary artery disease (CHD/CAD) (16 studies, 9352 patients)**

**Figure 9:**
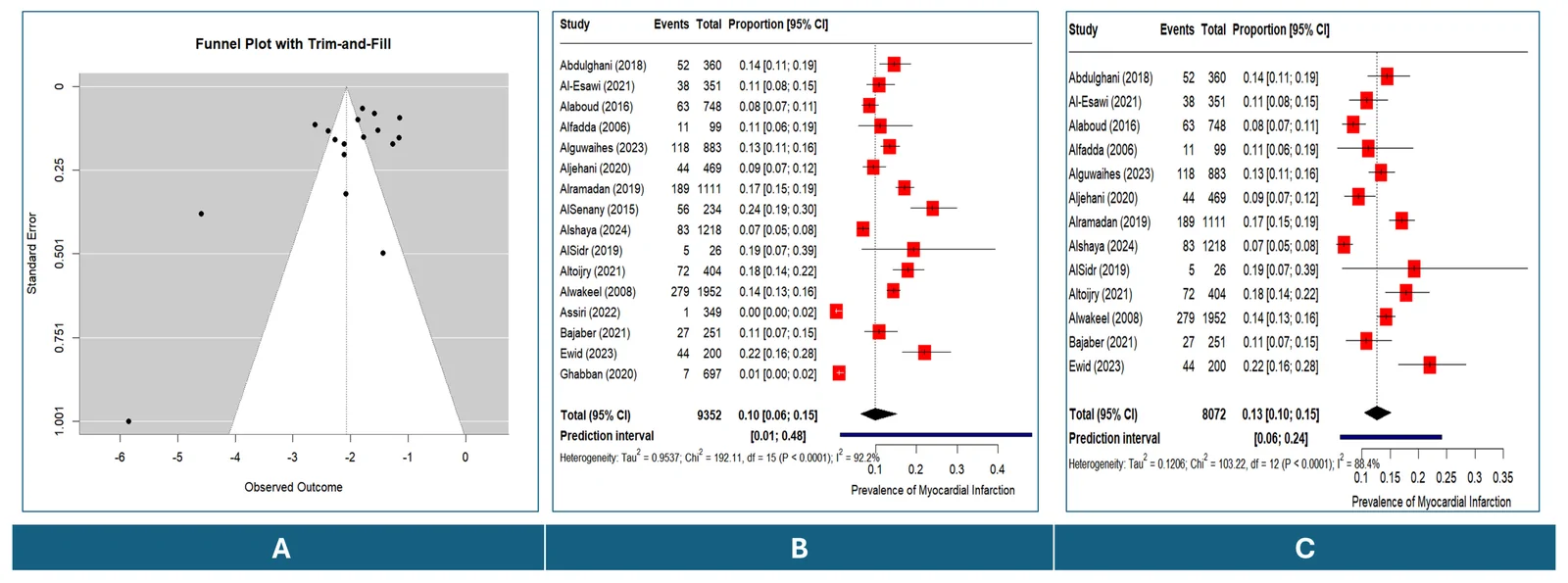
Myocardial infarction (MI) and broader coronary heart disease/coronary artery disease (CHD/CAD): (**A**) Trim and fill for assessment of small-study effects, each filled black circles (●) represent individual studies included in the meta-analysis, the vertical dotted line represents the overall pooled estimate (**B**) pooled prevalence; and (**C**) pooled prevalence after exclusion of influential outliers. Confirmed myocardial infarction was distinguished from broader CHD/CAD outcomes. Pooled proportions were estimated using an inverse-variance random-effects model with REML and Knapp–Hartung adjustment. Squares represent study estimates, horizontal lines show 95% confidence intervals, and the diamond shows the pooled proportion [13,14,20,22,23,24,27,30,35,36,37,40,41,42,47,48].

Publication bias: The funnel plot displays marked asymmetry, with a dense cluster of studies on the right side (higher prevalence) and a sparse left side. Supplementary Figure S5X. This visual asymmetry is statistically confirmed. Egger’s regression test (z = −3.058, *p* = 0.002) shows that the intercept of the standardized effect on precision is significantly different from zero. The trim-and-fill algorithm identified no potentially missing studies (Figure 9A).

Pooled effect size: The pooled prevalence of MI and CHD/CAD across all 16 studies is presented in Figure 9B. The pooled estimate was 10.0% (95% CI, 6.0–15.0%). Heterogeneity was extreme, with I^2^ = 92.2%, τ^2^ = 0.96, and a Q statistic of 192 (df = 15, *p* < 0.0001).

Pooled analysis after removing outliers: The pooled prevalence was slightly higher at 13.0% (95% CI, 10.0–15.0%), compared with the unadjusted estimate. The between-study variance (τ^2^) dropped from 0.96 to 0.12, a nearly eight-fold reduction. Nonetheless, I^2^ remains very high at 88.4%.

Subgroup analyses: They did not identify a consistent source of heterogeneity according to study design or risk-of-bias category. In contrast, a statistically significant difference was observed between regional subgroups (*p* < 0.0001), driven primarily by the difference between the Central region (11.0%) and the Western/Multi-region subgroups (24.0%). A statistically significant difference was also observed according to diagnosis (*p* = 0.003), suggesting that the clinical diagnosis of MI vs. CHD/CAD may contribute to the observed between-study heterogeneity. Supplementary Figure S5A–D. For sensitivity analysis, see Supplementary Figure S5E–H.

**Meta regression:** Univariate meta-regression was performed to assess whether study-level characteristics explained between-study heterogeneity in the prevalence of MI and CHD/CAD. No statistically significant associations were observed for year of publication, mean age, female proportion, diabetes duration, HbA1c, hypertension, obesity, smoking, dyslipidemia, JBI score, study design, risk of bias, or diagnostic modality (all *p* > 0.05). Diagnostic modality showed a modest contribution to heterogeneity (R^2^ = 6.7%), but the association was not statistically significant (β = −0.871, 95% CI −2.425 to 0.684, *p* = 0.250). The region model accounted for a substantial proportion of the observed between-study heterogeneity (R^2^ = 84.5%), with lower residual heterogeneity (τ^2^ = 0.110; I^2^ = 87.1%). In the categorical analysis, the Tabuk and Taif regional contrasts were statistically significant compared with the reference region (β = −3.325, *p* = 0.0004 and β = −4.587, *p* = 0.0023, respectively), whereas the other regional contrasts were not statistically significant. However, the overall interpretation of the regional moderator should be based on the global test for the categorical variable rather than individual regional contrasts alone. The parsimonious meta-regression retained year of publication as the only moderator. Year was not statistically significant (β = −0.032, 95% CI −0.145 to 0.081, *p* = 0.554), explained 0% of the observed heterogeneity, and substantial residual heterogeneity remained (I^2^ = 97.3%) (Supplementary Table S5, Supplementary Figure S5I).

**Peripheral arterial disease (10 studies, 5496 patients)**

**Figure 10:**
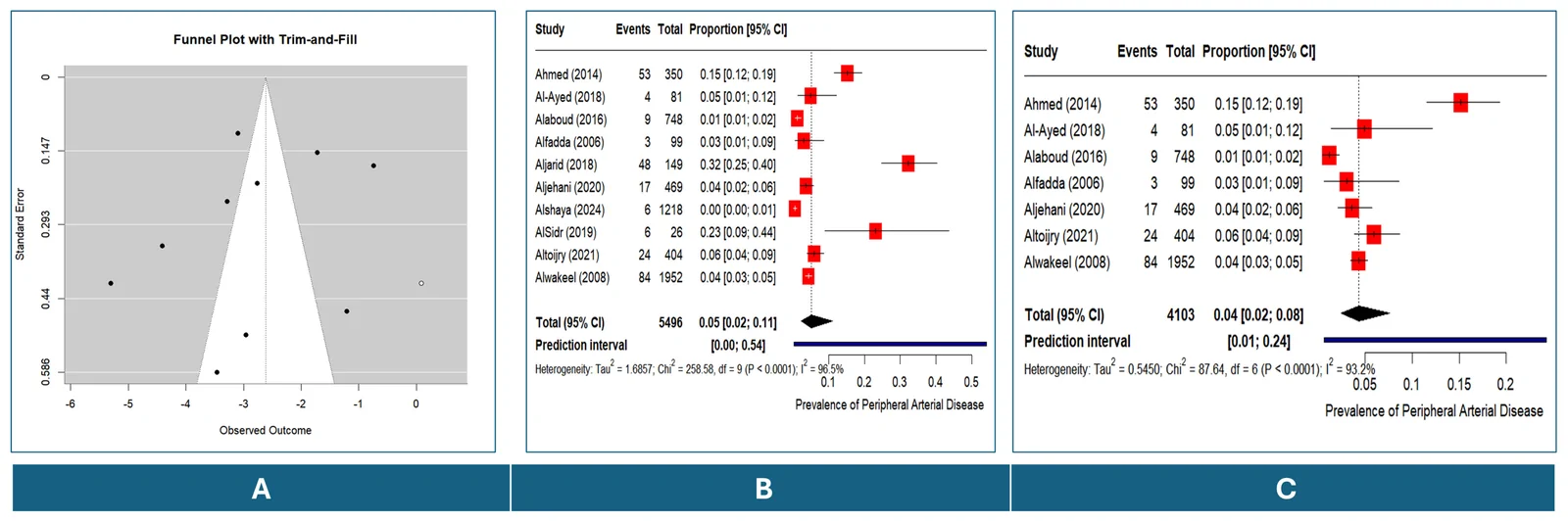
Peripheral arterial disease analyses: (**A**) Trim and fill for assessment of small-study effects, each filled black circles (●) represent individual studies included in the meta-analysis while open circles (○) represent filled or imputed studies (**B**) pooled prevalence, the vertical dotted line represents the overall pooled estimate; and (**C**) pooled prevalence after exclusion of influential outliers. Pooled proportions were estimated using an inverse-variance random-effects model with REML and Knapp–Hartung adjustment. Squares represent study estimates, horizontal lines show 95% confidence intervals, and the diamond shows the pooled proportion [12,20,21,22,23,25,37,40,41,47].

**Publication bias:** Supplementary Figure S6X shows the distribution of the 10-PAD prevalence estimates (logit-transformed) against their standard errors. Although visual inspection of the funnel plot may suggest some asymmetry, Egger’s regression test was not statistically significant (z = −0.897, *p* = 0.3961). The trim-and-fill algorithm identified 1 potentially missing study, which was imputed to restore symmetry (Figure 10A, open circle). The adjusted estimate suggests that the true pooled prevalence may be slightly lower than the unadjusted estimate (Figure 10A).

The pooled prevalence of PAD across all 10 studies is presented in Figure 10B. The pooled estimate was 5.0% (95% CI, 2.0–11.0%). Heterogeneity was extreme, with I^2^ = 96.5%, τ^2^ = 1.69, and a Q statistic of 258.58 (df = 9, *p* < 0.0001). The mega-studies, Alshaya 2024 [40] and Alwakeel 2008 [20] together contribute 3170 participants and dominate the pooled estimate, pulling it toward 0−4%, while smaller studies frequently sit between 15% and 32% (e.g., Ahmed 2014 [21]: 15.0%; Aljarid 2018 [25]: 32.0%; AlSidr 2019 [22]: 23.0%). Notably, Alshaya 2024 [40] reports a prevalence of 0.5%, which is a clear outlier on the low end (Figure 10B).

**Pooled analysis after removing outliers:** The pooled prevalence was unchanged at 4.0% (95% CI, 2.0–8.0%), compared with the unadjusted estimate, meaning the outliers were not biasing the central estimate in a clinically meaningful way, only inflating its uncertainty. The between-study variance (τ^2^) dropped from 1.67 to 0.54, a more than three-fold reduction, showing that the mega-studies (Alshaya 2024 [40] and Alwakeel 2008 [20]) and the extreme outlier (Aljarid 2018 [25], prevalence 32.0%) were responsible for a large share of the residual heterogeneity. Nonetheless, I^2^ remains high at 93.2% (Q = 88, df = 6, *p* < 0.0001) (Figure 10C).

**Subgroup analysis:** Subgroup analyses revealed a statistically significant difference by risk of bias (*p* = 0.032), with high risk of bias studies reporting a higher prevalence (11.0%) than low risk of bias studies (4.0%), and by region (*p* = 0.0048), driven entirely by a single Western study. Supplementary Figure S6A–D. For sensitivity analysis, see Supplementary Figure S6E–H.

**Meta-regression:** Univariate meta-regression showed a statistically significant association between the proportion of female participants and PAD estimates. Female proportion was negatively associated with the PAD estimate (β = −0.0842, 95% CI −0.1471 to −0.0213, *p* = 0.0150), explaining 53.2% of the between-study variance in the univariate model. This represents a study-level association and should not be interpreted as evidence of an individual-level protective effect. The other evaluated moderators, including diabetes duration, dyslipidemia, HbA1c, hypertension, mean age, obesity, smoking, JBI score, study design, risk of bias, and publication year, were not statistically significant. The overall region moderator was also not statistically significant (β= −0.9795, 95% CI −4.7798 to 2.8208, *p* = 0.5615). In the full categorical analysis, the Riyadh comparison showed a borderline association (β = −2.5720, 95% CI −5.4412 to 0.2972, *p* = 0.0718), but this did not reach conventional statistical significance (Supplementary Table S6, Supplementary Figure S6I).

**Diabetic Foot**

The pooled prevalence of diabetic foot included nine studies recruiting 5354 patients.

**Figure 11:**
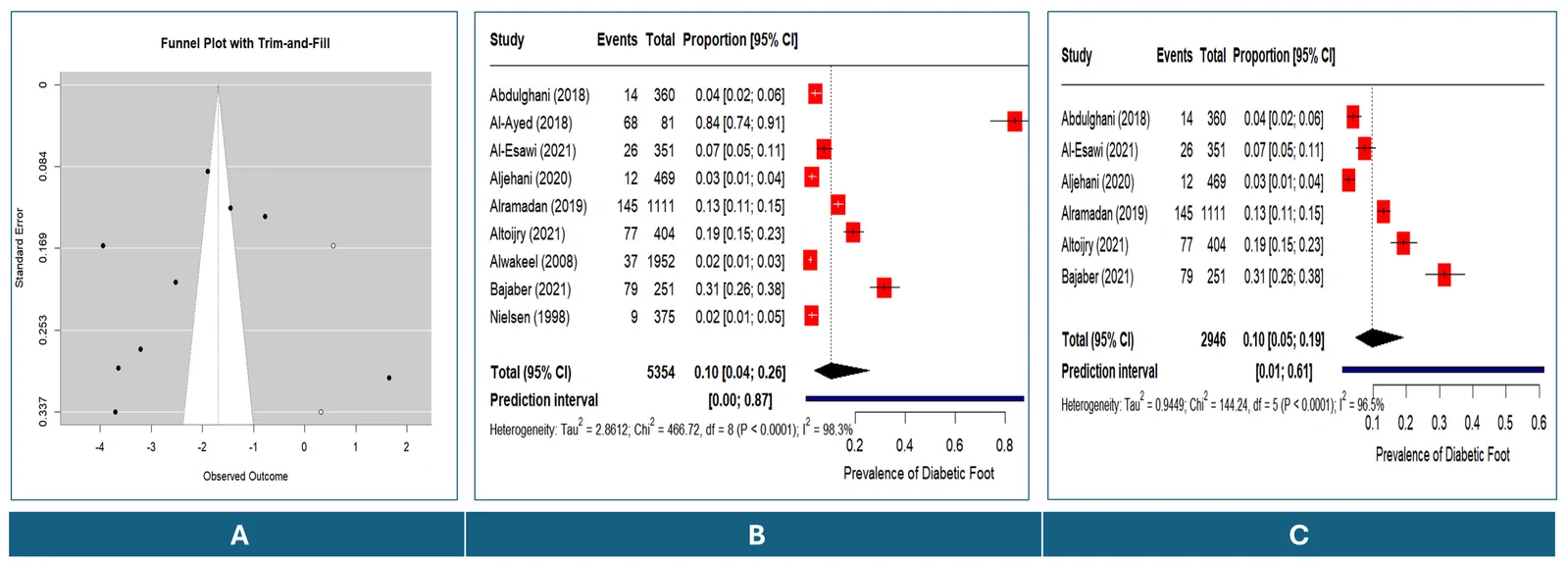
Diabetic foot disease analyses: (**A**) Trim and fill for assessment of small-study effects, each filled black circles (●) represent individual studies included in the meta-analysis while open circles (○) represent filled or imputed studies; (**B**) pooled prevalence, the vertical dotted line represents the overall pooled estimate; and (**C**) pooled prevalence after exclusion of influential outliers. Pooled proportions were estimated using an inverse-variance random-effects model with REML and Knapp–Hartung adjustment. Squares represent study estimates, horizontal lines show 95% confidence intervals, and the diamond shows the pooled proportion. Because the contributing studies included general, hospital, and specialty/high-risk clinical settings, the pooled estimate should not be interpreted as a national population prevalence. This study recruited patients with T2DM attending a diabetic foot clinic, a high-risk, specialty-care setting. Not all attendees had diabetic foot disease (68 of 81; 84.0%), so the study reported a prevalence rather than a case series and was retained. Because its estimate reflects a selected high-risk population, it is not representative of all Saudi adults with T2DM. It was identified as an extreme outlier (high-end) in the diabetic foot analysis, and a sensitivity analysis excluding it is presented [12,13,14,20,23,27,30,34,41].

Publication bias: The funnel plot displays marked asymmetry, with a dense cluster of studies on the left side (lower prevalence) and fewer studies on the right side (Supplementary Figure S7X). However, formal testing does not support statistically significant funnel plot asymmetry. Egger’s regression test (z = −0.200, *p* = 0.8463) showed that the intercept from the regression of standardized effect on precision was not significantly different from zero. The *p*-value was well above the conventional 0.10 significance threshold, providing no statistical evidence of small-study effects based on Egger’s test. The trim-and-fill algorithm identified 1 potentially missing study on the right side of the funnel, which was imputed to restore symmetry (Figure 11A, open circle). The adjusted estimate suggests that the true pooled prevalence may be slightly higher than the unadjusted estimate. However, given that only one study was imputed and the extreme heterogeneity (I^2^ = 98.3%), the adjusted estimate should be interpreted with caution.

Pooled effect: The pooled prevalence of diabetic foot across all 9 studies is presented in Figure 11B. The pooled estimate was 10.0% (95% CI, 4.0–26.0%). Heterogeneity was extreme, with I^2^ = 98.3%, τ^2^ = 2.86, and a Q statistic of 467 (df = 8, *p* < 0.0001). The prediction interval is exceptionally wide, spanning nearly the entire possible range and underscoring substantial uncertainty in predicting diabetic foot prevalence in any individual setting. The mega-study (Alwakeel (2008) [20]) contributes nearly 2000 participants and dominates the pooled estimate, pulling it toward 2–7%, while smaller studies frequently sit between 19% and 84% (e.g., Al-Ayed (2019) [12]: 84.0%; Bajaber (2021) [14]: 31.0%; Altoijry (2021) [41]: 19.0%). Notably, Al-Ayed (2019) [12] reports a prevalence of 84.0%, which is a clear outlier on the high end (Figure 11B).

Pooled effect after outlier removal: The pooled prevalence was essentially unchanged at 10.0% (95% CI, 5.0–19.0%), compared with the unadjusted estimate, meaning the outliers were not biasing the central estimate in a clinically meaningful way, only inflating its uncertainty. The between-study variance (τ^2^) dropped from 2.86 to 0.94, showing that the mega-studies (Alwakeel (2008) [20]) and the extreme outlier (Al-Ayed (2019) [12], prevalence 84.0%) were responsible for a very large share of the residual heterogeneity. Nonetheless, I^2^ remains very high at 96.5%. (Figure 11C).

Subgroup analyses identified statistically significant differences according to risk-of-bias category (*p* = 0.021) and study design (*p* = 0.001). A statistically significant difference was also observed across regional subgroups (*p* < 0.0001), driven primarily by the contrast between Riyadh (8.0%) and Makkah (31.0%). The diagnostic tool showed a borderline subgroup difference (*p* = 0.050), suggesting that differences in diagnostic methods may also contribute to the observed between-study heterogeneity. Supplementary Figure S7A–D. For sensitivity analysis, see Supplementary Figure S7E–H.

Meta-regression: None of the evaluated moderators showed a statistically significant association with the effect estimate at the conventional 0.05 level. Diabetes duration showed the largest explanatory proportion among the continuous moderators (R^2^ = 41.7%), but the association was not statistically significant (β = 0.226, 95% CI −0.322 to 0.774, *p* = 0.218). Year of publication explained 9.6% of the heterogeneity but was also not statistically significant (β = 0.104, 95% CI −0.079 to 0.287, *p* = 0.222). Smoking proportion and risk of bias explained 5.8% and 3.7% of the heterogeneity, respectively, without statistically significant associations. Diagnostic modality showed a relatively large, estimated effect (β = 2.583, 95% CI −0.224 to 5.390), with R^2^ = 31.7%, but the association was borderline and did not reach statistical significance (*p* = 0.066). The categorical analyses for region, study design, and risk of bias also showed no statistically significant individual contrasts (all *p* > 0.05). The parsimonious model retained year of publication as the only moderator. However, year was not statistically significant (β = 0.104, 95% CI −0.079 to 0.287, *p* = 0.222), explained only 9.6% of the observed heterogeneity, and substantial residual heterogeneity remained (τ^2^ = 2.850; I^2^ = 99.0%) (Supplementary Table S7, Supplementary Figure S7I).

## 4. Discussion

T2DM is a growing public health issue in Saudi Arabia, driven by urbanization, inactivity, and poor diets [49,50], with a great impact on quality of life and wellbeing [51]. It leads to serious microvascular and macrovascular complications, which reduce quality of life and increase mortality [24]. Various national and regional studies link these complications to poor glycemic control, hypertension, dyslipidemia, and longer diabetes duration [28,41]. However, inconsistent data and regional disparities hinder a unified understanding. This systematic review and meta-analysis aimed to synthesize prevalence and risk factors to guide policy and prevention strategies.

This systematic review included 37 eligible reports, of which 36 unique, non-overlapping datasets contributed to the quantitative synthesis. The pooled prevalences were 24% for retinopathy, 16% for nephropathy, 24% for neuropathy, 3% for cerebrovascular stroke, 5% for PAD, and 10% for diabetic foot disease. MI and CHD/CAD were also analyzed. Confirmed MI had a pooled prevalence of 10% across 14 studies, whereas two studies using broader CHD/CAD definitions yielded 20%; the combined estimate of 10% (16 studies) is presented as a secondary analysis. Individual studies frequently report associations between complications and poor glycemic control, longer diabetes duration, hypertension, obesity, dyslipidemia, smoking, and physical inactivity. In study-level meta-regression, statistically significant univariate associations were limited to diabetes duration and female proportion for nephropathy, smoking prevalence for neuropathy, and female proportion for PAD. Region explained a large share of the heterogeneity in MI/CHD/CAD prevalence. These aggregate associations are exploratory and do not establish patient-level causation. Extreme residual heterogeneity (I^2^ > 80% in all models) warrants cautious interpretation of all pooled estimates.

Regarding microvascular complications, this study showed a pooled retinopathy prevalence of 24.0%, which is lower than the global figure of 34.6% reported by Yau et al. [52]. However, it falls within the range reported across Middle east North Africa (MENA) populations, from 19% in the United Arab Emirates to 64% in Jordan [33] highlighting substantial regional variation in the burden of diabetic retinopathy. None of the factors examined showed a significant positive association with diabetic retinopathy prevalence. However, original studies have reported an increasing burden of hypertension in the region [53] and given the established evidence linking hypertension to diabetic retinopathy, its potential contribution to the regional burden of diabetic retinopathy warrants further investigation [11]. Nevertheless, individual Saudi datasets, such as Abu El-Asrar [38], showed the global trend, demonstrating a two-fold increased risk of retinopathy with hypertension, while Al-Rubeaan (2015) [16] linked poor lipid profiles to proliferative diabetic retinopathy. Similarly, Wondmeneh et al. [54] showed that diabetic patients with hypertension had an approximately 2.5-fold increased risk of developing diabetic retinopathy compared with those without hypertension (adjusted odds ratio (AOR) = 2.54, 95% CI 1.45–3.63). in the current review, the absence of significant associations between HbA1C and diabetic retinopathy in our meta-regression also contrasts with the United Kingdom Prospective Diabetes Study (UKPDS 35), which reported that each 1% increase in HbA1c was associated with a 21% increased risk of microvascular complications, including diabetic retinopathy [55]. Furthermore, the conducted meta-regression did not find a significant association between dyslipidemia and diabetic retinopathy prevalence. On the other hand, Liu et al. [56] reported that elevated lipid levels or dyslipidemia were risk factors for diabetic retinopathy. This discrepancy may reflect the predominance of cross-sectional study designs, differences in study populations and measurement methods, and the very high between-study heterogeneity, which may have limited the ability of the meta-regression to detect consistent study-level associations.

In this study, the pooled prevalence of diabetic nephropathy was 16.0%, slightly lower than the 23.7% reported by Al-Zahrani et al. [57]. In the meta-regression, hypertension showed a positive but non-significant association with the prevalence of diabetic nephropathy Supplementary Table S2, indicating that differences in study-level hypertension prevalence did not significantly explain the between-study variation in diabetic nephropathy prevalence. Nevertheless, evidence from individual studies indicates that effective blood pressure control can reduce the progression of microalbuminuria [6]. At the study level, female proportion and diabetes duration were significantly associated with nephropathy estimates in univariate meta-regression; these findings should not be interpreted as individual-level effects. Similarly, Zhang et al.’s [58] study found women had significantly higher diabetic nephropathy incidence than men (hazard ratio (HR) = 1.33 [95% CI 1.02–1.73]) and greater renal function decline (β = −7.346 [−8.906, −5.786]). Key reasons include loss of estrogen’s kidney-protective effect (73.5% were postmenopausal; only 1.8% used estrogen), higher dyslipidemia and obesity rates, lower medication adherence, and less intensive treatment compared to men. Like our findings, Pantis et al. [59] found that longer disease duration was associated with progressively lower GFR and substantially higher odds of chronic kidney disease, even after adjustment for age, hypertension, and other metabolic factors. On the same line, Tziomalos et al. [60] reported that longer diabetes duration contributes to the development and progression of diabetic nephropathy. This association may reflect the cumulative effects of prolonged metabolic stress, which can contribute to progressive structural and functional renal damage over time [61].

Moreover, neuropathy showed a high pooled burden (24.0%) with a wide prediction interval of 2 to 82%. This prevalence is lower than the global pooled prevalence of 46.7% for painful diabetic neuropathy reported by Tao et al. [62], which may reflect gaps in Saudi screening practices, with many studies relying on symptom enquiry or single-point vibration tests rather than nerve conduction studies [34]. Smoking prevalence showed a statistically significant univariate study-level association with neuropathy estimates; this does not establish smoking as an individual-level causal determinant. In the same vein, a meta-analysis on seventy-five articles involving 228,699 individuals with diabetes showed an increased risk of diabetic peripheral neuropathy among smokers (relative risk (RR) 1.28; 95% CI 1.05–1.51) [63]. This finding is consistent with the literature, which has linked neuropathy to chronic hyperglycemia, obesity, and smoking [44].

In this study, macrovascular events were less frequently reported in the included studies, yet they were associated with the most serious clinical outcomes. Stroke prevalence (3.0%) falls within the international range of 2–15% [64,65], and this finding is particularly concerning given that patients with DM have a 1.5–3-fold higher stroke risk compared with normoglycemic populations, as reported by Erkins et al. [66]. The stroke meta-regression found that HbA1c was the largest moderator (R^2^ = 24.5%, *p* = 0.167), then region (21.3%) and study design (14.6%), with no other significant moderator. Although hypertension was not a significant moderator of stroke prevalence in our meta-regression, Davis et al. [67] demonstrated that hypertension and systolic blood pressure were independently associated with stroke after adjustment for age and sex. These findings suggest that the absence of significant moderation in our analysis should not be interpreted as evidence that hypertension is unrelated to stroke risk, as study-level meta-regression may not capture individual-level associations.

In the current review, the pooled prevalence of MI and CHD/CAD was 10.0% (95% CI, 6.0–15.0%) across 16 studies, with substantial heterogeneity (I^2^ = 93.5%; τ^2^ = 0.96). The pooled prevalence of confirmed MI was 9% across 14 studies, whereas two studies using broader CHD/CAD definitions reported a prevalence of 20%. Accordingly, the combined estimate of 10% should be interpreted as a secondary analysis, with the distinction between confirmed MI and broader CHD/CAD definitions considered critical to its interpretation. These findings are broadly consistent with a large-scale systematic review and meta-analysis by Einarson et al. 2018 [68], which synthesized data from 57 articles comprising 4,549,481 persons with T2DM. Authors found that 32.2% of the population had cardiovascular disease, with CAD at 21.2%, heart failure at 14.9%, angina at 14.6%, MI at 10.0%, and stroke at 7.6%. In the present review, region accounted for 84.5% of the between-study heterogeneity in MI/CHD/CAD prevalence (τ^2^ = 0.110; I^2^ = 87.1%). Significant regional differences were observed for Tabuk (β = −3.325, *p* = 0.0004) and Taif (β = −4.587, *p* = 0.0023) relative to the reference region. This substantial geographical variation may reflect differences in healthcare access, diagnostic capacity, referral patterns, and underlying population risk profiles. These findings suggest that a single national pooled estimate may obscure clinically relevant regional disparities in MI/CHD/CAD prevalence [69].

Notably, our primary pooled PAD prevalence was 5.0% (the outlier-adjusted sensitivity estimate was 4.0%), which is lower than what was reported by Arif et al. [70] and the 9–55% range reported in hospital-based international studies by Soyoye et al. [71]. The lower prevalence may partly reflect underdiagnosis, as most included studies relied primarily on ABI thresholds without confirmatory duplex ultrasonography, potentially leading to underestimation of the true PAD burden. Female proportion was a significant moderator of PAD prevalence in our meta-regression. This finding is consistent with evidence that PAD may differ by sex in its risk factors, clinical presentation, and detection. Women with PAD are more likely to present with atypical leg symptoms and may have greater functional limitations, potentially contributing to delayed diagnosis or differences in reported prevalence. In addition, certain risk factors for PAD, like smoking, may have a stronger association with PAD among women [72,73]. Therefore, the significant female-sex effect observed in our meta-regression may reflect differences in risk-factor profiles, symptom presentation, detection, or study population characteristics rather than an independent causal effect of sex. Given that meta-regression uses study-level rather than individual-level data, this finding should be interpreted cautiously.

Diabetic foot complications pooled at 10.0%. However, this estimate must be interpreted within the methodological constraints of the available evidence. The predominance of cross-sectional designs precludes temporal sequencing and therefore limits causal inference, while the extreme between-study heterogeneity (I^2^ = 98.3%) undermines the identification of consistent moderator–outcome relationships. These findings are consistent with previous evidence showing substantial variation in diabetic foot ulcer prevalence across populations. Zhang et al. [74] reported a global pooled prevalence of 6.3%, with extreme heterogeneity (I^2^ > 95%) and wide prediction intervals, which they attributed to differences in case ascertainment, screening methods, and population risk profiles. In the present review, comparability was further limited by differences in diagnostic criteria, clinical settings, and definitions of study-level covariates. Several meta-regression models also included relatively few studies or had substantial missing moderator data, reducing statistical power and increasing the possibility of false-negative findings. Therefore, non-significant coefficients should be interpreted as a lack of detectable study-level association rather than evidence that the corresponding clinical risk factors are unimportant.

In synthesizing the factors associated with vascular complications in T2DM, several key determinants emerged. Although our meta-regression models did not identify statistically significant associations for most study-level variables, evidence from the broader literature and national data consistently supports the central role of poor glycemic control and longer diabetes duration in the development and progression of vascular complications [23]. Saudi studies with mean HbA1c levels exceeding 9%, such as Al Toijry [41], consistently reported higher complication rates, supporting established mechanisms involving oxidative stress and endothelial dysfunction, as described by Liu et al. [75]. Obesity was also highly prevalent, and its association with vascular complications is consistent with global evidence implicating insulin resistance and chronic inflammation as underlying mechanisms. Individual studies reported associations of smoking and physical inactivity with some cardiovascular and neuropathic outcomes, whereas the study-level meta-regressions generally did not demonstrate consistent associations, consistent with what was reported by Tegegne et al. [76].

*Strengths and Limitations*

This systematic review and meta-analysis has several strengths, including its large aggregate sample, Saudi-specific focus, and evaluation of both microvascular and macrovascular complications. Important limitations remain. Substantial between-study heterogeneity was present across most outcomes, reflecting differences in study populations, clinical settings, designs, and diagnostic definitions. Several pooled estimates therefore represent an average across clinically dissimilar settings and have wide prediction intervals. Funnel-plot asymmetry, where present, may indicate small-study effects, selective reporting, or heterogeneity; it should not be described as proof of publication bias. Meta-regression used aggregate study-level covariates that may not reflect the distribution of variables among affected individuals, can differ in direction or magnitude from patient-level relationships, and cannot adequately control individual-level confounding or effect modification. Consequently, all moderator findings are ecological, exploratory, and non-causal. In addition, some subgroup and sensitivity analyses contained few studies, limiting precision and the reliability of subgroup comparisons. Geographical representation was also uneven, with a large proportion of studies conducted in Riyadh and limited evidence from several other Saudi regions. This concentration may reduce the generalizability of the pooled estimates to the broader Saudi population, particularly where healthcare access, referral patterns, and diagnostic practices differ by region.

*Clinical and Research Implications*

National diabetes programs in Saudi Arabia should incorporate standardized annual retinal photography, urine albumin screening, neuropathy assessment, and ABI measurement, together with structured lifestyle counseling to facilitate the early detection and prevention of vascular complications. Future research should prioritize prospective longitudinal Saudi cohorts to better define temporal relationships between risk factors and complications, standardize diagnostic criteria and assessment methods, evaluate interventions such as structured exercise through randomized controlled trials, and leverage electronic health record databases to monitor long-term clinical outcomes in routine practice.

## 5. Conclusions

This systematic review and meta-analysis demonstrated a substantial burden of diabetes-related microvascular and macrovascular complications among adults with T2DM in Saudi Arabia. The pooled prevalences were 24% for retinopathy, 16% for nephropathy, 24% for neuropathy, 3% for cerebrovascular stroke, 5% for PAD, and 10% for diabetic foot disease, while the combined MI/CHD/CAD prevalence was 10% (confirmed MI 9%; broader CHD/CAD 20%). However, substantial variation in study populations, clinical settings, outcome definitions, and diagnostic approaches limits the generalizability and clinical interpretation of these pooled estimates. Confirmed MI should be distinguished from broader CHD/CAD outcomes, while diabetic foot disease should be considered separately from conventional macrovascular complications. Across individual studies, poor glycemic control, longer diabetes duration, hypertension, dyslipidemia, obesity, smoking, and physical inactivity were frequently reported to be associated with complications. However, study-level meta-regression findings were limited and exploratory and should not be interpreted as patient-level causal associations. Marked regional variation was also observed, highlighting the potential importance of geographical differences in healthcare access, diagnostic practices, referral patterns, and population risk profiles. These findings highlight the need for standardized case definitions, harmonized screening and diagnostic protocols, prospective population-based studies, and transparent reporting of setting-specific estimates. Saudi diabetes programs should expand structured, guideline-based screening for retinopathy, nephropathy, neuropathy, and PAD, alongside integrated lifestyle counseling and systematic management of modifiable cardiovascular risk factors. Future research should prioritize representative population-based designs and consistent outcome definitions to improve the comparability of estimates and clarify the geographical distribution of diabetes-related complications across Saudi Arabia. Study-level moderator findings should remain explicitly framed as exploratory and should not be interpreted as patient-level causal risk estimates.

## Figures and Tables

**Table 1:** Study characteristics, design, and comorbidities of included studies.

Author, Year, Region	Study Design	Study Setting	Age Mean ± SD	Female %	Participants (n)	Comorbidities
**Abdulghani, 2018, Riyadh (Riyadh Region) [27]**	Cross-sectional	Primary Health Care centers	54.38 ± 12.2	62.8%	360	Hypertension: 61.4%|Dyslipidemia: 58.1%|Smoking: 13.3%|CVD: 14.4%
**Abu El-Asrar, 1998, Riyadh (Riyadh Region) [38]**	Cross-sectional	Diabetes Center, King Abdulaziz University Hospital	48.6 ± 14.2	50.0%	502	Hypertension: 24.1%
**Ahmed, 2014, Jeddah (Makkah Region) [21]**	Cross-sectional	Primary Health Care centers	51 ± 11	42.9%	350	Hypertension: 56.0%|Smoking: 18.0%|Phys. inactivity: 63.1%
**Ahmed, 2016, Abha (Asir Region) [7]**	Cross-sectional	Diabetes center	54.6 ± 0.3	42.4%	401	BMI 30.82 ± 6.2 Hypertension: 36.4%
**Alabdulwahhab, 2019, Al-Majmaah (Riyadh Region) [26]**	Cross-sectional	Primary Health Care centers registry, Al-Majmaah	54.95 ± 11.65	38.6%	327	
**Alaboud, 2016, Riyadh (Riyadh Region) [37]**	Cross-sectional	Al-Wazarat Healthcare Center	57.9 ± 11.6	57.6%	748	BMI 18.5–24.9: 9.6%|25–29.9: 26.1%|≥30: 64.3% Hypertension: 28.9%|Dyslipidemia: 38.0%| CVD: 54.0%
**Al-Ayed, 2019, Riyadh (Riyadh Region) [12]**	Cross-sectional	Diabetic foot clinic	56.4 ± 6.72	34.6%	81	BMI 18.5–24.9: 12.3%|25–29.9: 38.3%|≥30: 49.4% Hypertension: 77.8%
**Al-Esawi, 2021, Riyadh (Riyadh Region) [30]**	Cross-sectional	Primary Health Care centers (PHC)	61.3 ± 10.9	0.0%	351	BMI 18.5–24.9: 17.4%|25–29.9: 43.6%|≥30: 39.0% Hypertension: 65.2%|Dyslipidemia: 67.5%| Smoking: 30.8%|Phys. inactivity: 74.1%
**Alfadda, 2006, Riyadh (Riyadh Region) [47]**	Retrospective cohort	Primary care clinics, King Khalid University Hospital	56.6 ± 12	46.5%	99	BMI mean = 30.8 ± 5.8
**Algeffari, 2018, Riyadh (Riyadh Region) [31]**	Cross-sectional	Primary Health Care clinics	56.9 ± 8.3	60.7%	242	BMI 18.5–24.9: 46.3%|25–29.9: 35.5%|≥30: 17.8% Hypertension: 7.9%|Dyslipidemia: 38.0%| Smoking: 5.0%|
**AlGhamdi, 2020, Jeddah (Makkah Region) [29]**	Cross-sectional	Primary care clinics	64.5 ± 15	38.8%	116	|CVD: 49.1%
**Alghamdi, 2021, Riyadh (Riyadh Region) [33]**	Cross-sectional	Primary care center	61 ± 11	65.0%	428	BMI 18.5–24.9: 17.3%|25–29.9: 24.5%|≥30: 66.4% Smoking: 3.0%
**Alguwaihes, 2023, Multiple regions (Riyadh Region; Makkah Region [Jeddah, Taif]; Eastern Region [Al Ahsa]; Al Qassim Region [Buraidah])** **[35]**	Cross-sectional	Saudi sites from the CAPTURE study	58 (50–65)	45.8%	883	BMI 29.1 (26.5–32.9) Hypertension: 55.5%|Dyslipidemia: 20.3%|Smoking: 8.7%|Phys. inactivity: 17.9%
**Alharajin, 2023, Al Ahsa (Eastern Region) [28]**	Cross-sectional	National Guard Primary Health Care clinics (Family Medicine Centers at King Abdulaziz Hospital & Iskan)	48.5 ± 12.3	52.6%	342	BMI 18.5–24.9: 20.2%|25–29.9: 46.2%|≥30: 33.6% Hypertension: 7.0%|Dyslipidemia: 20.2% Smoking: 21.9%
**Aljarid, 2018, Domat Aljandal & Quraiat (Al-Jouf Region) [25]**	Cross-sectional	Five Primary Health Care Centers	59.2 ± 10.4	23.5%	149	BMI mean 30.26 ± 6.62 Hypertension: 57.0%|Smoking: 47.0%
**Aljehani, 2020, Riyadh (Riyadh Region) [23]**	Cross-sectional	King Khalid University Hospital	50.6 ± 11.2	62.7%	469	BMI 18.5–24.9: N/A|25–29.9: N/A|≥30: 71.8% Hypertension: 76.3%|Dyslipidemia: 43.9% Smoking: 13.0%|Phys. inactivity: 9.6%|CVD: 9.6%
**Alnaim, 2023, Al Ahsa & Dammam (Eastern Region) [32]**	Cross-sectional	Diabetes clinics, Primary Health Care Diabetes Mellitus Clinics at King Abdulaziz Hospital and Imam Abdulrahman bin Faisal Hospital, National Guard Health Affairs	58.69 ± 9.46	54.1%	935	BMI 18.5–24.9: 6.1%|25–29.9: 24.1%|≥30: 69.8% Hypertension: 71.9%|Dyslipidemia: 86.3%
**Alramadan, 2019, Hofuf (Eastern Region); Riyadh (Riyadh Region); Jeddah (Makkah Region) [13]**	Cross-sectional	Diabetes centers	57.6 ± 11.1	65.2%	1111	BMI mean = 32.9 (±8.1) ≥30: 19.5%
**Al-Rubeaan, 2014, N/A [6]**	Cross-sectional	Saudi National Diabetes Registry (SNDR)	59.9 ± 12.7	44.5%	54,670	BMI 18.5–24.9: 18.0%|25–29.9: 33.8%|≥30: 48.2% Hypertension: 51.3%|Dyslipidemia: 38.0% Smoking: 8.0%
**Al-Rubeaan, 2015, Riyadh (Riyadh Region) [16]**	Cross-sectional	Not specified (Saudi cohort)	59.7 ± 12.78	44.0%	50,464	BMI 18.5–24.9: 18.0%|25–29.9: 33.8%|≥30: 48.1% Hypertension: 51.2%|Dyslipidemia: 39.1%|Smoking: 6.5%
**AlSenany, 2015, Jeddah (Makkah Region) [24]**	Cross-sectional	Ministry of Health primary care diabetes clinics (four centers)	58 ± N/A	57.7%	234	BMI 18.5–24.9: N/A|25–29.9: N/A|≥30: N/A Hypertension: 38.0%|
**Alshammari, 2022, Riyadh (Riyadh Region) [39]**	Cross-sectional	Not specified	55.9 ± 6.7	54.0%	430	BMI 18.5–24.9: 8.6%|25–29.9: 30.7%|≥30: 60.5% Hypertension: 37.4%|Smoking: 4.4%
**Alshaya, 2024, Riyadh (Riyadh Region) [40]**	Cross-sectional	King Abdulaziz Medical City and King Abdullah Bin Abdulaziz University Hospital	59.3 ± 13.1	57.0%	1218	BMI mean & SD = 31.9 ± 6.5 BMI > 30% = 701 (58.3%) Hypertension: 67.7%|Dyslipidemia: 69.1%|Smoking: 7.6%|CVD: 18.6%
**AlSidr, 2019, Riyadh (Riyadh Region) [22]**	Retrospective Cohort	Emergency department	59.7 ± 6.4	42.3%	26	Hypertension: 76.9%|Dyslipidemia: 42.3%
**Altoijry, 2021, Riyadh (Riyadh Region) [41]**	Cross-sectional	Not specified (diabetic foot population)	62.03 ± 0.3	38.1%	404	
**Alwakeel, 2008, Riyadh (Riyadh Region) [20]**	Retrospective cohort study	Adult Saudi patients with type 2 diabetes (clinic or hospital-based)	58.4 ± 14.2	51.7%	1952	BMI 18.5–24.9: 15.7%|25–29.9: 39.0%|≥30: 44.8% Hypertension: 78.1%|Dyslipidemia: 39.1%
**Al-Zamil, 2017, Al-Khobar (Eastern Region) [11]**	Cross-sectional	King Fahad Hospital of the University,	51.2 ± 5.3	55.4%	112	BMI ≥ 30: 38.1% Hypertension: 27.7%|Dyslipidemia: 58.0%|Smoking: 17.0%
**Assiri, 2022, West region (Taif City) [48]**	Retrospective cohort study	Patients with type 2 diabetes attending Prince Mansour Military Hospital	62.0 ± 12.2	56.4%	349	BMI 18.5–24.9: NA|25–29.9: NA|≥30: NA Hypertension: 53.5%|Dyslipidemia: 69.7%
**Bajaber, 2021, Makkah Region [14]**	Cross-sectional	Al-Noor Specialist Hospital	56.8 ± 9.9	61.8%	251	BMI = 28.51 ± 5.78 Hypertension: 63.7%|Dyslipidemia: 43.4%
**Bokhary, 2019, Jeddah (Makkah Region) [45]**	Case–control	Not specified (diabetic foot subgroup comparison)	62 ± 14.05	38.1%	402	BMI 18.5–24.9: 14.2%|25–29.9: 10.9%|≥30: 13.4% Hypertension: 68.9% Smoking 17.7%
**Ewid, 2023, Al Qassim Region [36]**	Cross-sectional	Diabetes Center, King Fahad Specialist Hospital	57.5 (49–61)	38.0%	200	BMI 29.93 (26 3- 35.0) Hypertension: N/A|Dyslipidemia: 81.0%| Smoking: 14.0%
**Ghabban, 2020, Tabuk (Tabuk Region) [42]**	Cross-sectional	Chronic illness clinics, King Khaled Hospital	58.2 ± 11.6	36.3%	697	BMI 18.5–24.9: 6.2%|25–29.9: 24.1%|≥30: 69.7% Smoking: 6.0%|Phys. inactivity: N/A|CVD: N/A
**Jatooi, 2021, Al-Khobar (Eastern Region) [43]**	Cross-sectional	Internal medicine clinics	56.4 ± 11.2	62.6%	187	BMI 18.5–24.9: 9.1%|25–29.9: 24.6%|≥30: 66.3%
**Nielsen, 1998, Riyadh (Riyadh Region) [34]**	Cross-sectional	Not specified (Saudi national cohort)	56 ± 10.4	21.6%	375	BMI 18.5–24.9: N/A|25–29.9: N/A|≥30: N/A Hypertension: 9.3%|Smoking: 4.0%
**Saiyed, 2022, Tabuk [46]**	Retrospective cohort study	Adult T2DM patients attending two military hospitals in Tabuk	51.3 ± 12.8 years	36.5%	1563	BMI median: 30.0 (27.0–36.0)
**Wang, 2014, N/A [44]**	Cross-sectional	Jeddah Newspaper invitation	53.4 ± 10.5	37.3%	552	Hypertension: 48.9%|Dyslipidemia: 50.7%| Smoking: 16.3%|Phys. inactivity: N/A

Abbreviations: N/A: category not applicable/assessed; BMI, body mass index; CAPTURE, Cardiovascular and Diabetes Outcomes in the CAPTURE study; CVD, cardiovascular disease; PHC, primary health care; SD, standard deviation; SNDR, Saudi National Diabetes Registry; T2DM, type 2 diabetes mellitus. Retrospective case–control chart review comparing 199 patients with T2DM and diabetic foot ulcers with 200 patients with T2DM without diabetic foot ulcers to assess diabetic retinopathy and associated factors. Sample size in abstract is 399 while in the main text 401, Nb: Al-Rubeaan (2014) [6] and Al-Rubeaan (2015) [16] are 2 different studies by the same first author on the same data base with different objectives.

**Table 2:** Characteristics of included studies: diabetes duration, diagnostic criteria, and glycemic control.

Study (Author, Year)	Diabetes Duration (Years)	Diagnostic Criteria (Complications)	HbA1c, Mean (SD)/Range	Glycemic Control Status		
				Controlled (≤7%)	Uncontrolled (7–9%)	Highly Uncontrolled (>9%)
**Abdulghani, 2018 [27]**	>10 years in 62.8%	Hospital records and patient interviews	NA	41.4%	26.1%	32.5%
**Abu El-Asrar, 1999 [38]**	9.4 ± 6.6	Clinical exam by retinal specialist	NA	N/A	N/A	N/A
**Ahmed, 2014 [21]**	13 ± 7; 11 ± 8	Clinical examination; monofilament test; vascular palpation	7.5%: 66%	NA	NA	NA
**Ahmed, 2016 [7]**	13.4 ± 7.9	Retinopathy: slit lamp + fundus photography. Nephropathy: microalbuminuria or elevated creatinine. Neuropathy: clinical diagnosis	9.26 (2.3)	7 pts with DR	53 pts with DR	86 pts with DR
**Alabdulwahhab, 2019 [26]**	N/A	Ophthalmic examination	9.32 ± 1.07	N/A	N/A	N/A
**Alaboud, 2016 [37]**	<10 y: 47.3%; 10–19 y: 36.5%; ≥20 y: 16.2%	Medical records; modified Toronto Clinical Neuropathy Score (mTCN) score for neuropathy	NA	21.1%	30.9%	35.3%
**Al-Ayed, 2019 [12]**	≥10 y in 85.2%	Neuropathy: monofilament + tuning fork. Vascular disease: palpation + symptoms. Foot ulcer: Wagner’s classification	NA	4.9%	NA	95.1%
**Al-Esawi, 2021 [30]**	N/A	Clinical records: HbA1c, BP, cholesterol; physician confirmation	NA	54.1%	N/A	N/A
**Alfadda, 2006 [47]**	11.8	Retinopathy: eye exam. Nephropathy: 24-h urine. Foot exam: protective sensation + vascular status. HbA1c	NA	24.7%	24.8%	50.5%
**Algeffari, 2018 [31]**	<5 y: 20.2%; 5–10 y: 47.6%; >10 y: 32.2%	Michigan Neuropathy Screening Instrument Score (MNSI) > 2	NA	9.5% (PDPN group)	32.2% (PDPN group)	58.3% (PDPN group)
**AlGhamdi, 2020 [29]**	N/A	DPN assessed using Neuropathy Disability Score (NDS ≥ 5)	8.4 ± 1.8	NA	NA	NA
**Alghamdi, 2021 [33]**	13	Fundus photography; International Clinical DR Severity Scale	NA	N/A	N/A	N/A
**Alguwaihes, 2023 [35]**	10 (5–17)	Medical records and patient self-report	NA	N/A	N/A	N/A
**Alharajin, 2023 [28]**	N/A	Douleur Neuropathique-4 (DN4) questionnaire (≥4)	NA	45.0%	33.0%	21.9%
**Aljarid, 2018 [25]**	9.13	ABI < 0.9	NA	N/A	N/A	N/A
**Aljehani, 2020 [23]**	6.2	Physician diagnosis + HbA1c, blood pressure, lipid profile, BMI	8.3 ± 2.5%	NA	NA	NA
**Alnaim, 2023 [32]**	>3	Retinopathy: annual eye exam. Nephropathy: GFR < 60 or albuminuria ≥ 30 mg/day for ≥3 months. Neuropathy: loss of pinprick, vibration, or temperature sensation	8.04 (2.83)	NA	NA	NA
**Alramadan, 2019 [13]**	13.9	Retinopathy: ophthalmologist confirmed. Neuropathy: MNSI ≥ 7. Nephropathy: eGFR ≤ 60 mL/min/1.73 m^2^. CAD: documented. Diabetic foot: ulcers/amputation. Stroke: documented CVA	NA	N/A	N/A	N/A
**Al-Rubeaan, 2014 [6]**	13.55 ± 8.14	American Diabetes Association criteria: microalbuminuria 30–299 mg/g; macroalbuminuria ≥ 300 mg/g; ESRD: GFR < 30 mL/min/1.73 m^2^	NA	N/A	60.6%	N/A
**Al-Rubeaan, 2015 [16]**	13.4 ± 8.24	NPDR: cotton wool spots, hemorrhages, hard exudates, microaneurysms. PDR: neovessels	8.9 (2.32)	NA	NA	NA
**AlSenany, 2015 [24]**	N/A	WHO fasting plasma glucose ≥ 7.0 mmol/L (T2DM); standard criteria for obesity and CHD	NA	N/A	N/A	N/A
**Al-Shammari, 2022 [39]**	17.33 ± 4.48	NDS, DNS scores; EMG/NCV	10 (1.82)	4.0%	N/A	92.0%
**Alshaya, 2024 [40]**	N/A	American College of Cardiology (ACC), WHO, Kidney Disease Improving Global Outcomes (KDIGO) criteria, and AHA/ACC/HFSA guidelines	8.1 ± 1.9%	NA	NA	NA
**AlSidr, 2019 [22]**	>10 y: 73.1%; ≤10 y: 26.9%	Clinical diagnosis from patient records	8.4 ± 0.43%	NA	NA	NA
**Altoijry, 2021 [41]**	N/A	Ankle-brachial index (ABI) < 0.9 or Toe-brachial index (TBI) < 0.7	9.81 ± 2.28%	NA	NA	NA
**Al-Wakeel, 2008 [20]**	10.41 ± 7.45 years	Retinopathy: retinal hemorrhages, exudates, macular edema	NA	NA	NA	NA
**Al-Zamil, 2017 [11]**	within 1 month	Slit-lamp biomicroscope; retinal imaging; WHO DR grading criteria	NA	N/A	N/A	55.0%
**Assiri, 2022 [48]**	NA	Nephropathy: persistent proteinuria (≥3 dipstick readings/year) or serum creatinine > 130 µmol/L	8.9 ± 2.1%	NA	NA	NA
**Bajaber, 2021 [14]**	11–16 y: 36.6%; >16 y: 13.6%	Fundus exam; ADA HbA1c targets	NA	44.2%	55.8%	N/A
**Bokhary, 2019 [45]**	16.7 ± 10.02	Fundoscopic exam; International Clinical DR Severity Scale	8.6 ± 2.47	NA	NA	NA
**Ewid, 2023 [36]**	114–156 months	ADA guidelines	NA	22.5%	N/A	NA
**Ghabban, 2020 [42]**	10.8 ± 7.3	ADA guidelines, 2020	8.4 (1.7)	18.5%	81.5%	N/A
**Jatooi, 2021 [43]**	N/A	MNSI questionnaire + physical exam	8.11 (1.63)	31.0%	63.1%	5.9%
**Nielsen, 1998 [34]**	8 (2–16)	Neuropathy: clinical examination; Hypertension: SBP ≥ 160 or DBP ≥ 95 mmHg or on antihypertensive therapy (WHO)	8.8 (8.4–9.2)	NA	NA	NA
**Saiyed, 2022 [46] **	8.3 ± 4.6 years	Neuropathy: numbness, paraesthesia, vibration loss, or absent reflexes	NA	NA	NA	NA
**Wang, 2014 [44]**	>10	Reduced vibration/touch sensation and neuropathic symptoms	9.0 (1.4)	NA	NA	NA

N/A = category not applicable/assessed; DPN: diabetic peripheral neuropathy; PDPN = painful diabetic peripheral neuropathy; PDR/NPDR = proliferative/non-proliferative diabetic retinopathy; MNSI = Michigan Neuropathy Screening Instrument, DA: American Diabetes Association.

## Data Availability

The original contributions presented in this study are included in the article and Supplementary Materials. Further inquiries can be directed to the corresponding authors.

## Supplementary Materials

The following supporting information can be downloaded at https://www.mdpi.com/article/10.3390/diagnostics16203433/s1: Supplementary Figures S1–S7 present outcome-specific subgroup, influence, leave-one-out, and meta-regression analyses for retinopathy (Figure S1: funnel plot 1X, subgroup analysis 1S, Baujat plot 1E, influence diagnostics 1F, leave-one-out effect sizes 1G, leave-one-out I^2^ 1H, meta-regression bubble plots 1I), nephropathy (Figure S2: 2X, 2S, 2E, 2F, 2G, 2H, 2I), neuropathy (Figure S3: 3X, 3S, 3E, 3F, 3G, 3H, 3I), cerebrovascular stroke (Figure S4: 4X, 4S, 4E, 4F, 4G, 4H, 4I), confirmed MI/broader CHD-CAD (Figure S5: 5X, 5S, 5E, 5F, 5G, 5H, 5I), peripheral arterial disease (Figure S6: 6X, 6S, 6E, 6F, 6G, 6H, 6I), and diabetic foot disease (Figure S7: 7X, 7S, 7E, 7F, 7G, 7H, 7I); Supplementary Tables S1–S7 present outcome-specific univariate and parsimonious meta-regression results—including model-level study counts, coefficients, confidence intervals, *p*-values, R^2^, and residual heterogeneity where available—for diabetic retinopathy (Table S1), nephropathy (Table S2), diabetic neuropathy (Table S3), cerebrovascular accident (Table S4), coronary artery disease and myocardial infarction (Table S5), peripheral arterial disease (Table S6), and diabetic foot (Table S7); additionally, the database-specific search strategies, outcome-definition table, master study-design list, overlap decisions, and study-level event/denominator audit should be supplied as separate supplementary tables and numbered consistently in the final submission package.

## Abbreviations

Abbreviation	Full Term
ABI	Ankle-Brachial Index
ACC	American College of Cardiology
ADA	American Diabetes Association
AHA	American Heart Association
AOR	Adjusted Odds Ratio
BMI	Body Mass Index
CAD	Coronary Artery Disease
CHD	Coronary Heart Disease
CI	Confidence Interval
CVD	Cardiovascular Disease
DM	Diabetes Mellitus
eGFR	Estimated Glomerular Filtration Rate
HbA1c	Glycated Hemoglobin
HDL	High-Density Lipoprotein
HR	Hazard Ratio
JBI	Joanna Briggs Institute
LDL	Low-Density Lipoprotein
MI	Myocardial Infarction
MNSI	Michigan Neuropathy Screening Instrument
OR	Odds Ratio
PAD	Peripheral Arterial Disease
PHC	Primary Health Care
SD	Standard Deviation
SNDR	Saudi National Diabetes Registry
T2DM	Type 2 Diabetes Mellitus
