# Supplementary material for: Microvascular and Macrovascular Complications and Their Associated Risk Factors Among Patients with Type 2 Diabetes in Saudi Arabia: A Systematic Review and Meta-Analysis
Source: Diagnostics (Basel). 2026 Oct 21;16(20):3433. doi: 10.3390/diagnostics16203433 (PMC13703109; doi:10.3390/diagnostics16203433)
Supplement: Supplementary file 1 [file diagnostics-16-03433-s001.zip › diagnostics-4554767-supplementary.pdf]

Supplementary File

Contents

Diabetic Retinopathy 3

Publication bias of diabetic retinopathy prevalence [Figure S1 X] 3

Subgroup analysis of diabetic retinopathy **[Figure S1 S]** 4

Sensitivity analysis of diabetic retinopathy prevalence 6

Meta-regression analysis of diabetic retinopathy prevalence 9

Diabetic Nephropathy 12

Publication bias of diabetic nephropathy prevalence [Figure S2 X] 12

Subgroup analysis diabetic nephropathy [**Figure S2 S**] 13

Sensitivity analysis of diabetic nephropathy 15

Meta regression of diabetic nephropathy prevalence 20

Diabetic Neuropathy 24

Publication bias of diabetic retinopathy prevalence [Figure S3 X] 24

Subgroup analysis of diabetic neuropathy prevalence [**Figure S3 S**] 25

Sensitivity analysis of diabetic neuropathy prevalence 27

Meta-regression analysis of diabetic neuropathy prevalence 33

Cerebrovascular stroke 37

Publication bias of diabetic retinopathy prevalence [Figure S4 X] 37

Subgroup analysis Cerebrovascular stroke prevalence **[Figure S 4 S]** 38

Sensitivity analysis Cerebrovascular stroke prevalence 40

Meta-regression analysis cerebrovascular stroke prevalence 45

Myocardial infarction and coronary artery disease 48

Publication bias of myocardial infarction and coronary artery disease prevalence [Figure S3 X] 48

Subgroup analysis of myocardial infarction and coronary artery disease **[Figure S5 S]** 49
Sensitivity analysis of myocardial infarction and coronary artery disease 51
Meta-regression analysis of myocardial infarction and coronary artery disease 55
Peripheral arterial diseases 60
Publication bias of Peripheral arterial diseases prevalence [Figure S3 X] 60
Subgroup analysis of peripheral arterial diseases prevalence **[Figure S6 S]** 61
Sensitivity analysis of peripheral arterial diseases prevalence 62
Meta-regression analysis of peripheral arterial disease prevalence 69
Diabetic foot ulcer 73
Publication bias of diabetic foot ulcer prevalence [Figure S3 X] 73
Subgroup analysis of diabetic foot ulcer prevalence **[Figure S7 S]** 74
Meta-regression analysis of diabetic foot prevalence 80

Diabetic Retinopathy

Publication bias of diabetic retinopathy prevalence [Figure S1 X]

The funnel plot displays marked asymmetry around the overall effect estimate (vertical dashed line at approximately -1.15), indicating potential publication bias or significant small-study effects:

- **Asymmetry and Outliers:** A substantial number of studies lie outside the pseudo-95% confidence bounds (white triangular funnel area), reflecting high between-study heterogeneity. Extreme outliers are visible on both ends of the outcome scale (e.g., observed outcomes near -3.0 and above 1.0).
- **Distribution of Small Studies:** Studies with larger standard errors (lower half of the plot) are sparsely and unevenly distributed, with several points scattered far to the left and right rather than forming a symmetrical, inverted funnel shape around the mean.
- **Interpretation:** This pronounced funnel asymmetry suggests that the meta-analysis is subject to publication bias or methodological differences across studies (such as varying sample sizes, diagnostic methods, or study populations).

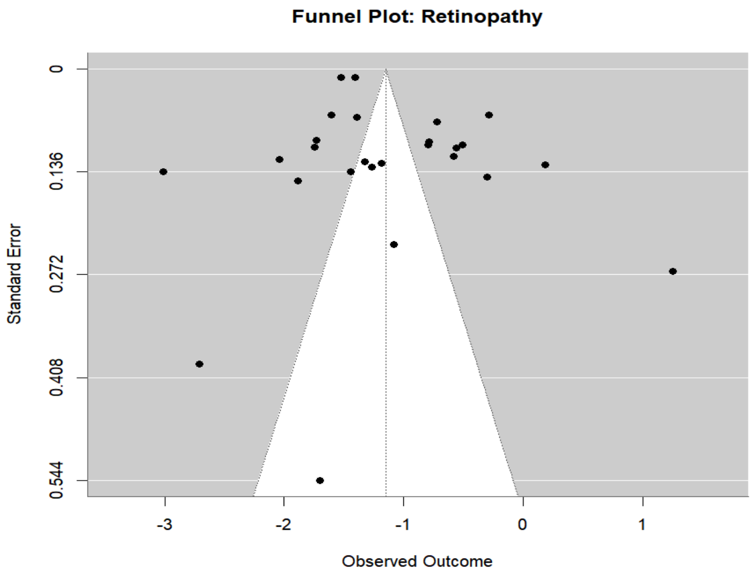

Figure S1 X: Funnel plot included studies assessing the pooled prevalence of retinopathy among patients with type 2 diabetes mellitus.

Subgroup analysis of diabetic retinopathy **[Figure S1 S]**

Subgroup analyses were conducted to evaluate the prevalence of diabetic retinopathy (DR) in Saudi Arabia based on risk of bias, study design, geographic region, and diagnostic tools:

- Risk of Bias (Supplementary Figure S1A): The pooled prevalence of DR was highest in studies with a moderate risk of bias at 30% (95%CI: 24%-36%), followed by low risk at 24% (95% CI: 17%-32%), and high risk at 22% (95% CI: 19%-26%). However, the test for subgroup differences showed no statistically significant variation (P=0.0843).
- Study Design (Supplementary Figure S1B): Pooled prevalence rates varied across study designs, with cross-sectional studies reporting 27% (95% CI: 20%- 35%), retrospective cohorts reporting 19% (95% CI: 16%-21%), prospective studies reporting 6% (95% CI: 3%- 12%), and case-control studies reporting 13% (95% CI: 10%- 17%). Differences across study designs were statistically significant (P<0.0001).
- Geographic Region (Supplementary Figure S1C): Prevalence varied markedly across geographic locations, ranging from 6% (95% CI: 3%- 12%) in Al-Khobar to 55% (95% CI: 48%- 61%) in Makkah. These geographic subgroup differences were statistically significant (P<0.0001).

- **Diagnostic Tool (Supplementary Figure S1 D):** The prevalence estimated via fundus photography/imaging was 26% (95% CI: 18%–35%), compared to 21% (95% CI: 15%–28%) using clinical examination or records. The test for subgroup differences between diagnostic methods was not statistically significant (P = 0.3718).

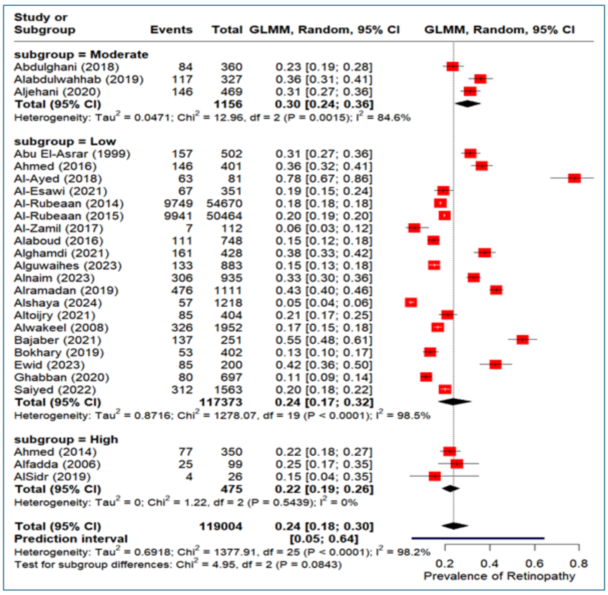

Supplementary figure A 1

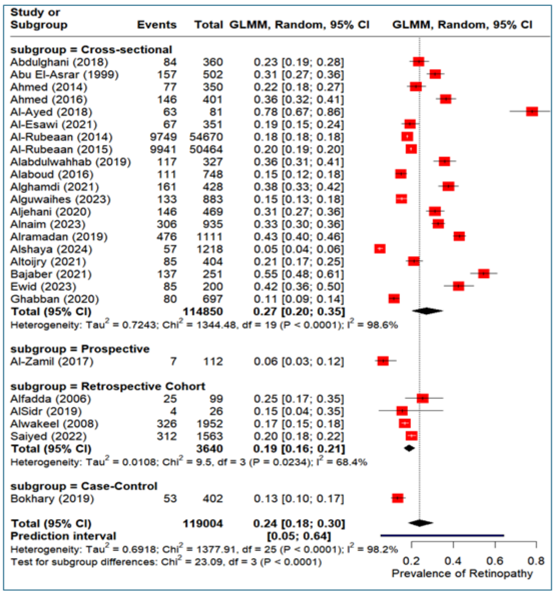

Supplementary figure B 1

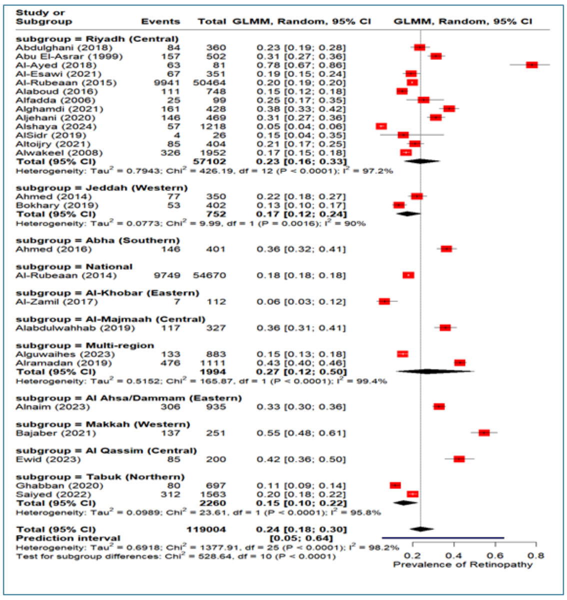

Supplementary figure 1 C

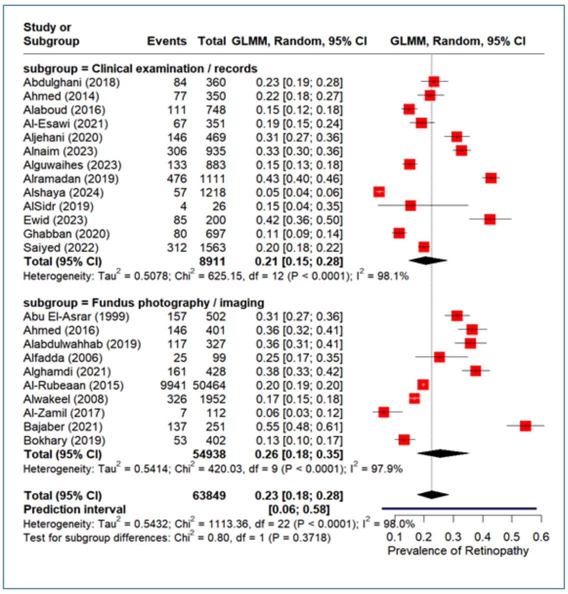

Supplementary figure 1 D

Figure S1 A-C: Subgroup analysis of diabetic retinopathy prevalence in Saudi Arabia by risk of bias, study design, geographic region, and diagnostic tool

Sensitivity analysis of diabetic retinopathy prevalence

The Baujat plot identifies Study 5 (Al-Ayed, 2018) as the primary source of overall heterogeneity, having both the highest squared Pearson residual (> 7.0) and a substantial influence on the overall pooled estimate (~0.34). Study 18 (Alshaya, 2024) also contributes significantly to heterogeneity (~4.8 squared Pearson residual) with a high overall influence (~0.24). Additionally, Study 9 (Al-Zamil, 2017) and Study 22 (Bajaber, 2021) demonstrate moderate influence and contribution to heterogeneity. The remaining studies cluster tightly near the origin (lower-left quadrant), indicating minimal contribution to trial heterogeneity and overall result stability. **Supplementary figure S1 E**

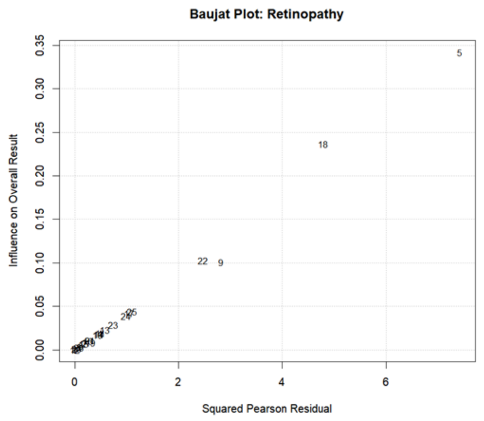

Figure S1 E: Baujat plot included studies assessing the pooled prevalence of retinopathy among patients with type 2 diabetes mellitus

The diagnostic plots identify Study 5 (Al-Ayed, 2018) and Study 18 (Alshaya, 2024) as influential outliers that significantly impact the overall meta-analysis results and heterogeneity:

- Residuals & Distance Metrics (rstudent, dffits, cook.d): Study 5 exhibits the highest positive studentized residual (> 3.0), highest DFFITS value (~0.58), and peak Cook's distance (~0.25), indicating it is an extreme outlier with strong leverage on the pooled effect. Study 18 exhibits a prominent negative studentized residual (~ -2.6) and a secondary peak in Cook's distance (~0.22).
- Heterogeneity Drop (tau2.del, QE.del): Removing Study 5 or Study 18 leads to the most substantial drops in total residual heterogeneity (τ^2) and Cochrane's Q test

statistic (QE), confirming both studies as the primary drivers of heterogeneity in the model.

- Leverage & Weight (hat, weight, cov.r): Across all studies, leverage (hat) remains relatively uniform (~0.04), while Study 5 and Study 18 marked noticeable drops in covariance ratios (cov.r), reflecting their disproportionate influence on the precision of the pooled variance. **Supplementary figure S1 F**

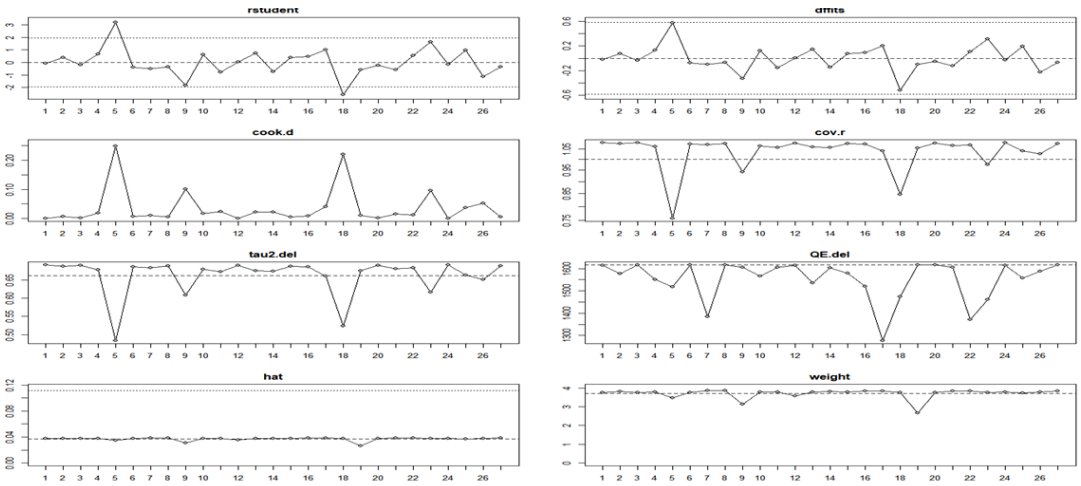

Figure S1 F: Influence analysis and diagnostic plots for meta-analysis of retinopathy prevalence in type 2 diabetes mellitus

The leave-one-out sensitivity plot demonstrates overall stability of the primary meta-analysis findings, as omitting any single study does not substantially shift the recalculated overall logit prevalence from the baseline reference line (dashed red line at approximately -1.15):

- **Main Influential Shifts:**
    - Omitting **Al-Ayed (2018)** causes the largest downward shift in overall logit prevalence (to approximately -1.24), confirming that its higher prevalence estimate was pulling the overall pooled result upward.
    - Omitting **Alshaya (2024)** causes the most noticeable upward shift in logit prevalence (to approximately -1.08), reflecting its influence on lowering the pooled effect.

- **Overall Robustness:** Despite the individual impact of these specific studies, all recalculations remain tightly centered around the baseline reference line, and their 95% confidence intervals continuously overlap across all iterations. This indicates that the final pooled prevalence estimate is highly robust and not overly dependent on any single study. **Supplementary figure S1 G**

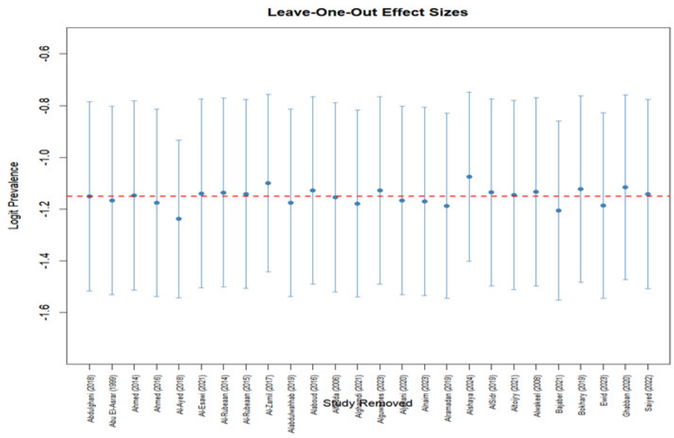

Figure S1 G: Leave-one-out sensitivity analysis of the pooled prevalence of retinopathy among patients with type 2 diabetes mellitus

The leave-one-out I^2 plot demonstrates that overall statistical heterogeneity remains exceptionally high ($I^2 > 99\%$) regardless of which individual study is omitted, though key studies exert a measurable influence on the overall variation:

- **Primary Contributors to Heterogeneity Reduction:**
  - Omitting Al-Rubeaan (2014) or Al-Rubeaan (2015) produces the most pronounced drop in heterogeneity, lowering I^2 from the baseline (dashed blue line at ~99.7%) to approximately 99.2%.
  - Omitting Al-Ayed (2018) or Alshaya (2024) also results in minor dips in heterogeneity (~99.6%).
- **Persistent Heterogeneity:** Despite these individual dips, I^2 remains consistently elevated above 99.0% across all iterations. This indicates that the observed heterogeneity is structural and systemic across the body of literature rather than driven by a single outlying study. **Supplementary figure S1 H**

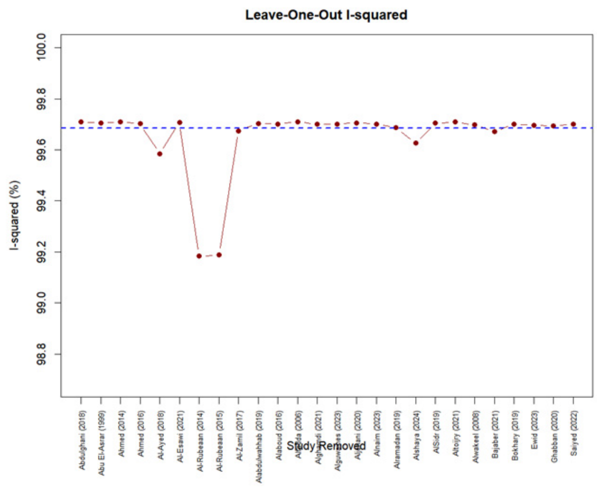

Figure S1 H: Leave-one-out sensitivity analysis of heterogeneity (I-squared) for the pooled prevalence of retinopathy among patients with type 2 diabetes mellitus

Meta-regression analysis of diabetic retinopathy prevalence

The meta-regression scatter plots illustrate the relationship between retinopathy prevalence (%) and various continuous study-level covariates, with circle sizes representing sample size weighting:

- **Positive Associations:**
  - **HbA1c (%):** Shows a distinct positive trend, indicating that higher mean HbA1c levels across study populations are associated with an increased prevalence of retinopathy.
  - **Dyslipidemia (%):** Exhibits a clear upward slope, demonstrating higher retinopathy rates in studies with greater proportions of dyslipidemia patients.
  - **Smoking (%) & Obesity (%):** Both demonstrate modest positive trends with prevalence.
- **Negative/Inverse Trends:**
  - **Mean Age (years) & DM Duration (years):** Show slight downward slopes relative to prevalence across the included study cohorts.
- **Neutral / Minimal Trends:**
  - **Publication Year, Hypertension (HTN %), & JBI Quality Score (%):** Display relatively flat regression lines (red dashed lines), indicating that prevalence estimates remained largely stable over publication years, varying hypertension rates, and study quality scores. **Supplementary figure S1 I**

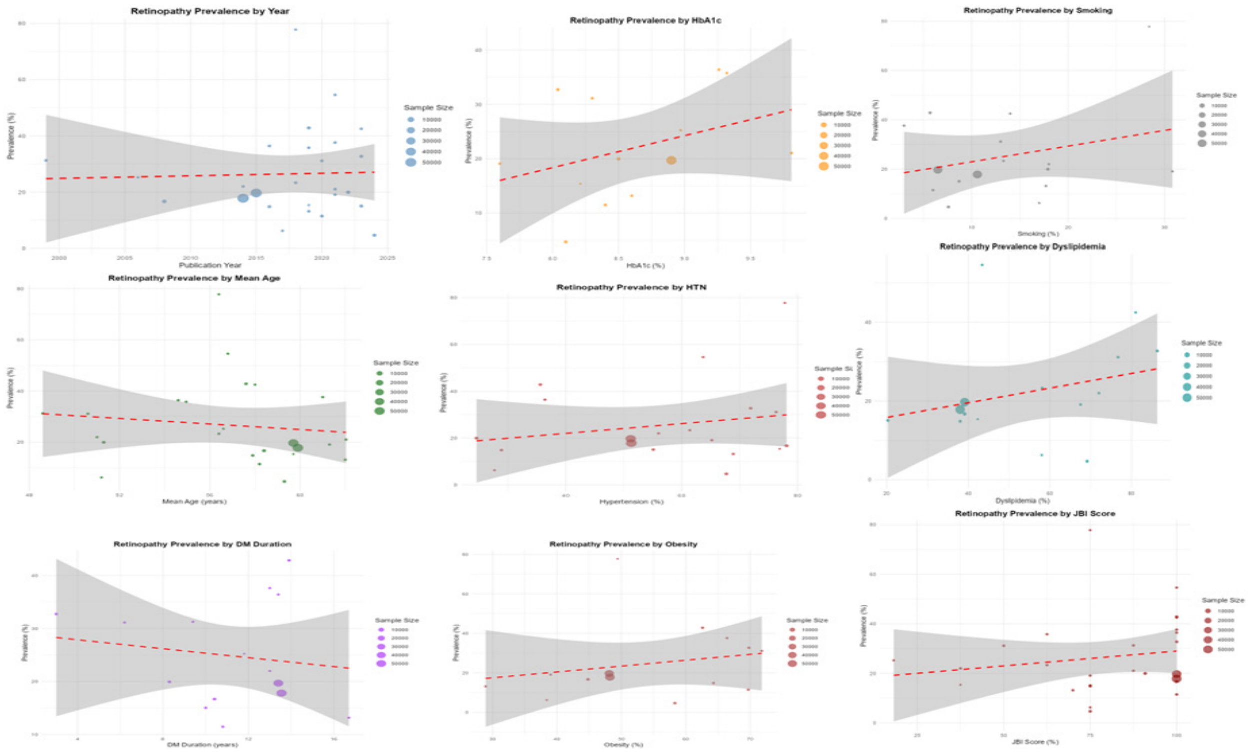

Figure S1 I: Meta-regression bubble plots examining the association between retinopathy prevalence and study-level moderators (publication year, HbA1c, smoking, mean age, hypertension, dyslipidemia, diabetes duration, obesity, and JBI Score)

The univariate meta-regression analyses showed no statistically significant associations between any of the evaluated study-level moderators and the prevalence of diabetic retinopathy. DM duration, diagnostic modality, dyslipidemia, female proportion, HbA1c, hypertension, JBI score, mean age, obesity, region, risk of bias, smoking, study design, and publication year all had p-values >0.05, and their 95% CIs crossed zero. HbA1c explained the greatest proportion of between-study heterogeneity (R^2 = 4.7%), while study design explained 6.1%, although neither association was statistically significant. Residual heterogeneity remained extremely high across all models, with residual I2 ranging from 98.4% to 99.9%, indicating that the examined study-level moderators explained little of the substantial variability in diabetic retinopathy estimates.

The model-fit results support this interpretation. The year-only model had R^2 = 0%, residual τ^2 = 0.7135, and residual I^2 = 99.7%. The residual heterogeneity test remained

statistically significant (QE = 1,386.53, $P < 0.0001$), indicating considerable unexplained heterogeneity after accounting for study year.

The absence of statistically significant meta-regression findings does not demonstrate that factors such as HbA1c, diabetes duration, age, or hypertension are unrelated to retinopathy at the individual-patient level. It means that, at the study level, the available data did not demonstrate that differences in these moderators explained the observed between-study heterogeneity. This distinction is particularly important because the residual heterogeneity was very high.

Table S1: Univariate meta-regression of study-level moderators for diabetic retinopathy

Study-level moderator	No. of studies	β coefficient	SE	95% CI	p-value	R^2 (%)	Residual I^2 (%)
**DM duration**	15	−0.0280	0.0429	−0.1207 to 0.0648	0.526	0.0	99.6
**Diagnostic modality**	23	0.2826	0.3254	−0.3942 to 0.9594	0.395	0.0	98.6
**Dyslipidemia (%)**	15	0.0088	0.0111	−0.0151 to 0.0327	0.442	0.0	99.8
**Female (%)**	26	0.0030	0.0128	−0.0234 to 0.0295	0.816	0.0	99.7
**HbA1c**	13	0.4090	0.3158	−0.2861 to 1.1040	0.222	4.7	98.4
**Hypertension (%)**	19	0.0101	0.0127	−0.0167 to 0.0368	0.438	0.0	99.8
**JBI score (%)**	26	0.0055	0.0077	−0.0103 to 0.0214	0.478	0.0	99.7
**Mean age**	24	−0.0328	0.0488	−0.1341 to 0.0685	0.509	0.0	99.7
**Obesity (%)**	14	0.0180	0.0217	−0.0293 to 0.0653	0.424	0.0	99.9
**Region**	26	−2.1504	1.3148	−4.9529 to 0.6521	0.123	0.0	99.1
**Risk of bias**	26	0.1480	0.5785	−1.0488 to 1.3447	0.800	0.0	99.7
**Smoking (%)**	16	0.0319	0.0329	−0.0386 to 0.1025	0.348	0.0	99.8
**Study design**	26	0.8864	0.8543	−0.8853 to 2.6581	0.311	6.1	99.7
**Year**	26	−0.0046	0.0306	−0.0679 to 0.0586	0.881	0.0	99.7

HbA1c = glycated haemoglobin; JBI = Joanna Briggs Institute; CI = confidence interval; SE = standard error; I^2 = residual heterogeneity; R^2 = proportion of between-study heterogeneity explained by the moderator; NS = not statistically significant. The reported residual I^2 values indicate very high unexplained heterogeneity across the models. The source analysis used a significance threshold of $P < .05$.

Diabetic Nephropathy

Publication bias of diabetic nephropathy prevalence [Figure S2 X]

The funnel plot exhibits clear asymmetry relative to the overall pooled effect line (vertical dashed line at approximately -1.6), indicating significant between-study heterogeneity and potential publication bias or small-study effects:

- **Asymmetry and Outlier Distribution:** Most of the included studies lie outside the pseudo-95% confidence bounds (white triangular area), reflecting substantial statistical heterogeneity. Studies are unevenly dispersed, with a cluster on the left side (lower prevalence outcomes, below -2.0) and several outlying studies scattered to the far right (observed outcomes extending above 1.0).
- **Small-Study Effects:** Studies with larger standard errors (lower portion of the plot) show considerable scatter away from the central pooled estimate rather than forming a symmetrical, inverted funnel shape.
- **Interpretation:** This visual asymmetry suggests that the pooled prevalence estimate for diabetic nephropathy may be influenced by reporting bias, differences in sample size, or clinical heterogeneity across studies (e.g., varying diagnostic criteria for nephropathy, study designs, or patient populations).

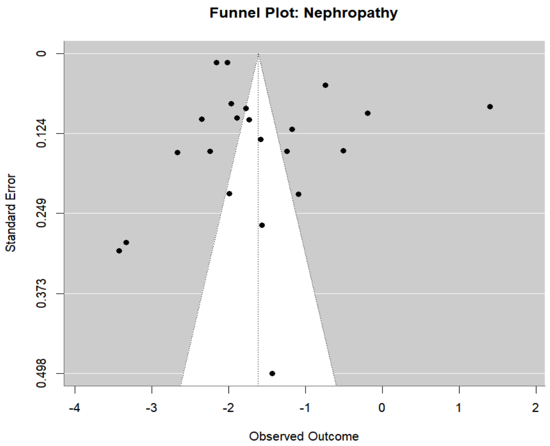

Figure S2 X: Funnel plot of included studies assessing the pooled prevalence of diabetic nephropathy among patients with type 2 diabetes mellitus.

Subgroup analysis diabetic nephropathy [**Figure S2 S**]

Subgroup analyses were conducted to evaluate the prevalence of diabetic nephropathy among Saudis based on risk of bias, study design, geographic region, and diagnostic tool:

- **Risk of Bias (Supplementary Figure S2 A):** The pooled prevalence of diabetic nephropathy was highest in studies with a moderate risk of bias at 45% (95% CI: 41%–50%), compared to low risk at 16% (95% CI: 11%–24%) and high risk at 10% (95% CI: 4%–23%). These subgroup differences were statistically significant (χ^2 = 44.15, df = 2, P < 0.0001).
- **Study Design (Supplementary Figure S2 B):** Pooled prevalence estimates varied across study designs, with prospective studies reporting 25% (95% CI: 17%–34%), cross-sectional studies reporting 17% (95% CI: 11%–26%), and retrospective cohort studies reporting 14% (95% CI: 7%–26%). The test for subgroup differences by study design was not statistically significant (χ^2 = 3.59, df = 2, P = 0.1661).
- **Geographic Region (Supplementary Figure S2 C):** Prevalence varied across geographic regions, ranging from 9% (95% CI: 2%–31%) in the Western region and 9% (95% CI: 6%–14%) in Tabuk to 48% (95% CI: 22%–76%) in the Eastern region. These regional subgroup differences were statistically significant (χ^2 = 57.04, df = 7, P < 0.0001).
- **Diagnostic Tool (Supplementary Figure S2 D):** Prevalence differed by diagnostic criteria, with eGFR-based diagnosis reporting 45% (95% CI: 8%–88%), combined/records/ICD methods reporting 16% (95% CI: 12%–22%), and albuminuria-based diagnosis reporting 9% (95% CI: 5%–15%). The subgroup differences based on diagnostic tool were statistically significant (χ^2 = 6.06, df = 2, P = 0.0483).

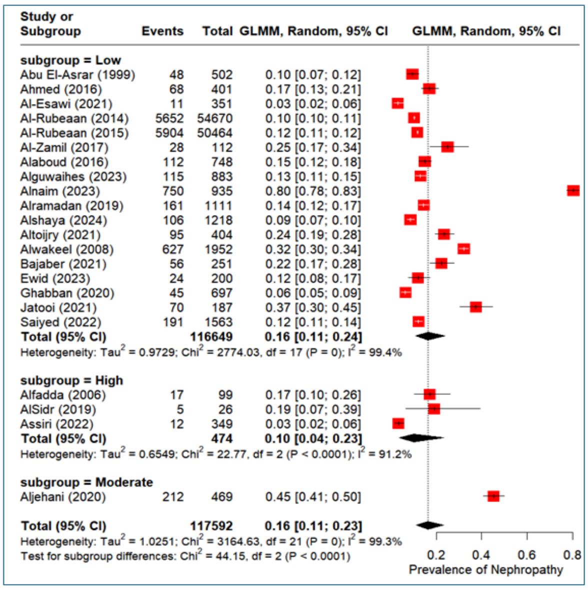

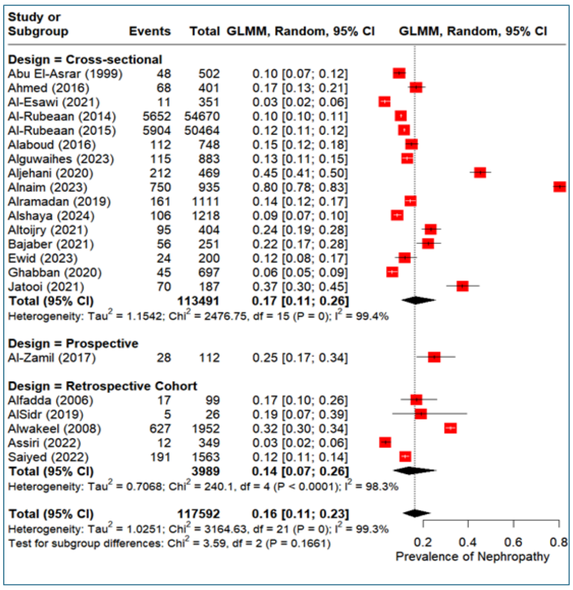

Supplementary figure 2 A

Supplementary figure 2 B

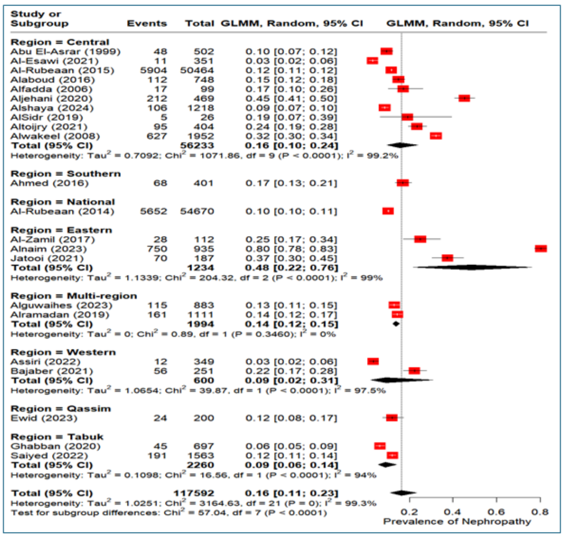

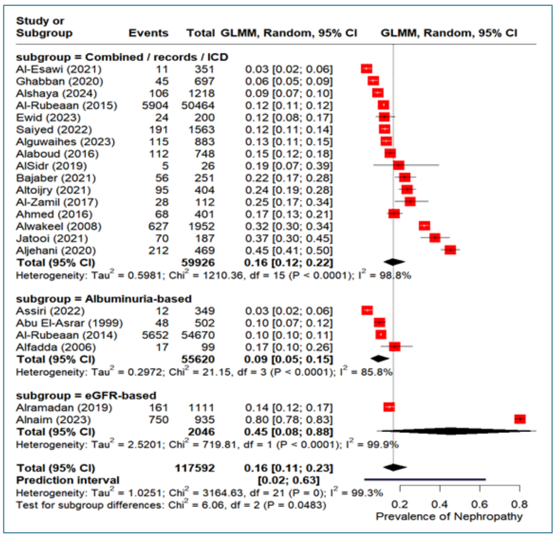

Supplementary figure 2 C

Supplementary figure 2 D

Figure S2 A-C. Subgroup analyses of diabetic nephropathy prevalence among Saudi by (A) risk of bias, (B) study design, (C) geographic region, and diagnostic tool (D)

Sensitivity analysis of diabetic nephropathy

The Baujat plot identifies Study 11 (Al-Rubeaan, 2015) as the dominant outlying study, contributing overwhelmingly to overall statistical heterogeneity and having the highest influence on the pooled estimate:

- Primary Driver of Heterogeneity: Study 11 (Al-Rubeaan, 2015) exhibits an extreme squared Pearson residual (> 8.0) and a substantial overall influence score (~0.65), making it the single largest contributor to model variation and pooled outcome variance.
- Secondary Influential Studies:
    - Study 3 (Alaboud, 2016) and Study 17 (Assiri, 2022) show moderate contributions to heterogeneity (squared Pearson residuals between 2.5 and 3.0) with influence scores around 0.13–0.14.
    - Study 10 (Al-Rubeaan, 2014) demonstrates a minor contribution to heterogeneity (~1.9 squared Pearson residual) with a low influence score (~0.09).
- Origin Cluster: Most of the remaining studies cluster tightly in the lower-left corner (near the origin), indicating that their individual contributions to overall study heterogeneity and influence on the final pooled estimate are minimal.

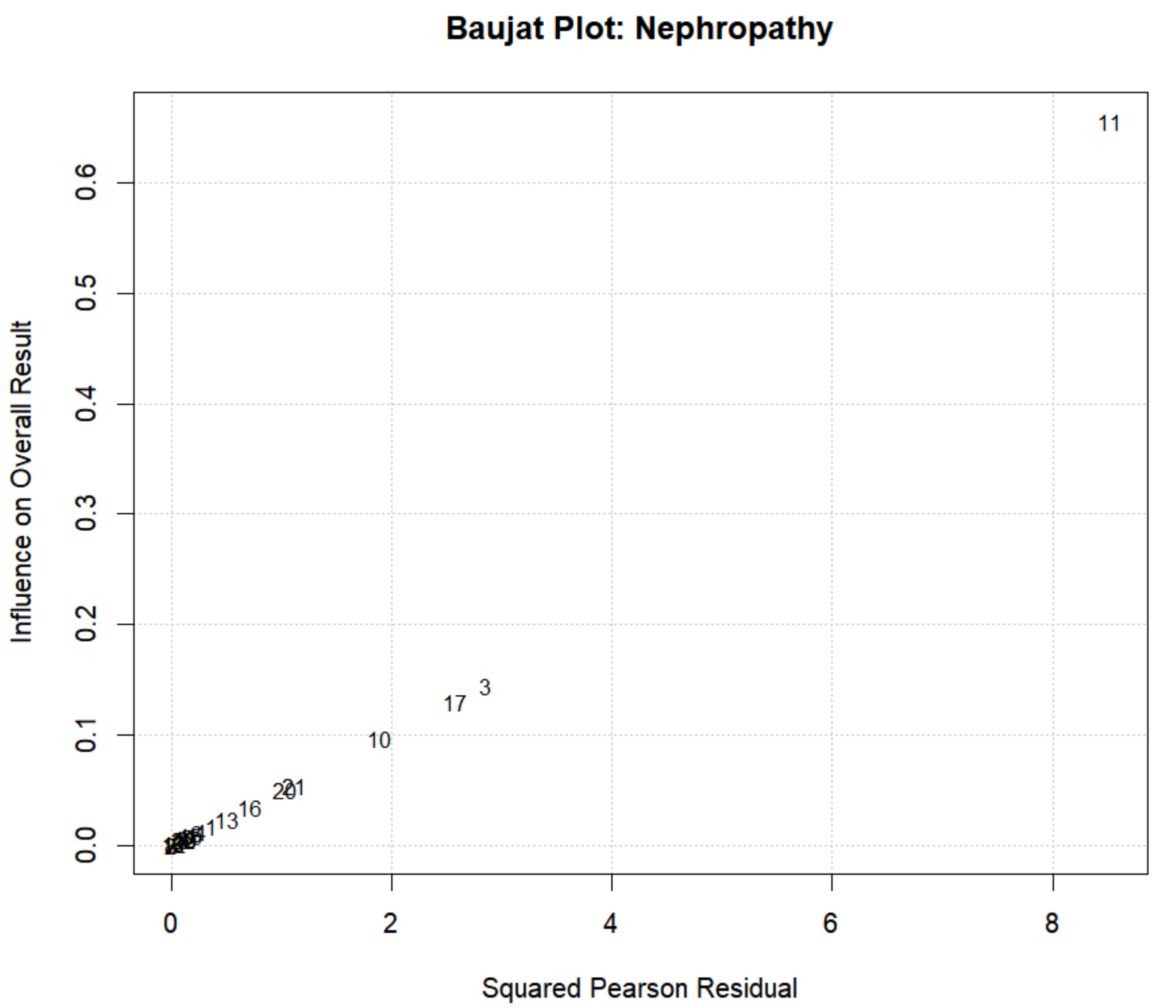

Figure S2 E: Baujat plot of included studies assessing the pooled prevalence of nephropathy among patients with type 2 diabetes mellitus.

The diagnostic plots identify Study 11 (Al-Rubeaan, 2015) as a significant outlier and the single most influential study in the meta-analysis:

- **Residuals & Influence Metrics (rstudent, dffits, cook.d):** Study 11 displays an extreme positive studentized residual (≈ 3.9), a peak DFFITS value (≈ 0.8), and a Cook's distance surpassing 0.4, confirming that it exerts strong influence and leverage over the pooled estimate.
- **Heterogeneity Impact (tau2.del, QE.del):** Excluding Study 11 results in a sharp drop in total residual variance (τ^2 decreases from > 1.0 down to ≈ 0.6) and

Cochrane's Q test statistic (QE), identifying it as the main driver of heterogeneity in the model. Secondary dips are also noticeable when omitting Study 3 (Alaboud, 2016) and Study 17 (Assiri, 2022).

- **Variance & Weight Metrics (cov.r, hat, weight):** Study 11 exhibits a sharp drop in covariance ratio (cov.r down to ≈ 0.6), indicating its strong impact on model precision. Leverage (hat) and overall study weighting remain relatively uniform across all 22 studies. **Supplementary figure S2 F**

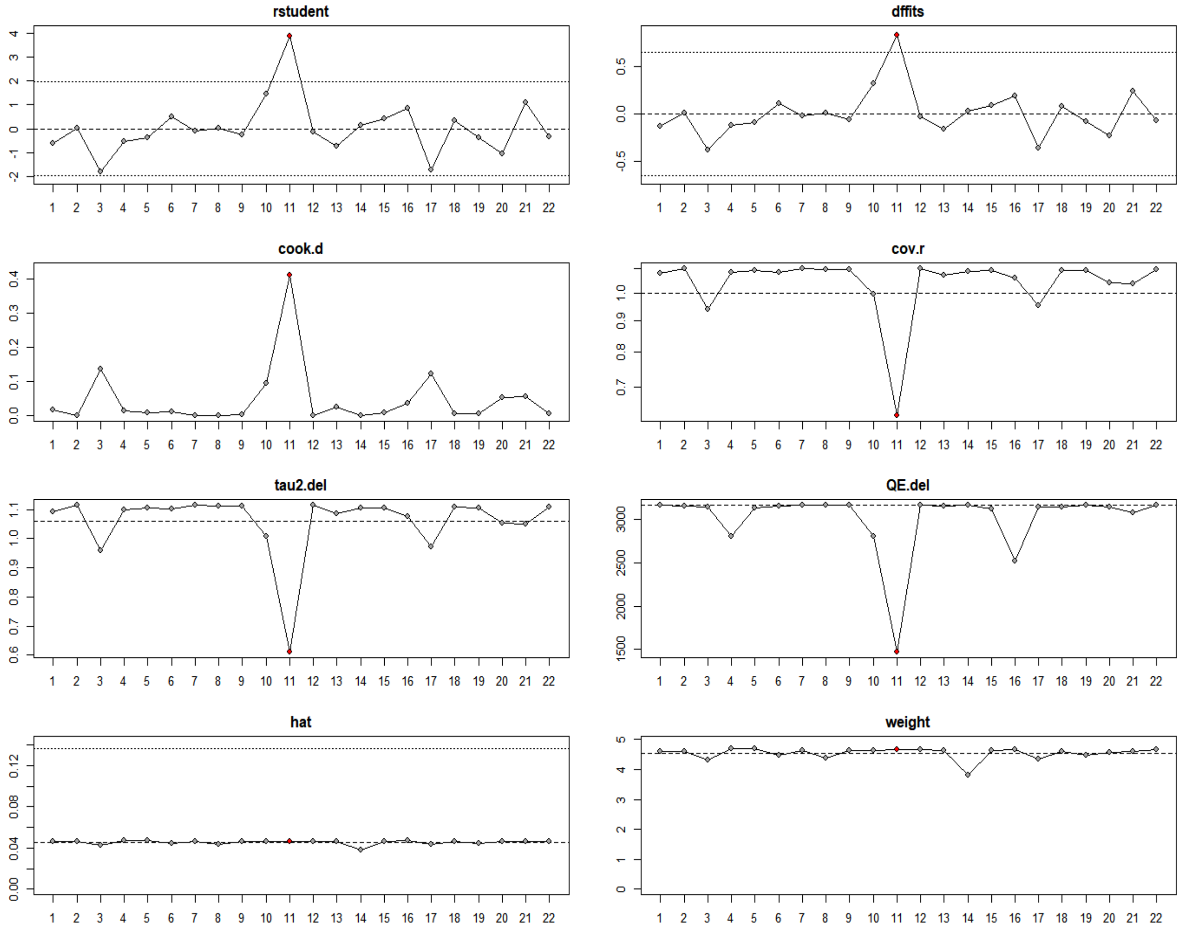

Figure S2 F: Influence analysis and diagnostic plots for meta-analysis of retinopathy prevalence in type 2 diabetes mellitus

The leave-one-out sensitivity plot demonstrates overall stability of the pooled prevalence estimate, as omitting any single study does not significantly shift the recalculated overall logit prevalence from the baseline reference line (dashed red line at approximately -1.6):

- **Primary Influential Shifts:**
    - Omitting Alnaim (2023) causes the most notable downward shift in overall logit prevalence (to approximately -1.75), indicating that this study reported a higher prevalence that pulled the overall pooled estimate upward.
    - Omitting Al-Esawi (2021) or Assiri (2022) produces minor upward shifts in logit prevalence (to approximately -1.53), reflecting their influence toward a lower pooled effect estimate.
- **Overall Robustness:** The recalculated logit prevalence estimates across all leave-one-out iterations remain tightly clustered around the baseline reference line, with wide and continuously overlapping 95% confidence intervals. This confirms that the final pooled prevalence estimate for diabetic nephropathy is robust and not excessively dependent on any individual study. **Supplementary figure S2 G**

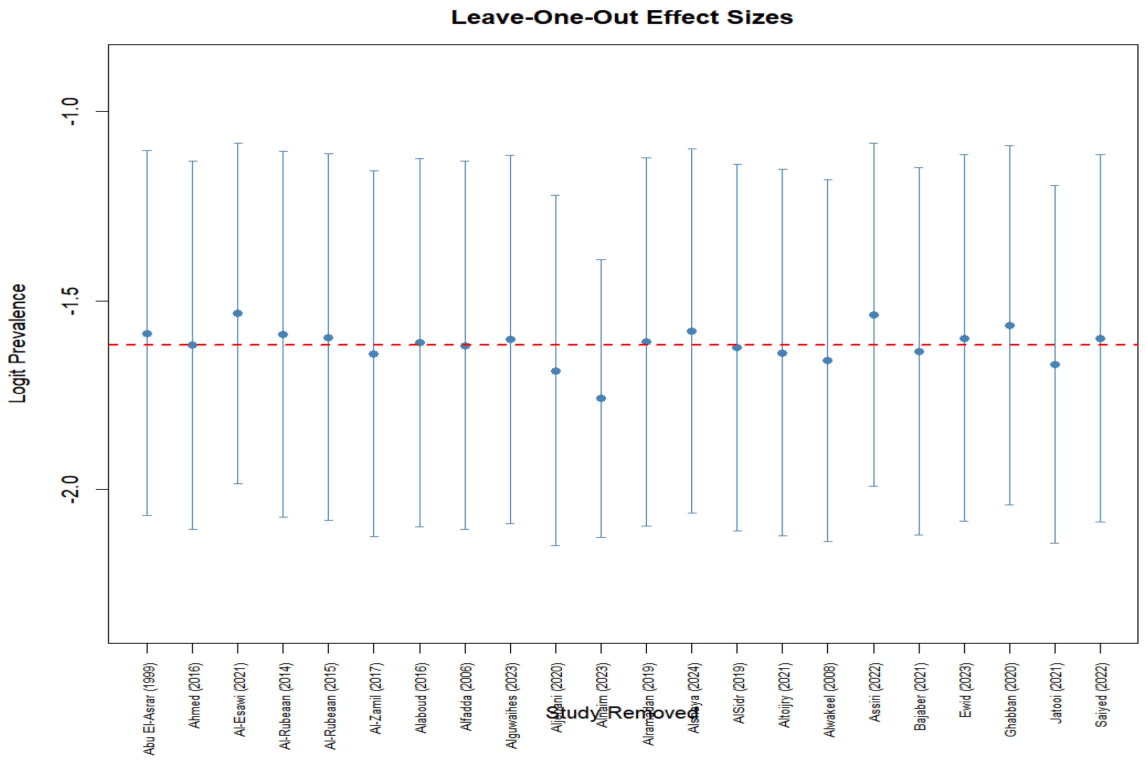

Figure S2 G: Leave-one-out sensitivity analysis of the pooled logit prevalence of nephropathy

The leave-one-out I^2 plot demonstrates that overall statistical heterogeneity remains extreme $I^2 > 99\%$ across all iterations, though key studies exert a measurable influence on reducing overall variation:

- Primary Contributors to Heterogeneity:
  - Omitting Al-Rubeaan (2014) or Al-Rubeaan (2015) produces the most significant drop in statistical heterogeneity, reducing I^2 from the baseline (dashed blue line at ~99.74%) down to approximately 99.38%.
  - Omitting Alnaim (2023) causes a secondary noticeable reduction, dropping I^2 to around 99.55%.
- Systemic Heterogeneity: Despite the observed drops upon excluding these specific studies, I^2 remains consistently above 99.3% across all leave-one-out scenarios. This indicates that the high degree of heterogeneity is a structural characteristic of the body of literature as a whole—likely due to clinical variations in diagnostic thresholds, study settings, and patient cohorts—rather than an artifact of a single study. Supplementary figure S2 H

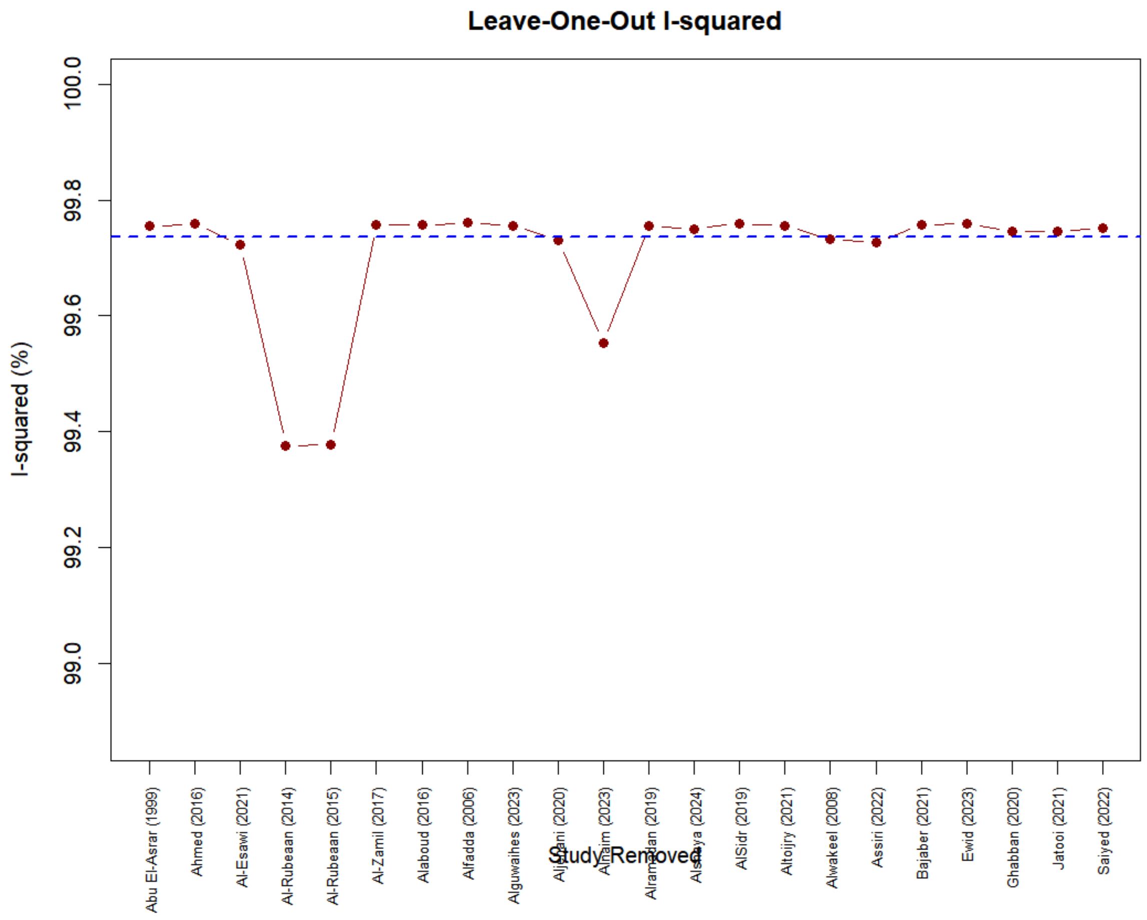

Figure S2 H: Leave-one-out sensitivity analysis of heterogeneity (i-squared) for the pooled prevalence of nephropathy among patients with type 2 diabetes mellitus

Meta regression of diabetic nephropathy prevalence

The meta-regression scatter plots illustrate the relationship between nephropathy prevalence (%) and continuous study-level covariates, with circle sizes reflecting sample size weighting:

- **Positive Associations:**
    - **Hypertension (HTN %), Obesity (%), and Dyslipidemia (%):** Each shows a clear upward regression slope, demonstrating that studies with higher proportions of patients with hypertension, obesity, or dyslipidemia reported higher nephropathy prevalence.

- **Publication Year:** Displays a slight upward trend over time.

- **Inverse / Negative Trends:**
  - **DM Duration (years):** Shows a steep downward slope, indicating lower reported nephropathy prevalence in study populations with longer mean diabetes duration.
  - **Mean Age (years) & HbA1c (%):** Both show a moderate downward trend relative to nephropathy prevalence.

- **Neutral / Minimal Trends:**
  - **Smoking (%) & JBI Quality Score (%):** Exhibit relatively flat regression lines, indicating little to no linear association with nephropathy prevalence across the included cohorts. **Supplementary figure S2 I**

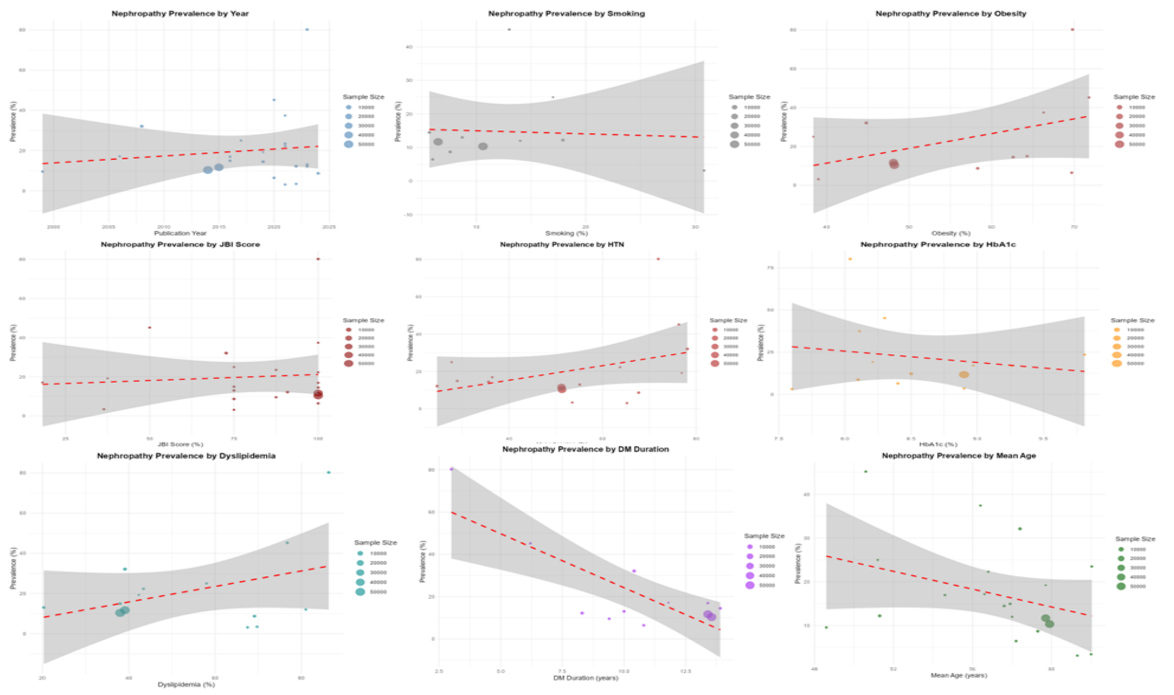

Figure S2 I: Meta-Regression analysis of nephropathy prevalence by study-level moderators (publication year, smoking, obesity, JBI score, hypertension, HbA1c, dyslipidemia, dm duration, and mean age) in type 2 diabetes mellitus

Univariate meta-regression of potential predictors of nephropathy, the results indicate that DM duration and female proportion were significantly associated with nephropathy. DM duration showed a significant negative association with nephropathy (β = −0.2539,

SE = 0.0706, 95% CI: −0.4112 to −0.0966, p = 0.0049) and explained 52.5% of the between-study heterogeneity. Female proportion also showed a significant positive association (β = 0.0357, SE = 0.0148, 95% CI: 0.0048 to 0.0667, p = 0.0259), accounting for 18.8% of the heterogeneity. Region explained 43.1% of the heterogeneity but was not statistically significant (p = 0.4333). The remaining moderators, including diagnostic modality, dyslipidemia, HbA1c, hypertension, JBI score, mean age, obesity, risk of bias, smoking, study design, and publication year, were not significantly associated with nephropathy (all $p > 0.05$). Residual heterogeneity remained very high across all models, with residual I^2 values ranging from 98.9% to 99.9%, indicating that substantial between-study variability remained unexplained by the individual moderators. Supplementary Table 2

Table S2: Univariate meta-regression of potential predictors of nephropathy

Study-level moderator	No. of studies	β	SE	95% CI	p-value	R^2 (%)	Residual I^2 (%)
**DM duration**	12	−0.2539	0.0706	−0.4112 to −0.0966	**0.0049**	52.5	99.7
**Diagnostic modality**	22	0.6973	0.5274	−0.4066 to 1.8013	0.2018	19.8	99.2
**Dyslipidemia (%)**	14	0.0132	0.0172	−0.0242 to 0.0506	0.4574	0.0	99.9
**Female (%)**	22	0.0357	0.0148	0.0048 to 0.0667	**0.0259**	18.8	99.7
**HbA1c**	13	−0.0980	0.6648	−1.5613 to 1.3653	0.8855	0.0	99.5
**Hypertension (%)**	16	0.0179	0.0158	−0.0161 to 0.0519	0.2775	2.5	99.8
**JBI score (%)**	22	0.0068	0.0095	−0.0131 to 0.0267	0.4839	0.0	99.7
**Mean age**	20	−0.0818	0.0464	−0.1792 to 0.0156	0.0946	9.8	99.5
**Obesity (%)**	12	0.0441	0.0300	−0.0227 to 0.1110	0.1719	9.6	99.9
**Region**	22	0.7882	0.9722	−1.3300 to 2.9063	0.4333	43.1	98.9
**Risk of bias**	22	0.5255	0.6651	−0.8666 to 1.9175	0.4393	3.7	99.7
**Smoking (%)**	11	−0.0267	0.0364	−0.1089 to 0.0555	0.4814	0.0	99.5
**Study design**	22	0.4931	1.1265	−1.8647 to 2.8510	0.6665	0.0	99.8
**Year**	22	0.0082	0.0371	−0.0692 to 0.0857	0.8265	0.0	99.7

After simultaneous adjustment for mean age and female proportion, mean age was no longer statistically associated with nephropathy prevalence (β = −0.0535, 95% CI −0.1449 to 0.0379, P = 0.234). Female proportion remained statistically significant. The coefficient was positive (β = 0.0270, 95% CI 0.0022 to 0.0518, P = 0.0344), indicating that higher female representation at the study level was associated with higher reported nephropathy estimates after adjustment for mean age. The parsimonious model had an R^2 of 28.5%. This means that the two moderators together explained 28.5% of the observed between-study variation. However, residual heterogeneity remained very high, with I^2 = 99.4%.

Diabetic Neuropathy

Publication bias of diabetic retinopathy prevalence [Figure S3 X]

The funnel plot displays marked asymmetry relative to the overall pooled effect estimate (vertical dashed line at approximately -1.2), indicating potential publication bias or significant small-study effects:

- **Asymmetry and Outlier Distribution:** A large proportion of the included studies lie outside the pseudo-95% confidence bounds (white triangular area), reflecting substantial between-study heterogeneity. The studies are widely scattered on both sides of the mean, with extreme outliers extending to lower outcome values (near -3.8) as well as higher values (above 2.0).
- **Small-Study Effects:** Smaller studies with larger standard errors (lower portion of the vertical axis) show wide dispersion away from the central estimate rather than forming a typical, symmetrical inverted funnel.
- **Interpretation:** This visual imbalance points to publication bias or substantial clinical and methodological differences across studies, such as variations in diagnostic tools, screening methods, or patient population characteristics.

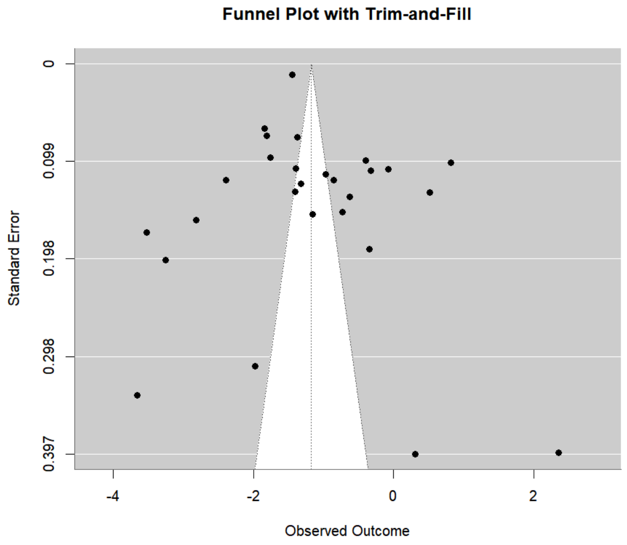

Figure 3S X: Funnel plot of included studies assessing the pooled prevalence of neuropathy among patients with type 2 diabetes mellitus.

Subgroup analysis of diabetic neuropathy prevalence [**Figure S3 S**]

Subgroup analyses were performed to assess the prevalence of diabetic neuropathy based on risk of bias, study design, geographic region, and diagnostic tools:

- Risk of Bias (Supplementary Figure S3 A): The pooled prevalence of diabetic neuropathy was highest in high-risk studies at 36% (95% CI: 15%–63%), followed by low-risk studies at 22% (95% CI: 15%–33%) and moderate-risk studies at 21% (95% CI: 3%–68%). The test for subgroup differences showed no statistically significant variation ($\chi^2 = 1.05$, df = 2, P = 0.5903).
- Study Design (Supplementary Figure S3 B): Pooled prevalence was 24% (95% CI: 15%–36%) in cross-sectional studies and 20% (95% CI: 9%–37%) in retrospective cohort studies. Differences across study designs were not statistically significant ($\chi^2 = 0.23$, df = 1, P = 0.6290).
- Geographic Region (Supplementary Figure S3 C): Prevalence varied across regions, ranging from 7% (95% CI: 3%–18%) in Tabuk to 63% (95% CI: 56%–69%) in Makkah (Western region). These regional subgroup differences were statistically significant ($\chi^2 = 365.71$, df = 9, P < 0.0001).
- Diagnostic Tool (Supplementary Figure S3 D): Studies using a validated instrument yielded a pooled prevalence of 32% (95% CI: 25%–40%), whereas clinical examinations or records showed a prevalence of 22% (95% CI: 13%–34%). The difference between diagnostic approaches was not statistically significant ($\chi^2 = 2.39$, df = 1, P = 0.1224).

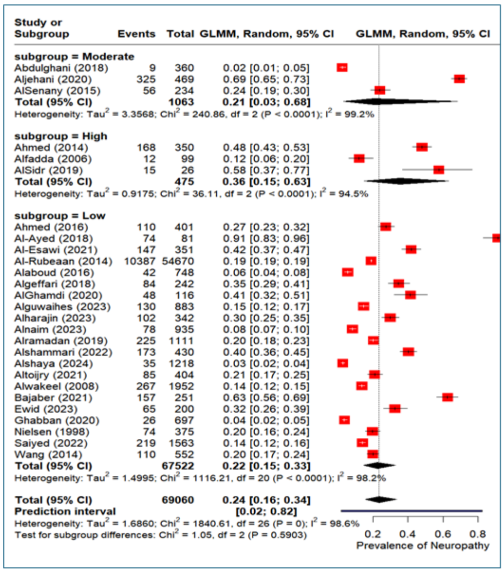

Supplementary figure 3 A

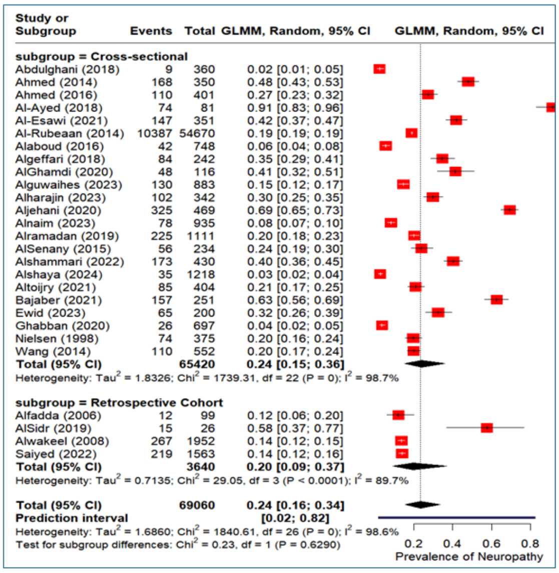

Supplementary figure 3 B

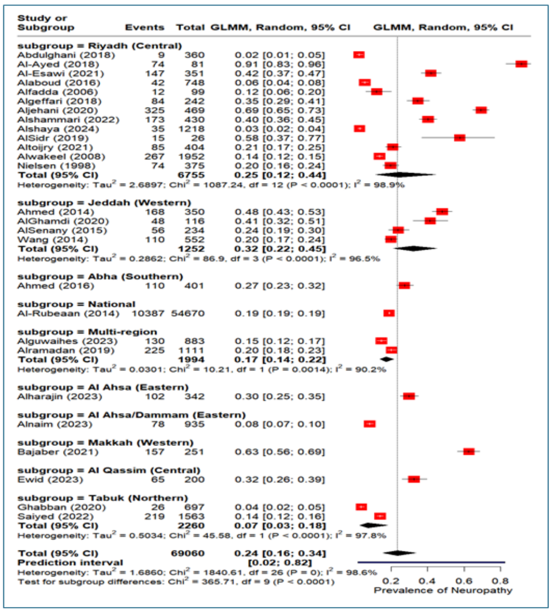

Supplementary figure 3 C

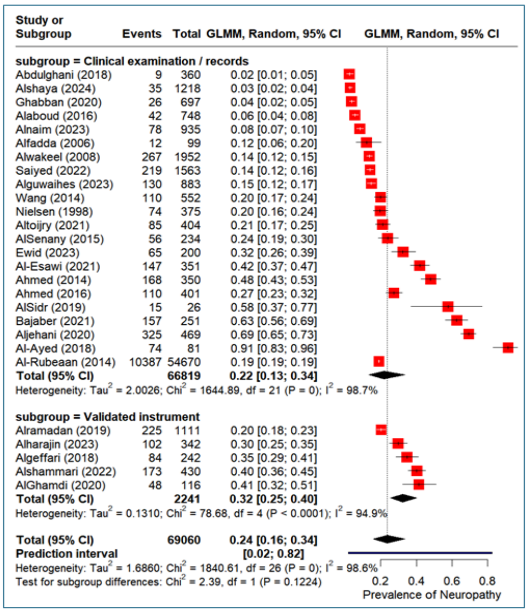

Supplementary figure 3 D

Figure 3S A-C. Subgroup analyses diabetic neuropathy prevalence by (A) risk of bias, (B) study design, (C) geographic region, and diagnostic tool (D).

Sensitivity analysis of diabetic neuropathy prevalence

The Baujat plot identifies **Study 4 (Al-Ayed, 2018)** as the dominant outlier contributing most significantly to overall heterogeneity and having the largest influence on the pooled outcome:

- **Primary Driver of Heterogeneity: Study 4 (Al-Ayed, 2018)** shows the highest contribution to statistical heterogeneity (squared Pearson residual = 6.9) and the greatest overall influence score (= 0.3).
- **Secondary Influential Studies:**

  Study 1 (Abdulghani, 2018) and Study 19 (AlSidr, 2019) demonstrate notable contributions to heterogeneity (squared Pearson residuals between 3.3 and 3.5) with moderate influence scores (≈ 0.13). Study 15 (Alramadan, 2019) and Study 25 (Nielsen, 1997) show moderate contributions (squared Pearson residuals ≈ 2.4 to 2.5) and influence scores around 0.09–0.10.

- **Origin Cluster:** Most of the remaining studies cluster close to the origin (lower-left quadrant), indicating minimal individual impact on overall study heterogeneity and model stability. **Supplementary Figure S3 E**

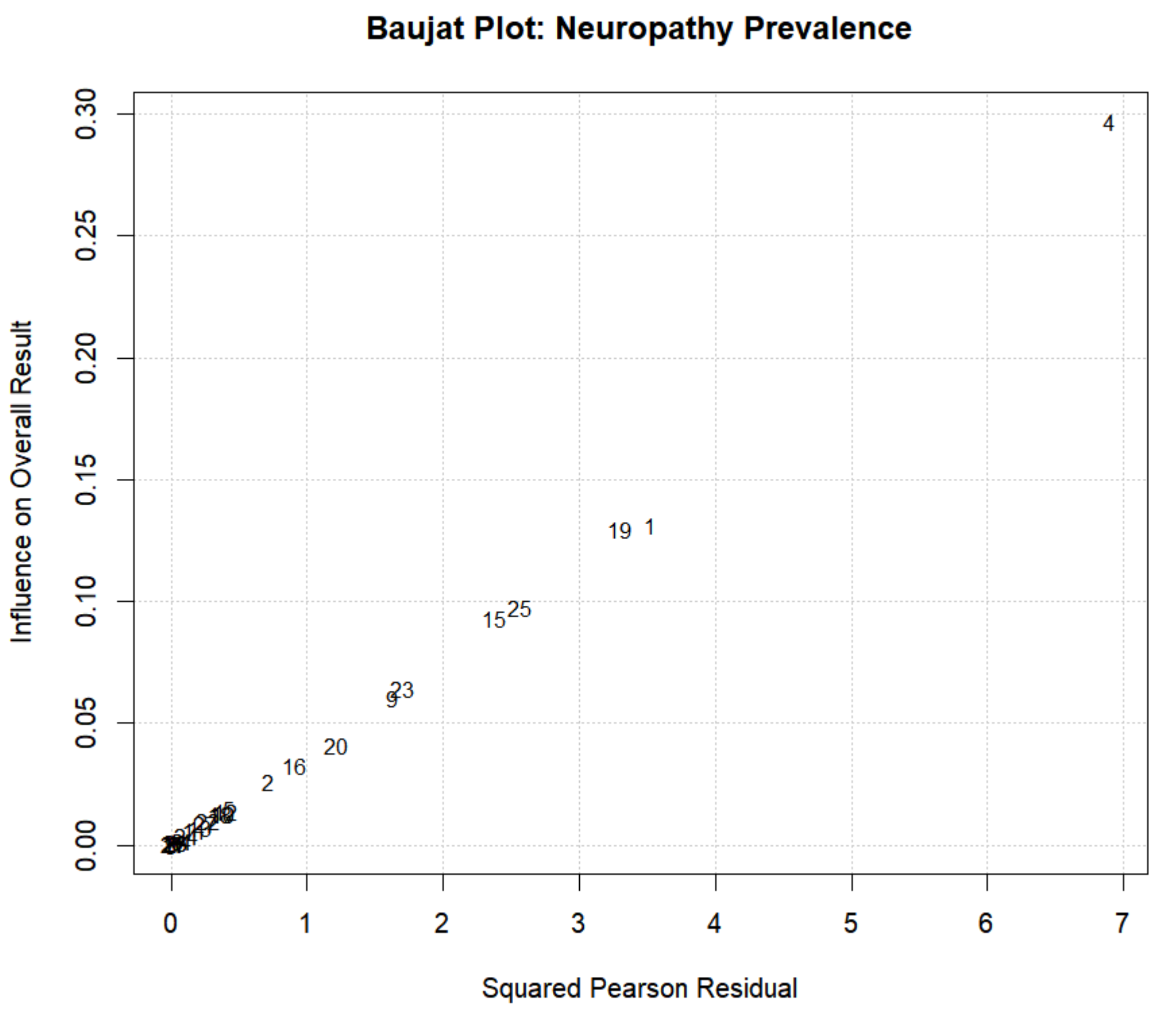

Figure S3 E: **Baujat plot of included studies assessing the pooled prevalence of diabetic neuropathy among patients with type 2 diabetes mellitus**

The diagnostic influence plots confirm that Study 4 (Al-Ayed, 2018) is the dominant outlier and most influential study in the meta-analysis, with secondary contributions from Study 1, Study 15, and Study 19:

- **Residuals & Influence Metrics (rstudent, dffits, cook.d):** Study 4 (Al-Ayed, 2018) exhibits a peak studentized residual (≈ 3.1), approaching the upper threshold for DFFITS (≈ 0.58), and a distinct peak in Cook's distance (≈ 0.23), indicating strong influence on the overall pooled outcome. Secondary peaks in Cook's distance and DFFITS are observed for Study 1 (Abdulghani, 2018), Study 15 (Alramadan, 2019), and Study 19 (AlSidr, 2019).

- **Heterogeneity & Variance Impact (tau2.del, QE.del, cov.r):** Omitting Study 4 leads to the sharpest drop in total residual variance (τ^2 decreases from > 1.6 to ≈ 1.28) and covariance ratio (cov.r drops to ≈ 0.79), showing its substantial role in driving statistical variability. Omitting Study 15 produces the largest single reduction in Cochrane's Q statistic (QE.del drops to ≈ 1350), with noticeable secondary drops for Study 4, Study 19, and Study 23 (Ewid, 2023).

- **Leverage & Weight Distribution (hat, weight):** Leverage values (hat) and study weights remain balanced and uniform across all 27 included studies (weight ≈ 3.5), confirming that influence is driven by outcome disparity rather than excessive sample weighting. **Supplementary figure 3E**

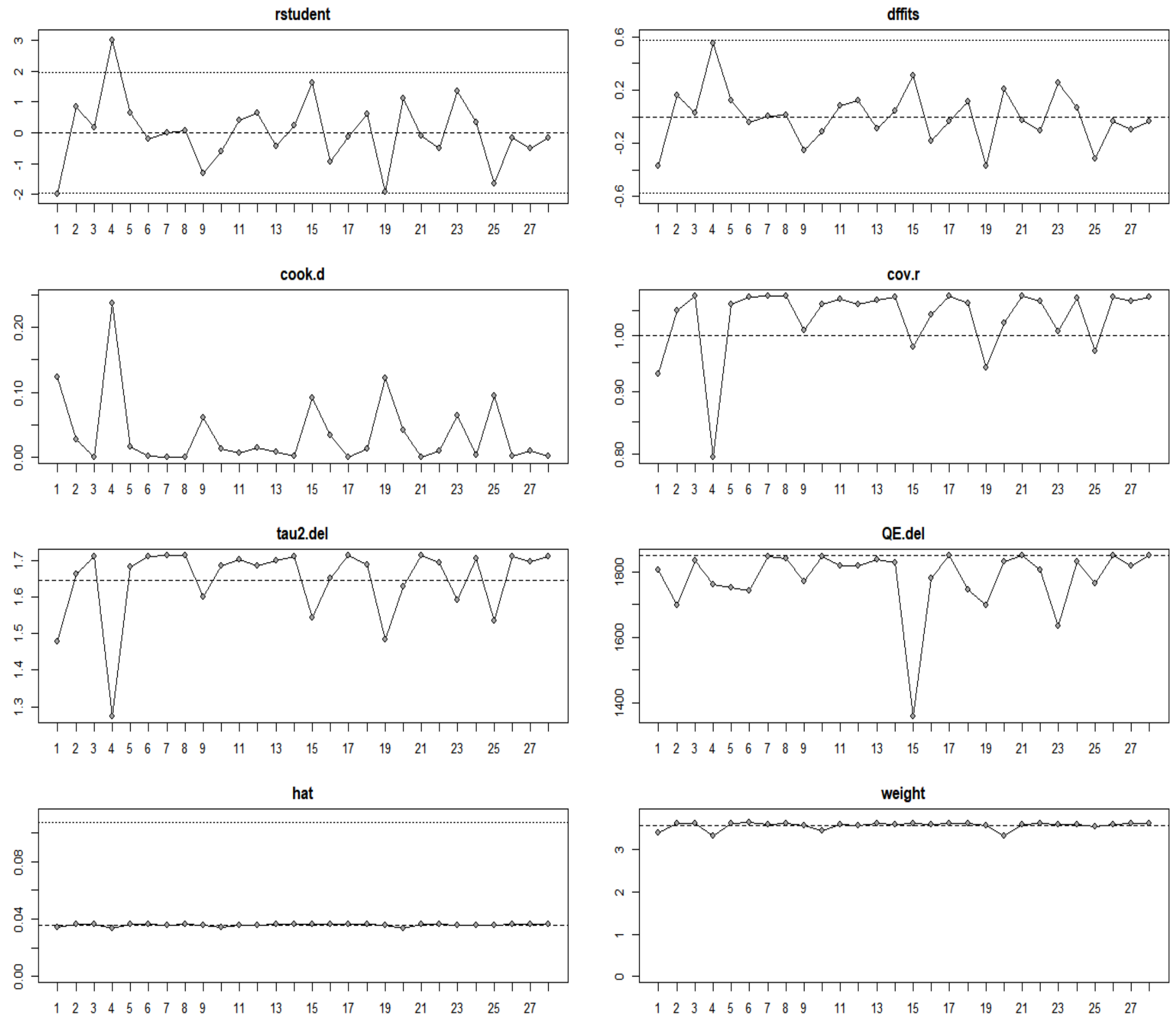

Figure S3E: Influence analysis and diagnostic plots for meta-analysis of neuropathy prevalence in type 2 diabetes mellitus

The leave-one-out sensitivity plot confirms the overall stability of the pooled prevalence estimate, as sequentially removing any single study does not substantially shift the recalculated logit prevalence from the baseline reference line (dashed red line at approximately -1.18):

- **Primary Influential Shifts:**
    - Omitting **Al-A**yed (2018) produces the most noticeable downward shift in recalculated logit prevalence (to approximately -1.30), demonstrating that this study reported a higher prevalence that pulled the overall pooled estimate upward.
    - Omitting Aljehani (2020) or Bajaber (2021) also results in minor downward shifts (to around -1.25).
    - Omitting Abdulghani (2018), Alaboud (2016), or Alshaya (2024) causes slight upward shifts in logit prevalence (to approximately -1.08 to -1.10).
- **Overall Robustness:** Despite minor fluctuations, all recalculated leave-one-out point estimates remain tightly bound around the overall estimate line, and all 95% confidence intervals overlap broadly. This indicates that no single study disproportionately distorts the final pooled prevalence estimate for diabetic neuropathy. **Supplementary figure S3 F**

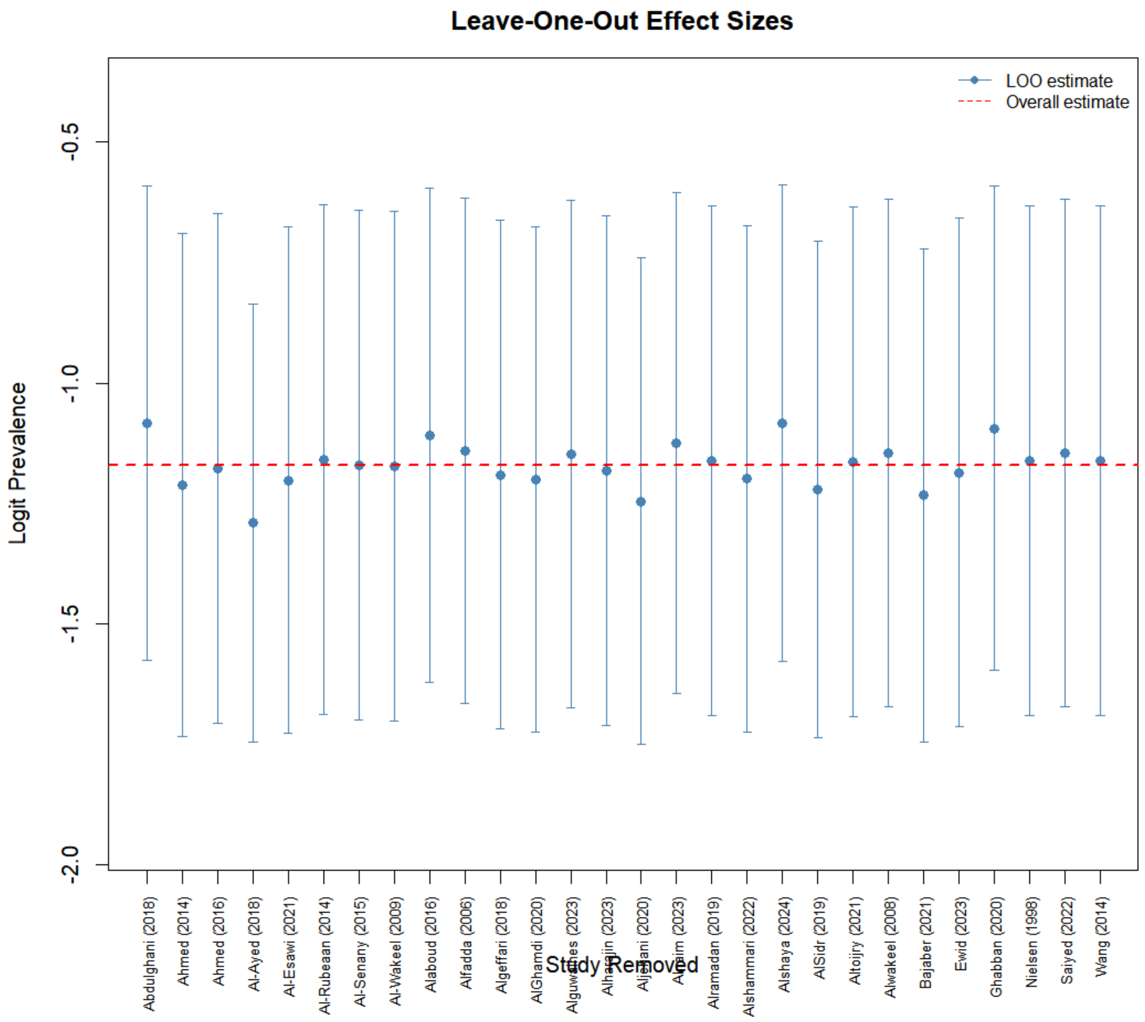

Figure S3 F: Leave-one-out sensitivity analysis of the pooled logit prevalence of neuropathy

The leave-one-out I^2 plot demonstrates that overall statistical heterogeneity remains extreme ($I^2 > 99.2\%$) regardless of which study is sequentially omitted, though key studies exert a measurable influence on the overall variation:

- **Primary Contributors to Heterogeneity:**

Omitting Al-Rubeaan (2014) produces the most pronounced drop in statistical heterogeneity, reducing I^2 from the baseline (dashed blue line at 99.55%) down to approximately 99.20%. Omitting Al-Ayed (2018) causes a secondary noticeable drop, reducing I^2 to around 99.43%. Minor dips are also observed when excluding Alshaya (2024), Aljehani (2020), and Abdulghani (2018) (≈99.50%).

- **Systemic Heterogeneity:** Across all 27 leave-one-out iterations, I^2 consistently remains above 99.2%. This confirms that the high level of heterogeneity is a persistent, structural feature of the broader body of literature—driven by differences in diagnostic criteria, clinical settings, and patient demographics across studies—rather than an artifact driven by a single outlier. **Supplementary figure S3 H**

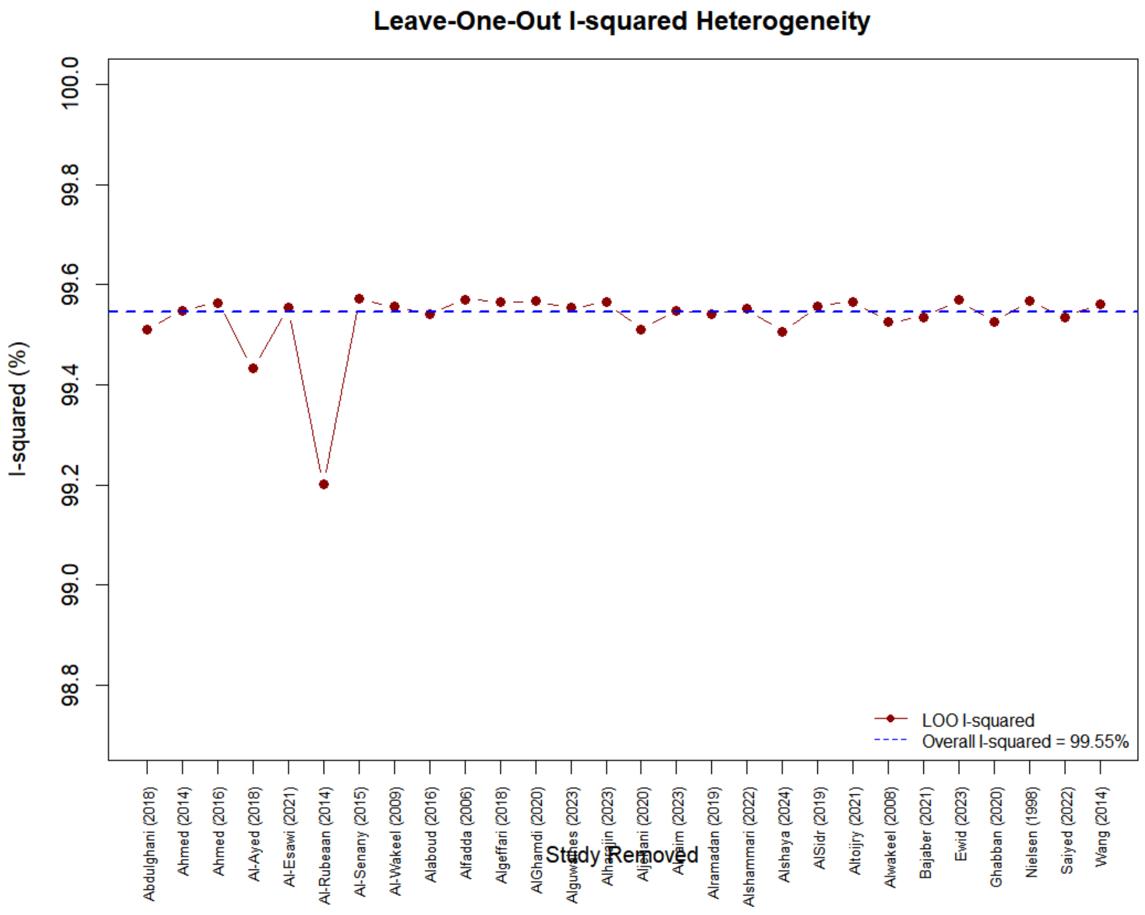

Figure S3 H: Leave-One-Out Sensitivity Analysis of Heterogeneity (I-squared) for the Pooled Prevalence of neuropathy among Patients with Type 2 Diabetes Mellitus

Meta-regression analysis of diabetic neuropathy prevalence

The meta-regression scatter plots illustrate the relationship between diabetic neuropathy prevalence (%) and continuous study-level covariates, with circle sizes representing sample size weighting:

- Positive Associations:
    - Smoking (%): Shows a strong, pronounced positive linear regression slope, indicating that studies with higher proportions of smokers reported significantly higher neuropathy prevalence.

- o Hypertension (HTN %) & Dyslipidemia (%): Both show clear positive trends, demonstrating higher reported neuropathy prevalence in cohorts with higher rates of hypertension and dyslipidemia.
- o DM Duration (years): Displays a modest upward slope, indicating a trend toward higher neuropathy prevalence with longer mean diabetes duration.

- Inverse / Negative Trends:
  - o Mean Age (years): Shows a consistent downward slope, suggesting lower reported neuropathy prevalence in older cohort averages.
  - o JBI Quality Score (%) & Obesity (%): Both demonstrate inverse relationships, with lower prevalence observed in studies with higher JBI methodological quality scores and higher obesity rates.
- Neutral / Minimal Trends:
  - o HbA1c (%): Displays an almost flat regression line (red dashed line), indicating that overall glycemic level was not strongly associated with linear variations in neuropathy prevalence across the included studies.

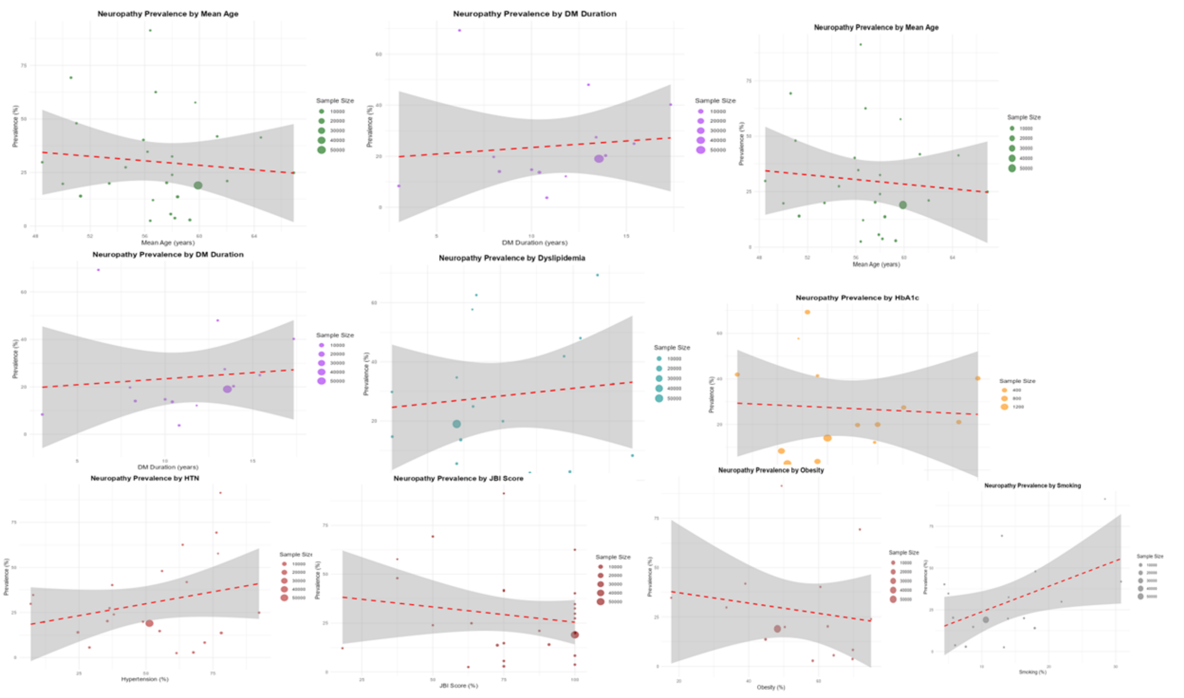

Figure S3 I: Meta-Regression Analysis of Neuropathy Prevalence by Study-Level Moderators (Publication Year, Smoking, Obesity, JBI Score, Hypertension, HbA1c, obesity Dyslipidemia, DM Duration, and Mean Age) in Type 2 Diabetes Mellitus

The univariate meta-regression identified smoking as the only statistically significant study-level moderator of diabetic neuropathy. Smoking was positively associated with the outcome ($\beta = 0.0932$, SE = 0.0431, 95% CI: 0.0013 to 0.1852, $p = 0.0472$) and explained 18.8% of the between-study heterogeneity. None of the other evaluated moderators, including DM duration, diagnostic modality, dyslipidemia, female proportion, HbA1c, hypertension, JBI score, mean age, obesity, region, risk of bias, study design, or publication year, showed a statistically significant association with diabetic neuropathy (all $p > 0.05$). Despite the association with smoking, substantial residual heterogeneity persisted across all models, with residual I2 ranging from 99.1% to 99.7%, indicating that most of the between-study variability remained unexplained by the individual study-level moderators. **Table S3**

Table S3: Univariate meta-regression of potential predictors of Diabetic Neuropathy

Moderator	N studies	Estimate	SE	95% CI	P value	R^2 (%)	τ^2 residual	I^2 residual (%)
**DM Duration**	13	0.0537	0.0819	−0.1266 to 0.2339	0.5258	0.0	1.1213	99.5
**Diagnostic Modality**	27	0.5540	0.6642	−0.8140 to 1.9220	0.4121	0.0	1.7348	99.5
**Dyslipidemia (%)**	16	0.0015	0.0174	−0.0358 to 0.0389	0.9306	0.0	1.9494	99.6
**Female (%)**	27	−0.0157	0.0184	−0.0536 to 0.0222	0.4010	0.0	1.7400	99.6
**HbA1c**	14	0.1540	0.5276	−0.9955 to 1.3036	0.7753	0.0	1.6493	99.1
**Hypertension (%)**	22	0.0110	0.0132	−0.0166 to 0.0386	0.4167	0.0	1.8905	99.6
**JBI Score (%)**	27	−0.0071	0.0111	−0.0300 to 0.0158	0.5286	0.0	1.7563	99.5
**Mean Age**	25	−0.0412	0.0713	−0.1886 to 0.1063	0.5690	0.0	1.8353	99.5
**Obesity (%)**	15	−0.0235	0.0253	−0.0782 to 0.0312	0.3696	0.0	2.2425	99.7

**Region**	27	0.1172	2.0809	−4.2731 to 4.5074	0.9558	0.0	2.1079	99.2
**Risk of Bias**	27	−0.6506	0.8595	−2.4246 to 1.1234	0.4564	0.0	1.8174	99.6
**Smoking (%)**	17	0.0932	0.0431	0.0013 to 0.1852	0.0472	18.8	1.7408	99.6
**Study Design**	27	−0.2165	0.7430	−1.7468 to 1.3137	0.7731	0.0	1.7797	99.5
**Year**	27	0.0134	0.0450	−0.0792 to 0.1060	0.7677	0.0	1.7822	99.5

Note: R^2 represents the proportion of between-study variance explained by the moderator. τ^2 represents residual between-study variance. I^2 represents residual heterogeneity. $^*p < 0.05$. ns, not significant.

Cerebrovascular stroke

Publication bias of diabetic retinopathy prevalence [Figure S4 X]

The funnel plot demonstrates substantial visual asymmetry around the overall pooled effect line (vertical dashed line at approximately -3.28), indicating marked between-study heterogeneity and potential publication bias or small-study effects:

- **Asymmetry and Outlier Distribution:** A significant proportion of the included studies lie outside the pseudo-95% confidence bounds (white triangular area). Studies with higher precision (smaller standard errors near the top) are split across the distribution, with a cluster situated to the right (higher observed outcome prevalence, between -2.7 and -2.15) and others situated to the left (lower observed outcome prevalence, extending down to -4.45).
- **Small-Study Effects:** Studies with lower precision/larger standard errors (bottom half of the plot) show considerable scatter away from the central pooled estimate rather than forming a symmetrical, inverted funnel shape.
- **Interpretation:** This pronounced visual asymmetry points toward potential publication bias, variations in sample size, or clinical and methodological diversity across included cohorts (e.g., differences in stroke diagnostic criteria, neuroimaging confirmation, patient age groups, or study settings).

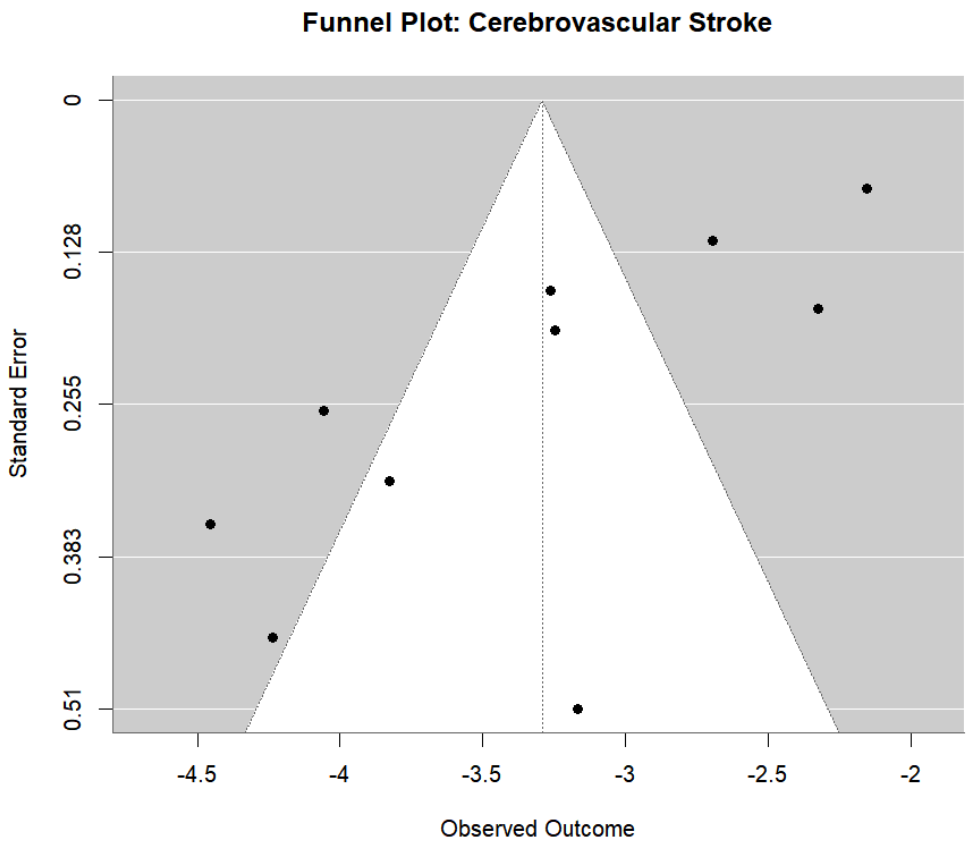

Figure S4 X: Funnel plot included studies assessing the pooled prevalence of cerebrovascular stroke among patients with type 2 diabetes mellitus

Subgroup analysis Cerebrovascular stroke prevalence **[Figure S 4 S]**

Subgroup analyses were conducted to evaluate the prevalence of cerebrovascular stroke in Saudi Arabia based on risk of bias, study design, geographic region, and diagnostic tools:

- Risk of Bias (Supplementary Figure 4A): The pooled prevalence of cerebrovascular stroke was 4% (95% CI: 2%–6%) in studies with low risk of bias, 4% (95% CI: 1%–10%) in high risk, and 2% (95% CI: 1%–4%) in moderate risk. The test for subgroup differences was not statistically significant ($\chi^2 = 2.05$, df = 2, P = 0.3586).
- Study Design (Supplementary Figure 4B): Retrospective cohort studies reported a higher pooled prevalence at 10% (95% CI: 9%–11%) compared to cross-sectional

studies at 3% (95% CI: 2%–5%). This difference across study designs was statistically significant ($\chi^2 = 23.72$, df = 1, $P < 0.0001$).

- Geographic Region (Supplementary Figure S4 C): Pooled prevalence differed across geographic regions, ranging from 1% (95% CI: 0%–2%) in Tabuk (Northern region) to 5% (95% CI: 3%–7%) in Riyadh (Central region), with multi-region studies reporting 3% (95% CI: 2%–4%). Subgroup differences by geographic region were statistically significant ($\chi^2 = 9.90$, df = 2, $P = 0.0071$).
- Diagnostic Tool (Supplementary Figure S4 D): Studies utilizing imaging confirmation showed a pooled prevalence of 5% (95% CI: 3%–7%), while those relying on clinical or unspecified diagnostic types reported 3% (95% CI: 2%–6%). The test for subgroup differences between diagnostic methods was not statistically significant ($\chi^2 = 1.58$, df = 1, $P = 0.2086$).

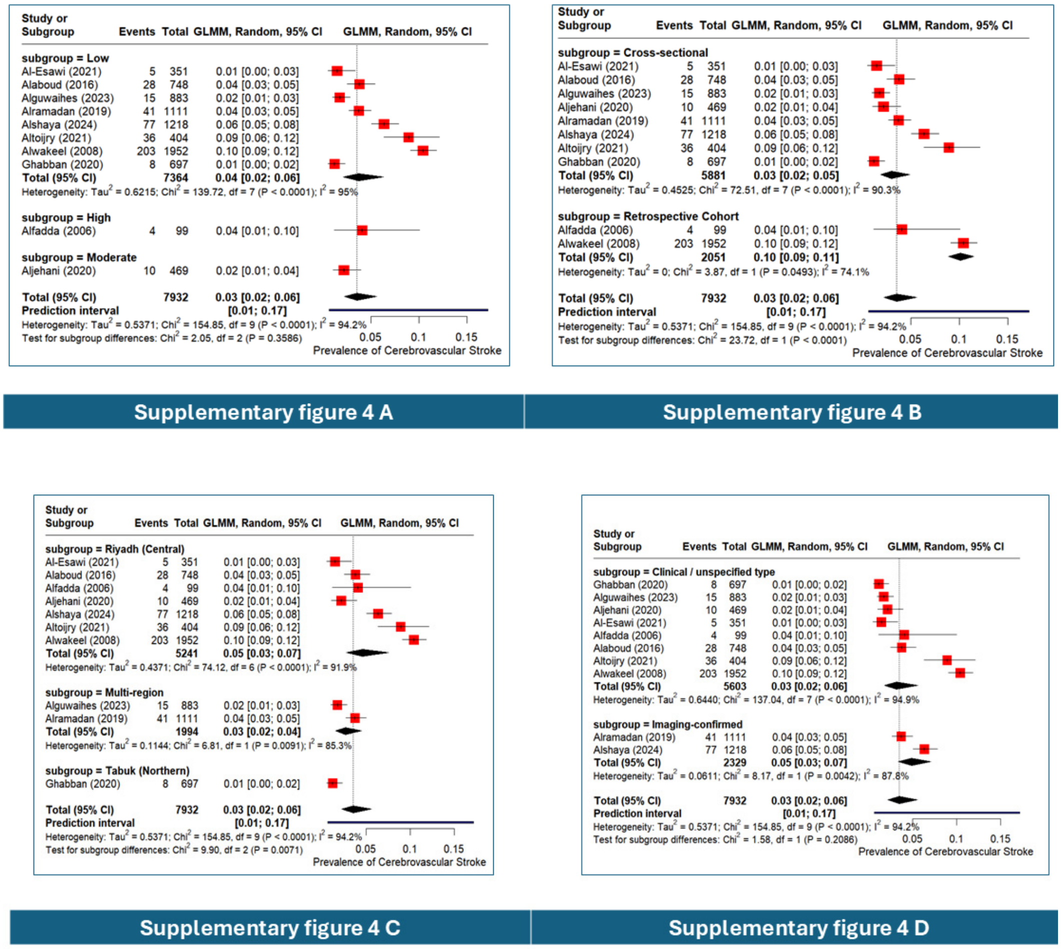

Figure S4 A-C. Subgroup analyses cerebrovascular stroke prevalence by (A) risk of bias, (B) study design, (C) geographic region, and diagnostic tool (D)

Sensitivity analysis Cerebrovascular stroke prevalence

Here is the Baujat plot assessment for the included meta-analysis studies assessing the pooled prevalence of cerebrovascular stroke among patients with type 2 diabetes mellitus:

The Baujat plot identifies **Study 4 (Alguwaihes)** as the single most influential outlier, driving most of the overall heterogeneity and exerting the strongest influence on the overall pooled estimate:

- **Primary Driver of Heterogeneity:** Study 4 (Alguwaihes) exhibits the highest squared Pearson residual (≈6.7) and the greatest overall influence score (≈0.30), making it the dominant contributor to model variability.

- **Secondary Influential Studies:** Study 1 (Al-Esawi) and Study 18 (labeled as 18 on the plot, corresponding to Alshaya, Study 7 in the subset list) show moderate contributions to heterogeneity (squared Pearson residuals between 3.1 and 3.4) with influence scores around 0.13. Study 13 and Study 24 exhibit moderate contributions to heterogeneity (squared Pearson residuals ≈2.3–2.4) with influence scores of approximately 0.09–0.10.

- **Origin Cluster:** Most of the remaining included studies cluster near the origin (lower-left corner), indicating that their individual contributions to statistical heterogeneity and overall influence on the pooled stroke prevalence estimate are minimal. **Supplementary figure S4 E**

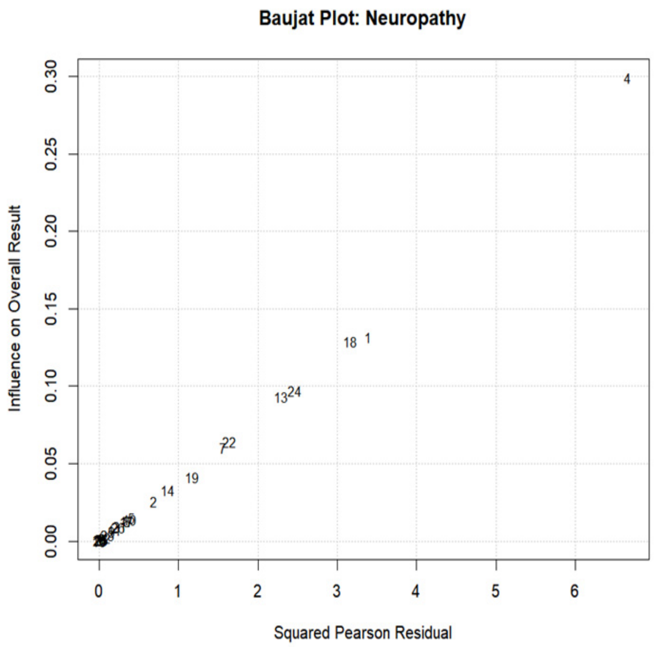

Figure S4 E: Baujat plot included studies assessing the pooled prevalence of Cerebrovascular stork among patients with type 2 diabetes mellitus

The diagnostic influence plots confirm that **Study 4** is the primary outlier and most influential study in the meta-analysis, with secondary influential contributions observed from **Study 1**, **Study 13**, **Study 18**, and **Study 24**:

- **Residuals & Influence Metrics (rstudent, dffits, cook.d):**

  Study 4 exhibits a peak studentized residual (≈3.0), an upper-bound DFFITS value (≈0.58), and a distinct peak in Cook's distance (≈0.23), indicating strong statistical influence and leverage over the pooled stroke prevalence estimate. Notable secondary peaks in Cook's distance and DFFITS are observed for Study 1, Study 13, Study 18, and Study 24.

- **Heterogeneity & Variance Impact (tau2.del, QE.del, cov.r):**

Omitting Study 4 leads to the sharpest drop in total residual variance (τ^2 decreases from >1.7 down to ≈1.33) and covariance ratio (cov.r drops to ≈0.79), underscoring its role as the major driver of statistical heterogeneity. Omitting Study 13 produces the largest single drop in Cochrane's Q test statistic (QE.del drops to ≈1350), with secondary drops observed when excluding Study 2, Study 18, and Study 22.

- **Leverage & Weight Distribution (hat, weight):**

  Leverage values (hat) and individual study weights remain uniform and stable across all 27 studies (weight ≈ 3.7), confirming that influence is driven by variance in reported prevalence rates rather than disproportionate sample weighting. **Supplementary figure S4 F**

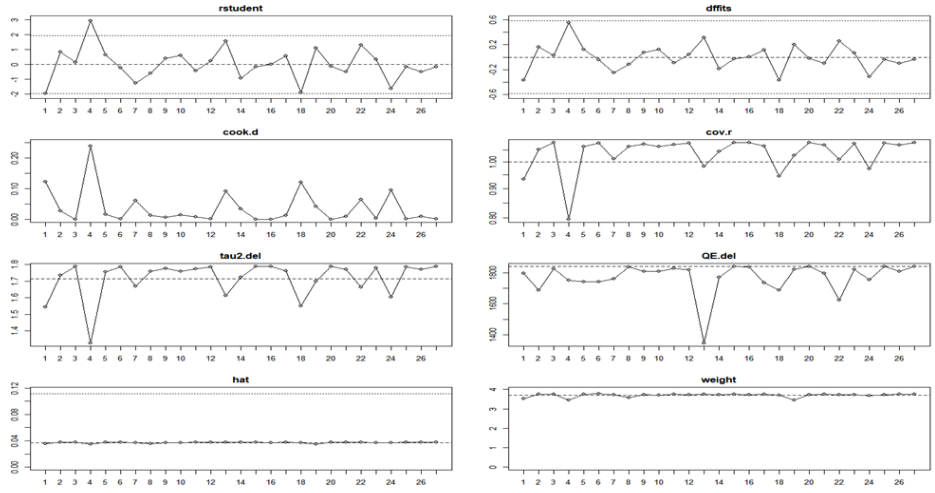

Figure S4 F: Influence Analysis and Diagnostic Plots for Meta-Analysis of Cerebrovascular stroke Prevalence in Type 2 Diabetes Mellitus

The leave-one-out sensitivity plot demonstrates high stability of the overall pooled prevalence estimate, as sequentially removing any single study does not meaningfully shift the recalculated logit prevalence from the baseline reference line (dashed red line at approximately -1.18): Primary Influential Shifts:Omitting Al-Ayed (2018) produces the

most pronounced downward shift in recalculated logit prevalence (to approximately -1.30), indicating that this study reported a higher prevalence that pulled the overall pooled estimate upward. Omitting Aljehani (2020) or Bajaber (2021) also results in minor downward shifts (to around -1.25). Omitting Abdulghani (2018), Alaboud (2016), or Ghabban (2020) causes slight upward shifts in logit prevalence (to approximately -1.08 to -1.10). Overall Robustness: The recalculated logit prevalence estimates across all leave-one-out iterations remain tightly clustered around the baseline reference line, with wide and consistently overlapping 95% confidence intervals. This confirms that the final pooled stroke prevalence estimate is robust and not excessively dependent on any single study. **Supplementary figure S4 G**

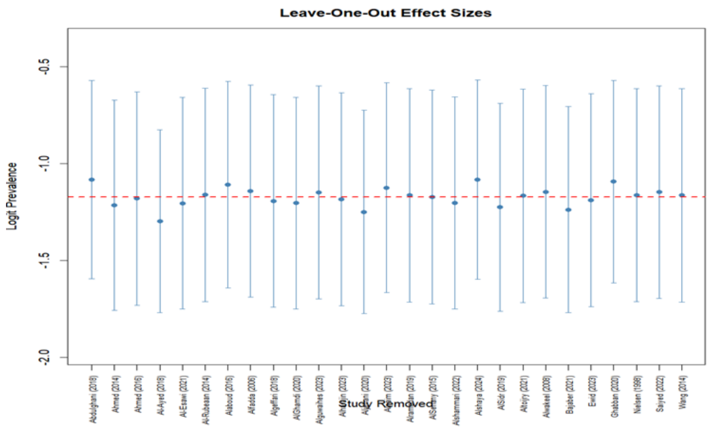

Figure S4 G: Leave-one-out sensitivity analysis of the pooled logit prevalence of Cerebrovascular stork

The leave-one-out I^2 plot demonstrates that overall statistical heterogeneity remains extreme ($I^2 > 99.2\%$) across all iterations, though specific studies exert a measurable influence on total model variability:

- **Primary Contributors to Heterogeneity:**

Omitting Al-Rubeaan (2014) produces the most significant drop in statistical heterogeneity, reducing I^2 from the baseline (dashed blue line at approximately 99.55%) down to around 99.21%. Omitting Al-Ayed (2018) causes a secondary noticeable drop, reducing I^2 to approximately 99.45%. Minor dips are also observed when sequentially excluding Abdulghani (2018), Aljehani (2020), and Alshaya (2024) (≈99.52%).

- **Systemic Heterogeneity:** Despite the minor reductions observed upon omitting these specific studies, I^2 remains consistently above 99.2% across all 27 leave-one-out iterations. This indicates that the extreme statistical heterogeneity is an inherent, structural characteristic of the body of literature as a whole—likely driven by clinical differences in stroke classification, neuroimaging verification, and population demographics—rather than an artifact of any single study. **Supplementary figure S4 H**

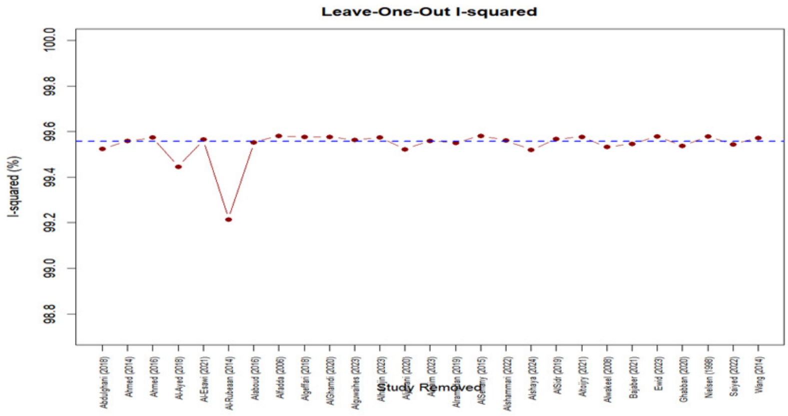

Figure S4 H: Leave-one-out sensitivity analysis of heterogeneity (i-squared) for the pooled prevalence of cerebrovascular stork among patients with type 2 diabetes mellitus

Meta-regression analysis cerebrovascular stroke prevalence

The meta-regression scatter plots illustrate the relationship between cerebrovascular stroke prevalence (%) and continuous study-level covariates, with bubble sizes indicating study sample size weighting:

- **Positive Associations:**
    - **Mean Age (years) & HbA1c (%):** Both demonstrate upward linear regression slopes, indicating that studies with older patient cohorts or higher mean HbA1c levels reported higher prevalence rates of cerebrovascular stroke.
    - **Hypertension (HTN %) & JBI Quality Score (%):** Both show positive slopes, reflecting higher stroke prevalence in study populations with higher hypertension rates as well as studies with higher methodological quality scores.
    - **DM Duration (years):** Displays a slight positive trend, suggesting a modest increase in stroke prevalence with longer mean diabetes duration.
- **Inverse / Negative Trends:**
    - **Publication Year:** Displays a clear downward slope over time, showing lower reported stroke prevalence in more recent publication years.
    - **Obesity (%) & Smoking (%):** Both exhibit downward regression slopes, indicating lower reported stroke prevalence in cohorts with higher proportions of obesity or smoking.
- **Neutral / Minimal Trends:**
    - **Dyslipidemia (%):** Displays a nearly flat regression line with a broad confidence band, indicating little to no linear relationship with

cerebrovascular stroke prevalence across the included studies. **Supplementary figure S4 I**

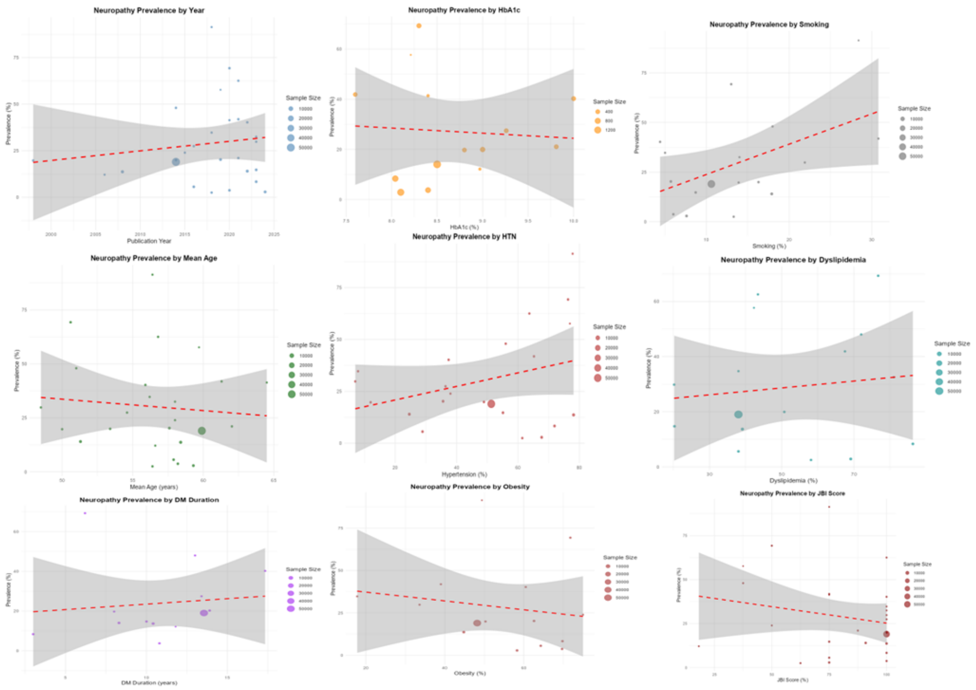

Figure S4 I: Meta-Regression Analysis of cerebrovascular stroke prevalence by study-level moderators (publication year, smoking, obesity, JBI score, hypertension, HbA1C, obesity dyslipidemia, dm duration, and mean age) in type 2 diabetes mellitus

The univariate meta-regression analyses found no statistically significant association between any of the evaluated study-level moderators and cerebrovascular accident, with all p-values> 0.05. HbA1c explained the largest proportion of between-study heterogeneity ($R^2 = 24.5\%$), followed by region ($R^2 = 21.3\%$) and study design ($R^2 = 14.6\%$), although none of these associations were statistically significant. The remaining moderators, including DM duration, diagnostic modality, dyslipidemia, female proportion, hypertension, JBI score, mean age, obesity, risk of bias, smoking, and publication year, explained little or none of the observed heterogeneity ($R^2 = 0$-4.9%) and were not significantly associated with cerebrovascular accident. Residual heterogeneity remained substantial across all models, with residual I^2 ranging from 84.7% to 96.4%, indicating that the examined study-level moderators did not adequately explain the

variability in cerebrovascular accident estimates between studies. **Supplementary table 4**

Table S4: Univariate meta-regression of potential predictors of cerebrovascular accident

Moderator	N studies	Estimate (β)	SE	95% CI	P value	R^2 (%)	τ^2 residual	I^2 residual (%)
**DM duration**	6	0.0767	0.1585	−0.3635 to 0.5168	0.6539	0.0	0.7753	94.4
**Diagnostic modality**	10	0.4097	0.6331	−1.0503 to 1.8697	0.5357	0.0	0.6291	94.3
**Dyslipidemia (%)**	6	−0.0027	0.0184	−0.0539 to 0.0485	0.8892	0.0	0.7892	95.8
**Female (%)**	10	0.0157	0.0147	−0.0182 to 0.0497	0.3157	0.3	0.5717	95.0
**HbA1c**	6	0.7199	0.4272	−0.4663 to 1.9061	0.1673	24.5	0.5144	84.7
**Hypertension (%)**	7	0.0072	0.0168	−0.0360 to 0.0503	0.6876	0.0	0.5878	95.0
**JBI score (%)**	10	0.0049	0.0116	−0.0218 to 0.0316	0.6825	0.0	0.6199	94.6
**Mean age**	9	0.0767	0.0889	−0.1336 to 0.2870	0.4171	0.0	0.5615	95.2
**Obesity (%)**	7	−0.0235	0.0289	−0.0977 to 0.0507	0.4524	0.3	0.6051	94.3
**Region**	10	0.6266	0.5728	−0.7280 to 1.9812	0.3102	21.3	0.4511	93.7
**Risk of bias**	10	−0.0834	1.0286	−2.5157 to 2.3489	0.9377	0.0	0.6864	96.4
**Smoking (%)**	6	−0.0281	0.0354	−0.1265 to 0.0702	0.4715	0.0	0.4212	90.6
**Study design**	10	0.8991	0.6059	−0.4981 to 2.2963	0.1761	14.6	0.4896	91.4
**Year**	10	−0.0513	0.0438	−0.1523 to 0.0498	0.2759	4.9	0.5452	92.2

Myocardial infarction and coronary artery disease

Publication bias of myocardial infarction and coronary artery disease prevalence [Figure S3 X]

The funnel plot displays noticeable visual asymmetry relative to the overall pooled effect line (vertical dashed line at approximately -2.12), indicating potential publication bias, small-study effects, or underlying study heterogeneity:

- **Asymmetry and Outlier Distribution:** A substantial portion of the included studies lie outside the pseudo-95% confidence bounds (white triangular area). High-precision studies (smaller standard errors near the top) are asymmetrical, with a prominent cluster situated to the right (higher observed outcomes, ranging between -1.8 and -1.1) and a few outliers on the far left (extending down to -4.6 and -5.85).
- **Small-Study Effects:** Studies with larger standard errors (lower portion of the vertical axis) show wide dispersion rather than forming a symmetrical inverted funnel shape.
- **Interpretation:** This visual imbalance suggests publication bias or substantial clinical and methodological variations across included studies, such as differences in diagnostic criteria for myocardial infarction/CAD, screening protocols, clinical settings, or underlying population risk profiles.

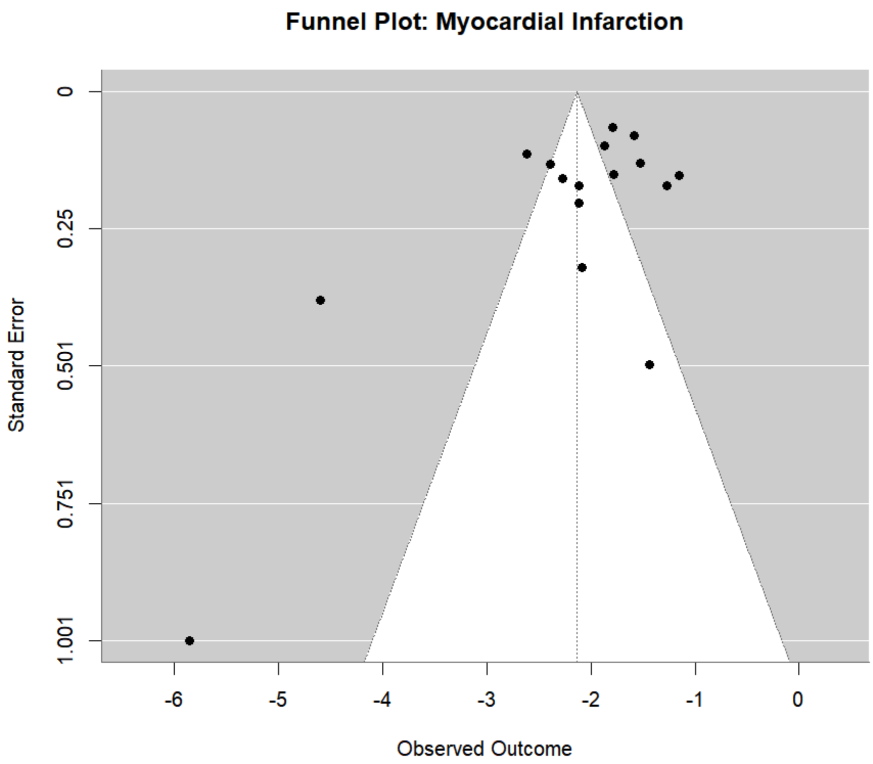

Figure 5X S: Funnel plot of included studies assessing the pooled prevalence of myocardial infarction and coronary artery disease among patients with type 2 diabetes mellitus

Subgroup analysis of myocardial infarction and coronary artery disease **[Figure S5 S]**

Subgroup analyses were conducted to evaluate the prevalence of myocardial infarction (MI) based on risk of bias, study design, geographic region, and diagnostic criteria:

- Risk of Bias (Supplementary Figure 5B): Pooled prevalence of MI was highest in moderate-risk studies at 15% (95% CI: 9%–23%), followed by low-risk studies at 10% (95% CI: 6%–16%), and high-risk studies at 4% (95% CI: 0%–28%). Differences across risk-of-bias subgroups were not statistically significant ($\chi^2 = 2.35$, df = 2, P = 0.3090).
- Study Design (Supplementary Figure 5A): Pooled prevalence was 11% (95% CI: 7%–16%) in cross-sectional studies and 6% (95% CI: 1%–25%) in retrospective

cohort studies. The test for subgroup differences by study design showed no statistically significant variation ($\chi^2 = 0.54$, df = 1, P = 0.4605).

- Geographic Region (Supplementary Figure 5C): Prevalence varied significantly across geographic locations, ranging from 0% (95% CI: 0%–2%) in Taif and 1% (95% CI: 0%–2%) in Tabuk to 24% (95% CI: 19%–30%) in Jeddah. Regional subgroup differences were statistically significant ($\chi^2 = 104.63$, df = 6, P < 0.0001).
- Diagnosis (Supplementary Figure 5D): Studies evaluating broader CAD/CHD endpoints reported a significantly higher pooled prevalence of 20% (95% CI: 15%–25%) compared to those specifically measuring confirmed MI at 9% (95% CI: 5%–14%). Subgroup differences between diagnostic definitions were statistically significant ($\chi^2 = 8.65$, df = 1, P = 0.0033).

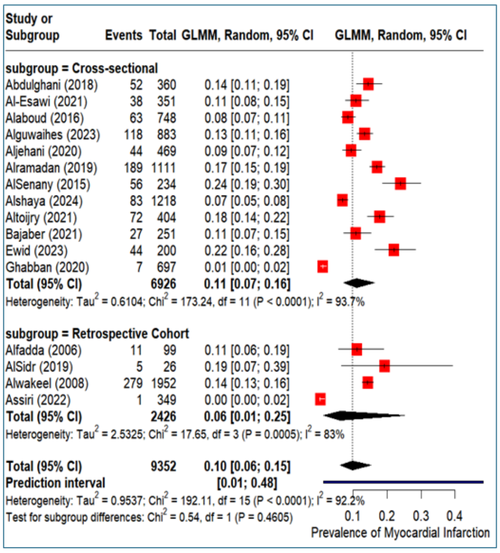

Supplementary figure 5 A

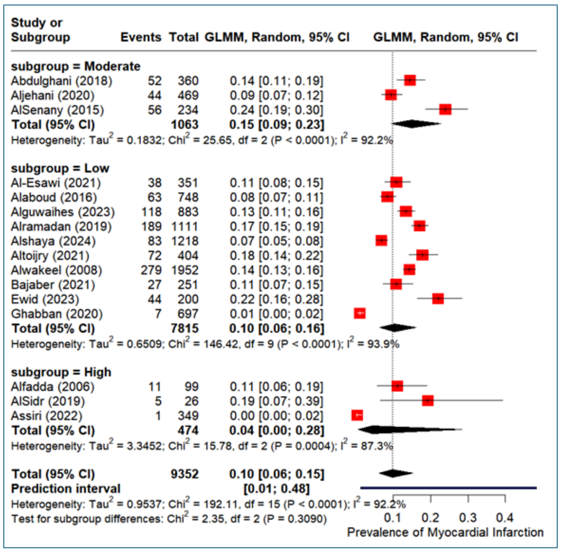

Supplementary figure 5 B

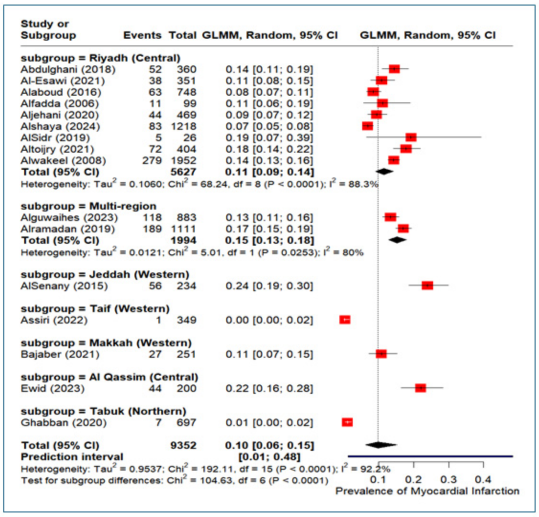

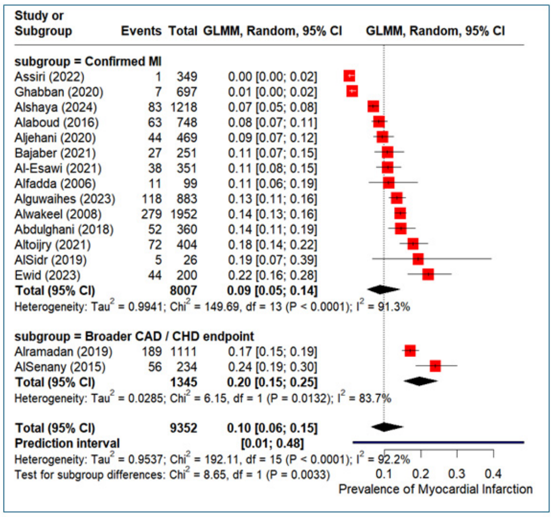

Supplementary figure 5 C	Supplementary figure 5 D

Figure S5A-C. Subgroup analyses myocardial infarction prevalence by (A) risk of bias, (B) study design, (C) geographic region, and diagnosis (D)

Sensitivity analysis of myocardial infarction and coronary artery disease

The Baujat plot identifies **Study 16 (Ghabban 2020)** as the single most influential outlier, driving most of the overall heterogeneity and exerting the strongest influence on the overall pooled MI prevalence estimate:

- **Primary Driver of Heterogeneity:** Study 16 (Ghabban 2020) exhibits the highest influence score (≈1.45) and a large squared Pearson residual (≈7.5), making it the dominant contributor to model variability. This is driven by an exceptionally low prevalence (7/697, 1.0%).
- **Secondary Influential Study:** Study 13 (Assiri 2022) shows the largest squared Pearson residual (≈7.8) with a moderate influence score (≈0.35), driven by the lowest prevalence in the dataset (1/349, 0.3%).
- **Tertiary Influential Studies:** Study 15 (Ewid 2023) and Study 8 (AlSenany 2015) exhibit mild contributions to heterogeneity (squared Pearson residuals ≈1.4) with

influence scores of approximately 0.04–0.05, driven by high prevalences (22.0% and 23.9% respectively) in small samples.

- **Origin Cluster:** The majority of the remaining included studies (Study 1–7, 9–12, 14) cluster near the origin (lower-left corner), indicating that their individual contributions to statistical heterogeneity and overall influence on the pooled MI prevalence estimate are minimal. **Supplementary figure S5 E**

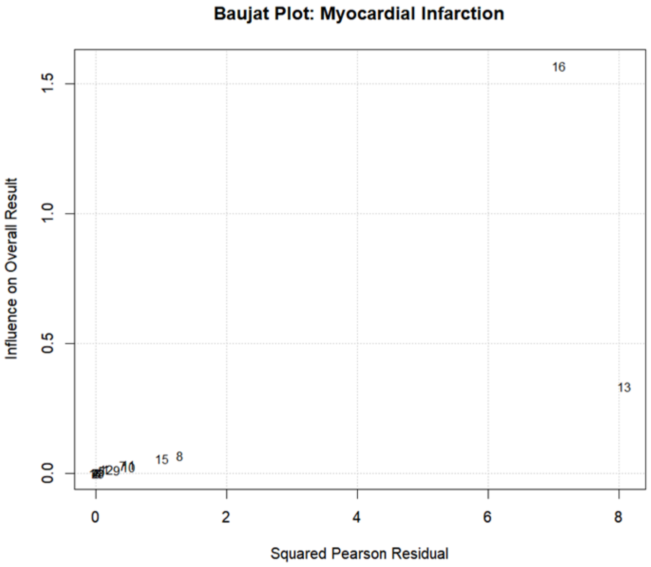

Figure S5 E: Baujat plot included studies assessing the pooled prevalence of Myocardial infarction and coronary artery disease among patients with type 2 diabetes mellitus.

The panel of influence diagnostics (Supplementary Figure 5F) confirms that the MI meta-analysis is dominated by a small number of studies, with **Study 13 (Assiri 2022)**, **Study 15 (Ewid 2023)**, and **Study 16 (Ghabban 2020)** emerging as the principal drivers of model instability:

- Primary Driver of Instability : Study 16 (Ghabban 2020): Exhibits the most extreme values across multiple diagnostics — the highest Cook's D (≈0.45), the largest DFFITS (≈0.30), the highest hat value (≈0.20), the largest cov.r deviation (≈1.05), and the most extreme tau^2.del (≈0.44). This confirms Ghabban (2020) as the single most influential study in the model, driven by its exceptionally low prevalence (7/697, 1.0%).

- Secondary Influential Study: Study 13 (Assiri 2022): Shows a strong DFFITS spike (≈−0.32), an elevated Cook's D (≈0.25), an elevated hat value (≈0.15), and a pronounced tau².del deviation (≈0.75), reflecting its extremely low prevalence (1/349, 0.3%). Its influence is amplified by the small number of MI events.
- Tertiary Influential Study — Study 15 (Ewid 2023): Displays moderate deviations in DFFITS (≈−0.20), Cook's D (≈0.10), and cov.r (≈1.02), driven by its high prevalence (44/200, 22.0%) in a small sample.
- Origin Cluster: The remaining studies (Study 1–7, 9–12, 14) cluster tightly around the reference lines across all eight diagnostic panels, indicating minimal individual influence on the pooled MI prevalence estimate. **Supplementary figure S5 F**

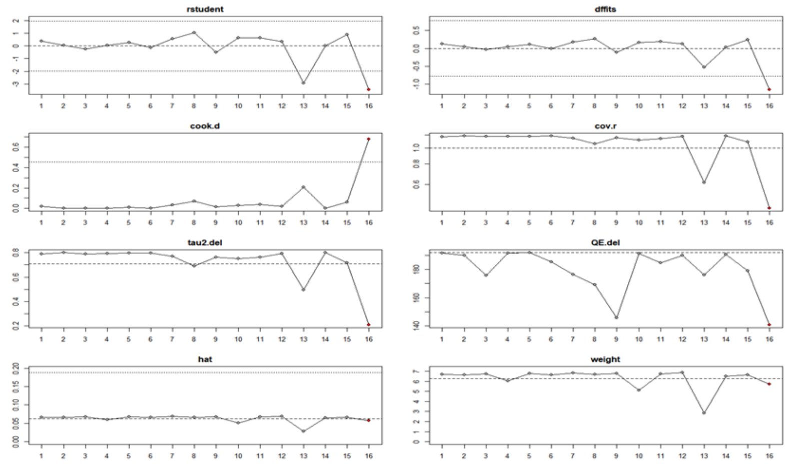

Figure S5 F: Influence analysis and diagnostic plots for meta-analysis of myocardial infarction and coronary artery disease prevalence in type 2 diabetes mellitus

The leave-one-out (LOO) effect size plot (Supplementary Figure 5G) confirms that the pooled MI prevalence estimate is robust for most studies, with only **two studies** materially altering the pooled logit prevalence when removed:

- Primary Influential Study — Ghabban (2020): When removed, the pooled logit prevalence shifts sharply upward from the reference line (≈−2.14) to approximately −1.90, the largest single-study displacement observed. This corresponds to the greatest single-study influence on the pooled estimate, consistent with the Baujat and influence diagnostics findings. The upward shift

reflects Ghabban's exceptionally low prevalence (1.0%), which was pulling the pooled estimate downward.

- Secondary Influential Study — Assiri (2022): When removed, the pooled logit prevalence shifts upward to approximately −2.00. This is the second-largest displacement, consistent with Assiri's extremely low prevalence (0.3%) exerting a downward pull on the pooled estimate.
- All Remaining Studies: The LOO point estimates for all other studies cluster tightly around the reference line (≈–2.14), with narrow, overlapping confidence intervals, indicating that no single one of them materially changes the pooled MI prevalence estimate. Supplementary **figure S5 G**

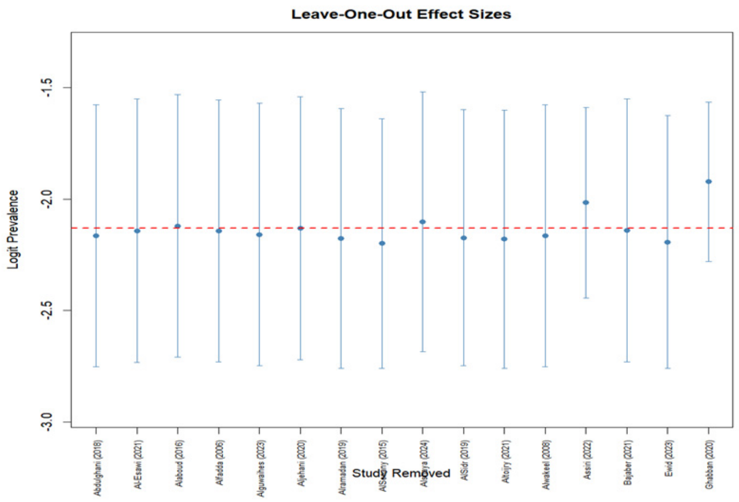

Figure S5 G: Leave-one-out sensitivity analysis of the pooled logit prevalence of Myocardial infarction and coronary artery disease

The leave-one-out I² plot (Supplementary Figure 5H) shows that between-study heterogeneity remains **persistently high across all iterations**, confirming that no single study is solely responsible for the observed heterogeneity — though one study does meaningfully reduce it:

- Primary Heterogeneity Driver : Ghabban (2020): When removed, I² drops sharply from the reference line (≈97.4%) to approximately 92.0% a reduction of about 5.4 percentage points, the largest single-study effect observed. This confirms Ghabban (2020) as the dominant contributor to statistical heterogeneity, consistent with its

extreme low prevalence (1.0%) identified in the Baujat plot and influence diagnostics.

- Secondary Contributor : Assiri (2022): When removed, I^2 rises to approximately 97.8%, indicating that its exclusion does not reduce heterogeneity and may marginally increase it. This reflects Assiri's extreme prevalence (0.3%) contributing to heterogeneity but not exclusively.
- Other Notable Deviations:
    - Ewid (2023): Removal lowers I^2 slightly to ≈96.8%, a modest reduction.
    - Alwakeel (2008): Removal raises I^2 to ≈97.9%, suggesting its inclusion slightly tempers overall heterogeneity.
    - AlSidr (2019): Removal raises I^2 to ≈97.8%.
- Stable Cluster: All remaining studies produce I^2 values within approximately 97.2–97.6%, tightly clustered around the reference line, indicating that no other individual study materially shifts the heterogeneity estimate.

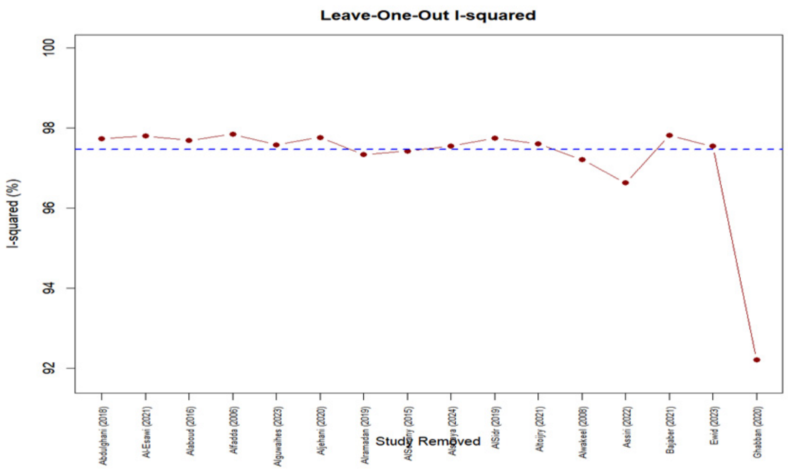

Figure S5 H: Leave-One-Out Sensitivity Analysis of Heterogeneity (I-squared) for the Pooled Prevalence of Myocardial infarction and coronary artery disease among Patients with Type 2 Diabetes Mellitus

Meta-regression analysis of myocardial infarction and coronary artery disease

The meta-regression scatter plots illustrate the relationship between Myocardial infarction and coronary artery disease prevalence (%) and continuous study-level covariates, with bubble sizes indicating study sample size weighting:

- **Positive Associations:**

- **Hypertension (HTN %), Dyslipidemia (%), and Obesity (%):** Each exhibits a clear upward linear regression slope, indicating that study populations with higher proportions of patients with hypertension, dyslipidemia, or obesity reported higher prevalence rates.
- **Publication Year:** Displays a slight upward trend over time.

- **Inverse / Negative Trends:**
    - **DM Duration (years):** Shows a steep downward regression slope, indicating lower reported prevalence in studies with longer mean diabetes duration.
    - **Mean Age (years) & HbA1c (%):** Both demonstrate downward slopes relative to prevalence.
- **Neutral / Minimal Trends:**
    - **Smoking (%) & JBI Score (%):** Exhibit relatively flat regression lines, indicating little to no linear association with prevalence across the included cohorts

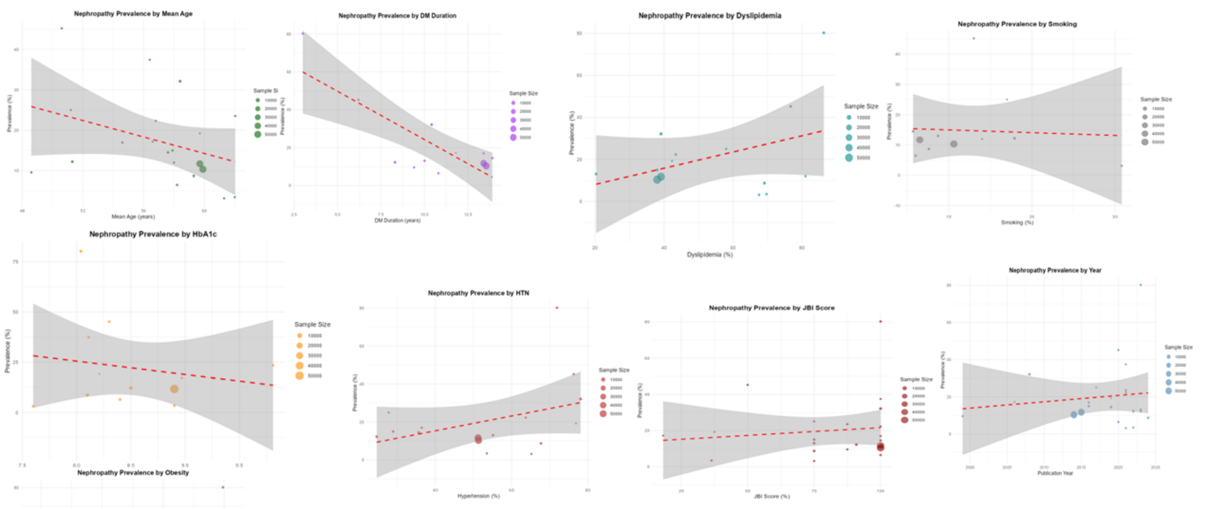

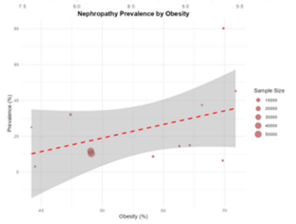

Supplementary figure 5 I: Meta-Regression Analysis of Myocardial infarction and coronary artery disease by Study-Level Moderators (Publication Year, Smoking, Obesity, JBI Score, Hypertension, HbA1cDM Duration, and Mean Age) in Type 2 Diabetes Mellitus

Figure 5I S: Meta-Regression Analysis of Myocardial infarction and coronary artery disease by Study-Level Moderators (publication year, smoking, obesity, JBI Score, hypertension, HbA1c, DM duration, and mean age) in type 2 diabetes mellitus

The univariate meta-regression identified region as the only study-level moderator significantly associated with coronary artery disease and myocardial infarction outcomes. Compared with the reference region, studies from Tabuk (Northern) showed a significantly lower effect estimate (β = −3.3251, 95% CI: −4.7086 to −1.9417, p = 0.0004), while studies from Taif (Western) also demonstrated a significant negative association (β = −4.5865, 95% CI: −7.0564 to −2.1167, p = 0.0023). The associations for Jeddah, Makkah, multi-region studies, and Riyadh were not statistically significant, although Riyadh showed a borderline association (p = 0.0750). Region explained 84.5% of the between-study heterogeneity, with residual I^2 of 87.1%. Study design, JBI score, risk of bias, and diagnostic modality were not significantly associated with the outcome, with R^2 values ranging from 0% to 6.7%. Substantial residual heterogeneity remained in these models, with residual I^2 ranging from 87.1% to 98.0%, indicating that considerable between-study variability was still unexplained. **Supplementary table S5**

Table S5: Univariate meta-regression of potential predictors of coronary artery diseases and myocardial infarctions

Moderator	Comparison	N	β	SE	95% CI	P value	R^2 (%)	I^2 residual (%)
**Region**	Jeddah (Western)	16	0.1092	0.5088	−1.0418 to 1.2602	0.8348	84.5	87.1
	Makkah (Western)	16	−0.8501	0.5254	−2.0387 to 0.3384	0.1401	84.5	87.1

	Multi-region	16	−0.4593	0.4338	−1.4405 to 0.5220	0.3173	84.5	87.1
	Riyadh (Central)	16	−0.7727	0.3839	−1.6411 to 0.0958	0.0750	84.5	87.1
	Tabuk (Northern)	16	−3.3251	0.6116	−4.7086 to −1.9417	0.0004	84.5	87.1
	Taif (Western)	16	−4.5865	1.0918	−7.0564 to −2.1167	0.0023	84.5	87.1
**Study design**	Retrospective cohort	16	−0.2914	0.6486	−1.6824 to 1.0996	0.6601	0.0	97.3
**JBI score (%)**	Continuous	16	0.0016	0.0107	−0.0213 to 0.0246	0.8805	0.0	97.6
**Risk of bias**	Low	16	0.5020	0.7792	−1.1814 to 2.1854	0.5306	0.0	98.0
**Risk of bias**	Moderate	16	0.9222	0.9272	−1.0809 to 2.9253	0.3381	0.0	98.0
**Diagnostic modality**	Confirmed MI	16	−0.8708	0.7248	−2.4253 to 0.6837	0.2495	6.7	97.0

CI = confidence interval. SE = standard error. R^2 = proportion of between-study heterogeneity explained. τ^2 = residual between-study variance. I^2 = residual heterogeneity. JBI = Joanna Briggs Institute.

Peripheral arterial diseases

Publication bias of Peripheral arterial diseases prevalence [Figure S3 X]

The funnel plot displays substantial asymmetry around the overall pooled effect estimate (vertical dashed line at approximately -2.88), pointing to significant publication bias or small-study effects across the included cohorts:

- **Asymmetry and Outlier Distribution:** Most of the included studies lie outside the pseudo-95% confidence bounds (white triangular area). High-precision studies (smaller standard errors near the top) show notable dispersion, with studies positioned to the far left (lower observed prevalence down to -5.3) and to the right (higher observed prevalence extending to -0.75).
- **Small-Study Effects:** Studies with lower precision/larger standard errors (bottom half of the plot) are widely scattered outside the central expected boundary rather than forming a symmetrical, inverted funnel shape.
- **Interpretation:** This marked visual imbalance reflects potential publication bias or substantial clinical and methodological variation across studies, such as differences in PAD diagnostic methods (e.g., Ankle-Brachial Index thresholds vs. symptomatic vascular presentation), clinical settings, or patient age profiles.

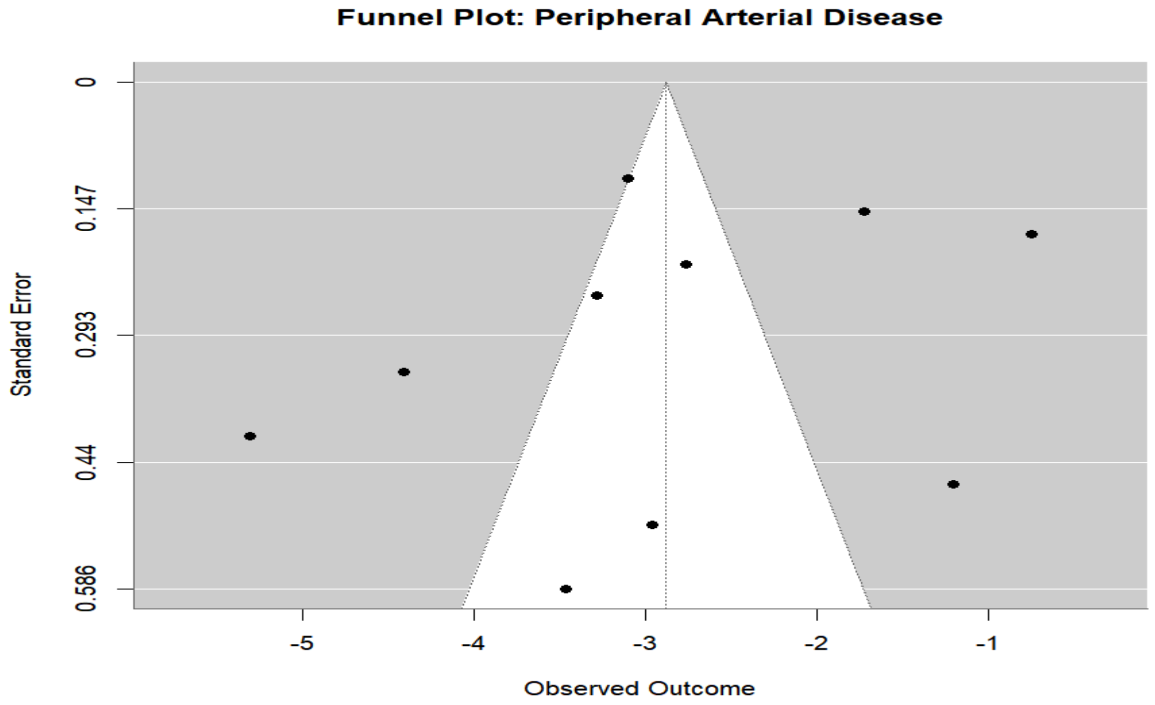

Figure 6X S: Funnel plot included studies assessing the pooled prevalence of Peripheral arterial disease among patients with type 2 diabetes mellitus

Subgroup analysis of peripheral arterial diseases prevalence **[Figure S6 S]**

Subgroup analyses were conducted to evaluate the prevalence of peripheral arterial disease (PAD) complications based on risk of bias, study design, and geographic region:

- **Risk of Bias (Supplementary Figure S6 A):** Pooled prevalence was highest in studies with moderate risk of bias at 12% (95% CI: 2%–44%), followed by high risk at 11% (95% CI: 4%–25%), and low risk at 2% (95% CI: 1%–5%). These subgroup differences were statistically significant ($\chi^2 = 6.84$, df = 2, P = 0.0327).

- **Study Design (Supplementary Figure S6 B):** Cross-sectional studies reported a pooled prevalence of 6% (95% CI: 2%–14%), while cohort studies reported 4% (95% CI: 3%–5%). The test for subgroup differences between study designs was not statistically significant ($\chi^2 = 0.29$, df = 1, P = 0.5893).

- **Geographic Region (Supplementary Figure S6 C):** Pooled prevalence was 15% (95% CI: 12%–19%) in the Western region (based on a single study) compared to

4% (95% CI: 2%–10%) in the Central region. The subgroup difference between geographic regions was statistically significant ($\chi^2 = 7.94$, df = 1, P = 0.0048).

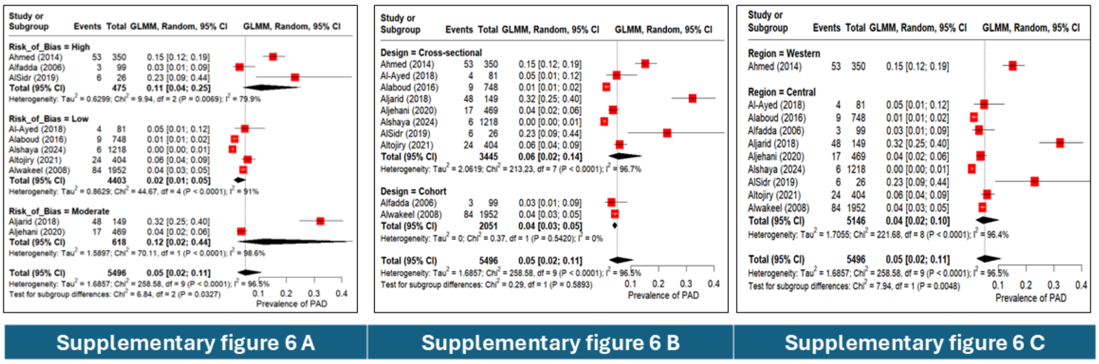

Figure S6 A-C. Subgroup analyses peripheral arterial disease complications prevalence by (A) risk of bias, (B) study design, and (C) geographic region.

Sensitivity analysis of peripheral arterial diseases prevalence

The Baujat plot identifies Study 7 (Alshaya, 2024) and Study 5 (Aljarid, 2018) as the dominant outliers contributing most significantly to overall statistical heterogeneity and overall influence on the pooled outcome:

- **Primary Drivers of Heterogeneity and Influence:**

  Study 7 (Alshaya, 2024) exhibits the highest contribution to statistical heterogeneity (squared Pearson residual ≈ 2.95) and the greatest overall influence score (≈ 0.46). Study 5 (Aljarid, 2018) demonstrates a strong secondary contribution to heterogeneity (squared Pearson residual ≈ 2.45) with a substantial influence score (≈ 0.36).

- **Secondary Influential Studies:**

  Study 8 (AlSidr, 2019) and Study 3 (Alaboud, 2016) show moderate contributions to heterogeneity (squared Pearson residuals between 1.2 and 1.4) with moderate influence scores (≈ 0.14–0.15). Study 1 (Ahmed, 2014) demonstrates a minor contribution to heterogeneity (squared Pearson residual ≈ 0.72) and influence (≈ 0.08).

- **Origin Cluster:**

The remaining studies — including Study 2 (Al-Ayed, 2018), Study 4 (Alfadda, 2006), Study 6 (Aljehani, 2020), and Study 9 (Altoijry, 2021) — cluster near the origin (lower-left quadrant), indicating negligible individual impact on overall heterogeneity and model estimation.

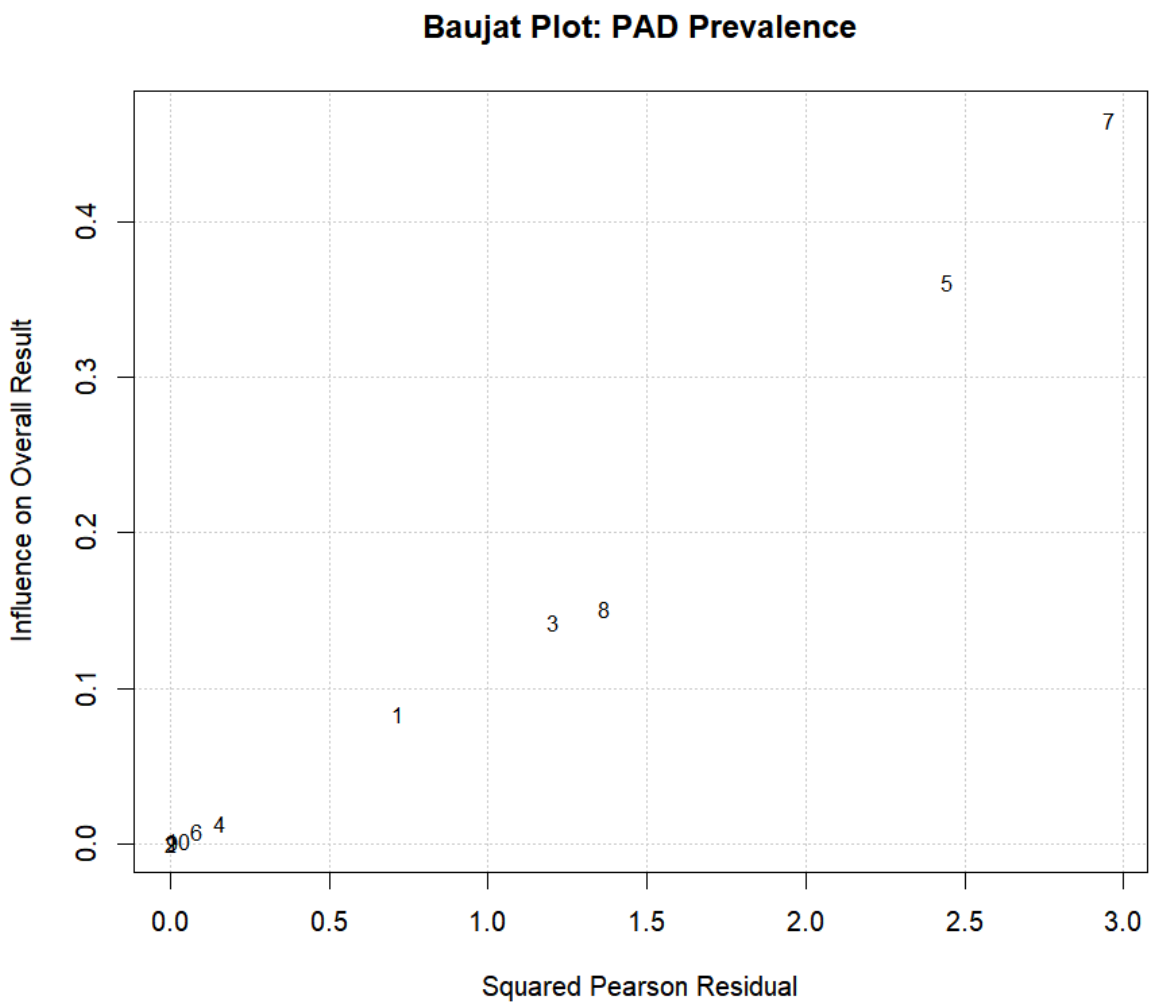

Figure S6 E: Baujat plot of included studies assessing the pooled prevalence of Peripheral arterial disease among patients with type 2 diabetes mellitus

The diagnostic influence plots confirm that **Study 7 (Alshaya, 2024)** and **Study 5 (Aljarid, 2018)** are the most influential studies and primary outliers in the meta-analysis:

Residuals & Influence Metrics (rstudent, dffits, cook.d):

Study 7 (Alshaya, 2024) records the highest Cook's distance (≈ 0.37), alongside a large negative studentized residual (≈ −2.1) and the lowest DFFITS value (≈ −0.7), confirming its strong leverage over the pooled estimate. Study 5 (Aljarid, 2018) exhibits a peak in Cook's distance (≈ 0.32), with a large positive studentized residual (≈ 1.9) and the highest DFFITS value (≈ 0.65). Study 3 (Alaboud, 2016) and Study 8 (AlSidr, 2019) show moderate secondary influence in Cook's distance (≈ 0.15).

Heterogeneity & Variance Impact (tau2.del, QE.del, cov.r):

Omitting Study 7 leads to the sharpest drop in total residual variance (τ^2 decreases from > 1.8 down to ≈ 1.30) and records the lowest covariance ratio (cov.r ≈ 0.79). Omitting Study 5 produces the single largest reduction in Cochrane's Q test statistic (QE.del drops from ≈ 260 down to ≈ 138) and reduces τ^2 to ≈ 1.38.

Leverage & Weight Distribution (hat, weight):

Leverage values (hat ≈ 0.10) and study weights (weight ≈ 10.0) remain balanced and uniform across all 10 included studies. This confirms that high influence is driven by variance in reported prevalence rather than disproportionate weighting. **Supplementary Figure S6 F.**

Supplementary figure S6 E

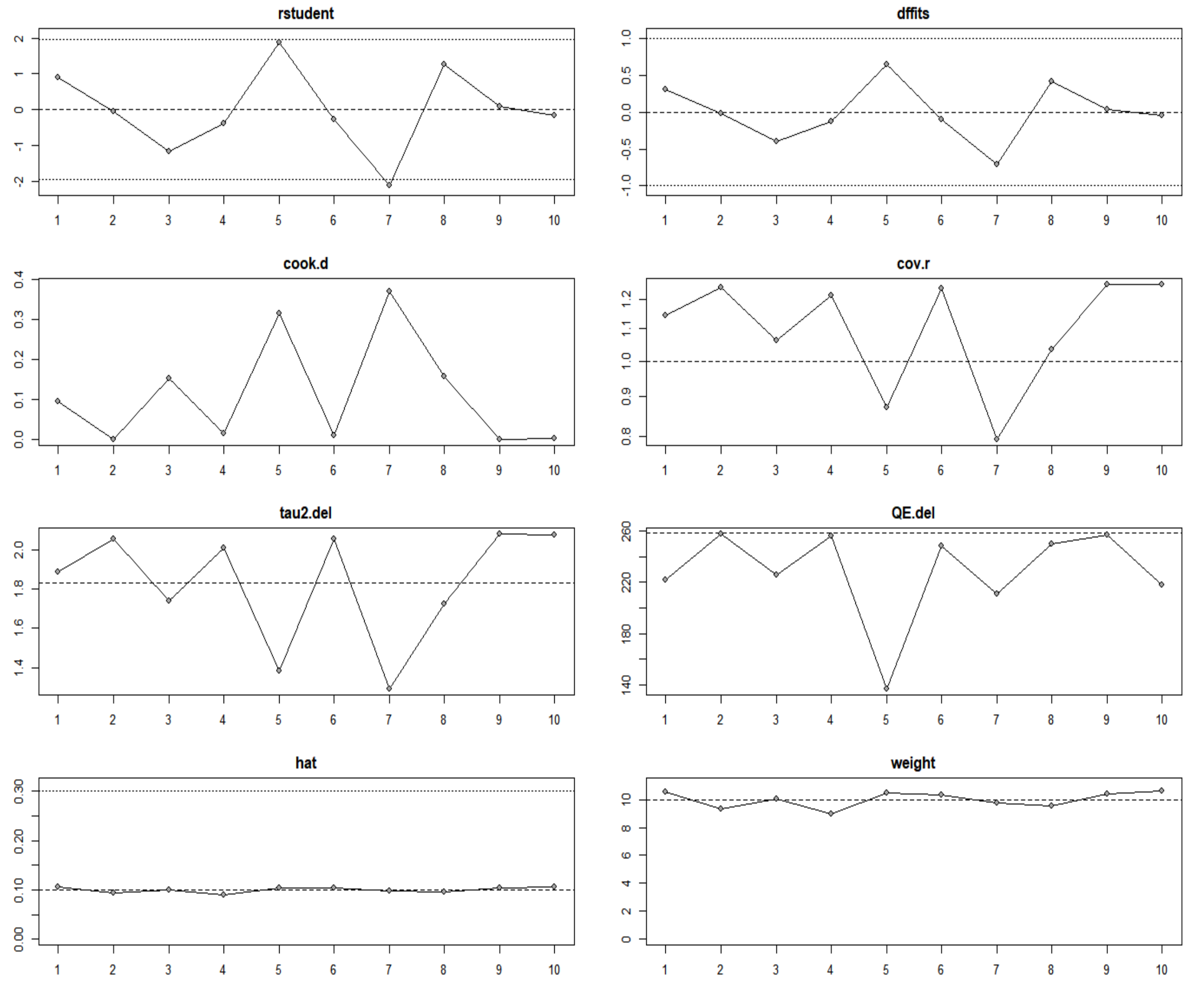

Figure S6 F: Influence analysis and diagnostic plots for meta-analysis of peripheral arterial disease prevalence in type 2 diabetes mellitus

Here is the leave-one-out sensitivity analysis assessment of effect sizes for the meta-analysis of peripheral arterial disease (PAD) prevalence among patients with type 2 diabetes mellitus:

The leave-one-out sensitivity plot demonstrates the sensitivity and overall stability of the pooled PAD prevalence estimate upon sequentially removing individual studies from the baseline model (dashed red line at approximately -2.88 logit prevalence):

- Primary Influential Shifts:

- Omitting Alshaya (2024) causes the most pronounced upward shift in recalculated logit prevalence (to approximately -2.60), confirming that this study reported a significantly lower PAD prevalence that pulled the overall pooled estimate down.
    - Omitting Aljarid (2018) produces the largest downward shift (to approximately -3.12), demonstrating that its higher reported prevalence rate pulled the overall pooled estimate up.
    - Minor downward shifts are also observed when sequentially excluding Ahmed (2014) and AlSidr (2019) (to around -3.01 and -3.05, respectively).
    - Omitting Alaboud (2016) results in a minor upward shift (to around -2.70).
- Overall Robustness: Sequentially omitting neutral studies—such as Al-Ayed (2018), Alfadda (2006), Aljehani (2020), Altoijry (2021), and Alwakeel (2008)—leaves the recalculated point estimate virtually unchanged from the baseline. Wide, broadly overlapping 95% confidence intervals across all iterations confirm that the primary pooled estimate remains generally robust. Supplementary figure 6 G

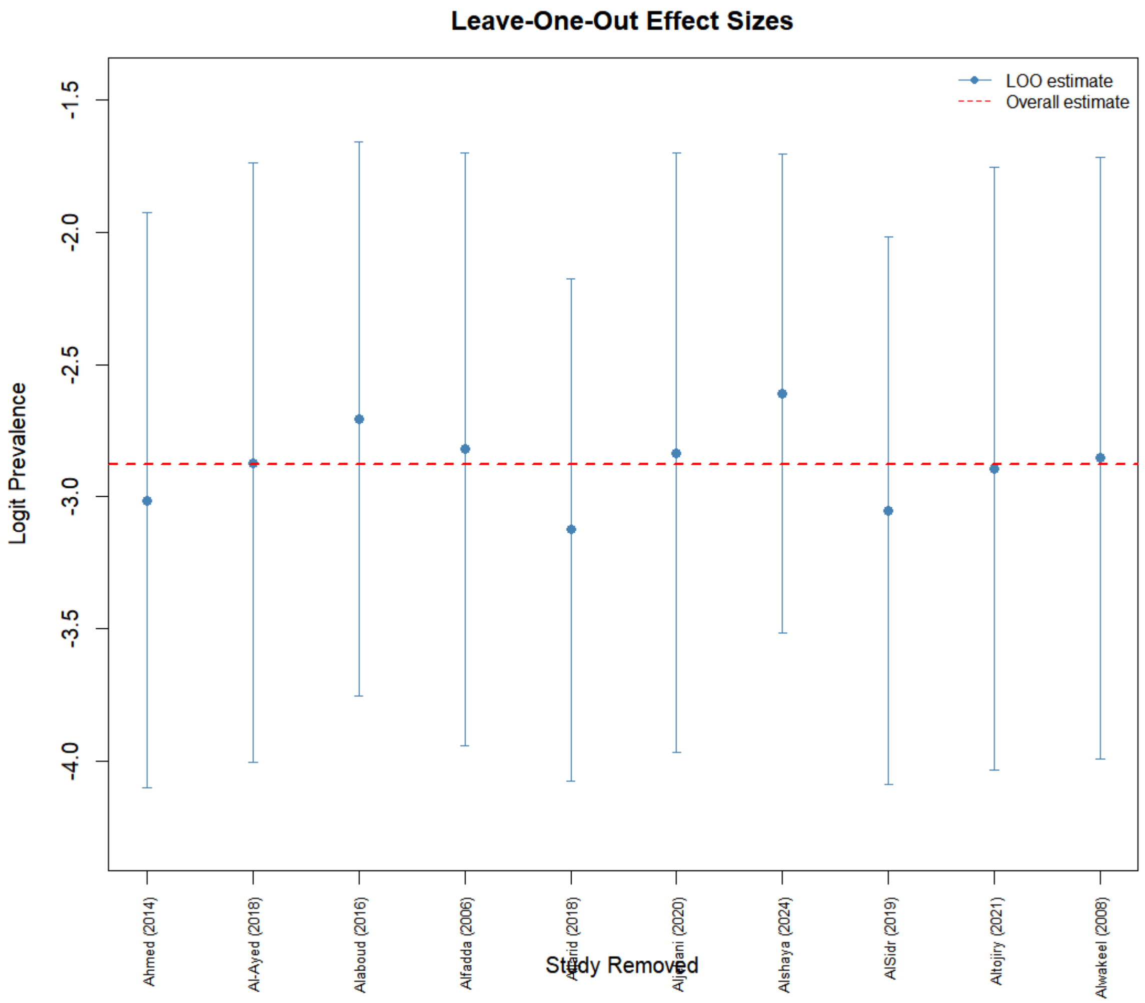

Figure S6 G: Leave-one-out sensitivity analysis of the pooled logit prevalence of Peripheral arterial disease

The leave-one-out I^2 plot evaluates changes in overall statistical heterogeneity upon sequentially excluding individual studies relative to the overall baseline level (dashed blue line at $I^2 = 97.28\%$):

Primary Contributors to Heterogeneity:

Omitting Aljarid (2018) causes the sharpest drop in statistical heterogeneity, reducing I^2 from the baseline of 97.28% down to approximately 96.05%. Omitting Alshaya (2024) produces the second largest reduction in heterogeneity, decreasing I^2 to around 96.45%.

Secondary dips are also observed when sequentially excluding Ahmed (2014) ($I^2 \approx$ 96.85%) and Alwakeel (2008) ($I^2 \approx 96.75\%$).

Minor Increases in Heterogeneity:

Sequentially omitting Al-Ayed (2018) or Alfadda (2006) leads to slight upward shifts in I^2 (increasing to around 97.75%), indicating that these studies report prevalence estimates closer to the pooled mean and help stabilize model variance.

Systemic Heterogeneity:

Despite the observed reductions upon removing key outlier studies, I^2 remains consistently high (> 96.0%) across all leave-one-out iterations. This indicates that substantial statistical heterogeneity is an inherent feature of the overall PAD body of literature — likely reflecting clinical differences in diagnostic methods (e.g., Ankle-Brachial Index screening vs. symptomatic presentation), patient age profiles, and disease duration — rather than an artifact driven by any single study. **Supplementary Figure S6 H.**

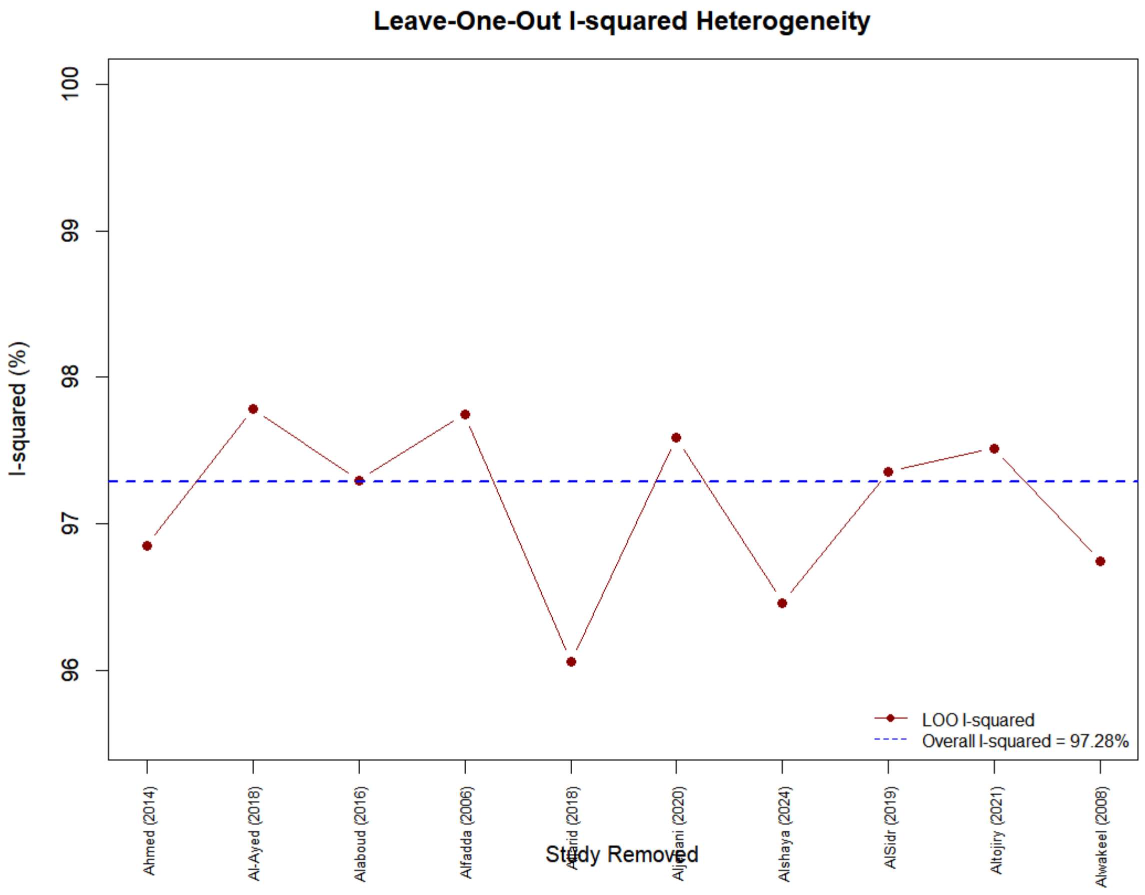

Figure S6 H: Leave-One-Out Sensitivity Analysis of Heterogeneity (I-squared) for the Pooled Prevalence of Peripheral arterial disease among Patients with Type 2 Diabetes Mellitus

Meta-regression analysis of peripheral arterial disease prevalence

Here is the meta-regression analysis assessment across study and clinical moderators for the pooled prevalence of peripheral arterial disease (PAD) among patients with type 2 diabetes mellitus:

The meta-regression scatter plots illustrate the relationships between PAD prevalence (%) and continuous study-level covariates, with bubble sizes reflecting study sample size weighting:

- Positive Associations:

- Smoking (%): Displays a steep, pronounced upward linear regression slope, indicating that study populations with higher smoking rates reported substantially higher prevalence rates of peripheral arterial disease.
- Mean Age (years) & Publication Year: Both demonstrate modest upward linear regression trends, reflecting slightly higher reported PAD prevalence in older patient cohorts as well as in more recent publication years.
- DM Duration (years): Displays a slight positive regression line, suggesting a minor increase in reported PAD prevalence with longer mean diabetes duration.

- Inverse / Negative Trends:
  - JBI Score (%): Exhibits a downward regression slope, indicating that studies with higher methodological quality scores reported lower rates of PAD.
  - Obesity (%): Displays a negative slope, showing lower reported PAD prevalence in patient cohorts with higher obesity rates.
  - HbA1c (%): Demonstrates a subtle downward trend across the evaluated studies.
- Neutral / Minimal Trends:
  - Hypertension (HTN %) & Dyslipidemia (%): Both exhibit essentially flat regression lines with wide confidence bands, indicating little to no linear association with peripheral arterial disease prevalence across the included studies. **Supplementary figure S6 I**

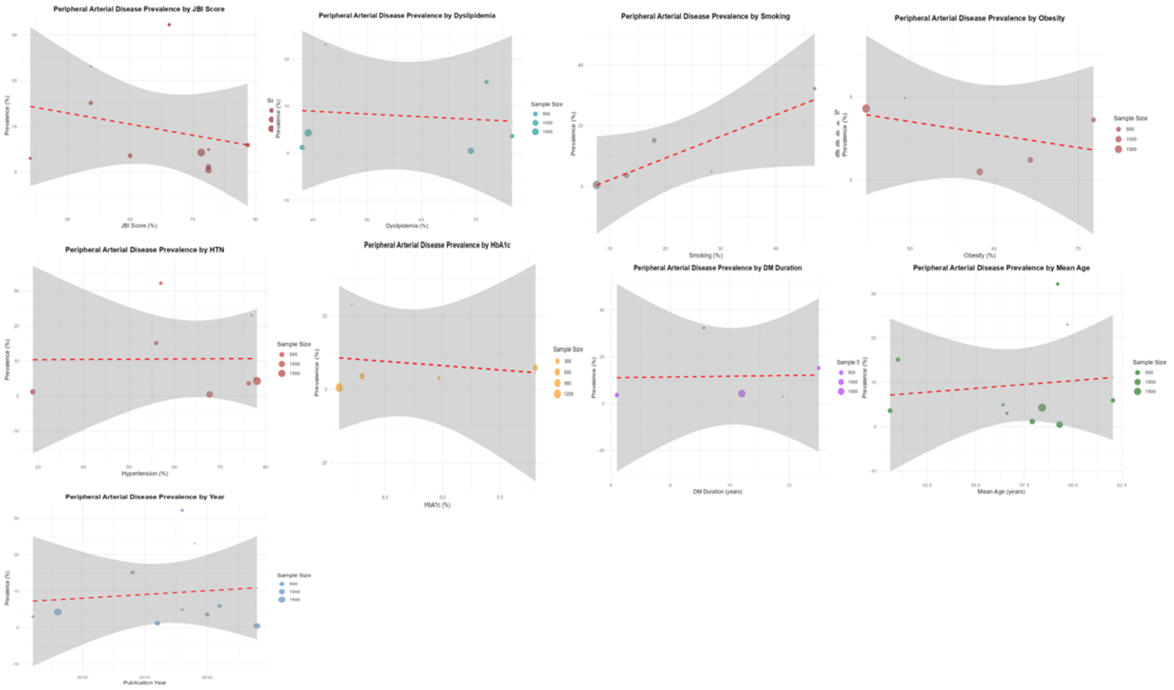

Figure S6 I: Meta-Regression Analysis of Peripheral arterial disease by Study-Level Moderators (Publication year, smoking, obesity, JBI Score, hypertension, HbA1c, DM duration, and mean Age) in Type 2 Diabetes Mellitus

The meta-regression showed that female proportion was the only statistically significant moderator of the pooled effect estimate. A higher proportion of female participants was associated with a lower effect estimate ($\beta = -0.0842$, 95% CI −0.1471 to −0.0213, $P = 0.015$), and this variable explained 53.2% of the between-study variance. However, substantial residual heterogeneity remained ($I^2 = 93.9\%$), indicating that female proportion did not fully explain the variability between studies.

The other examined moderators, including mean age, smoking, dyslipidemia, hypertension, diabetes duration, HbA1c, obesity, JBI score, region, risk of bias, study design, diagnostic modality, and publication year, were not statistically significant.

The parsimonious model retained publication year, but publication year was not statistically associated with the effect estimate ($\beta = -0.0219$, $P = 0.8093$) and explained 0% of the variance. Residual heterogeneity remained very high ($I^2 = 97.1\%$, $\tau^2 = 2.0504$), with a significant heterogeneity test ($QE = 248.8$, $P < 0.0001$). **Supplementary Table 6**

Table S6: Univariate meta-regression of potential predictors of peripheral arterial disease

Moderator	Studies	β coefficient	SE	95% CI	P value	R^2 (%)	Residual I^2 (%)
**Female (%)**	10	-0.0842	0.0273	-0.1471 to -0.0213	**0.0150**	53.2	93.9
**Smoking (%)**	5	0.0884	0.0377	-0.0316 to 0.2084	0.1009	53.9	94.8
**Dyslipidemia (%)**	6	-0.0065	0.0421	-0.1235 to 0.1105	0.8848	0.0	97.6
**Hypertension (%)**	8	0.0151	0.0367	-0.0747 to 0.1049	0.6947	0.0	98.0
**Diabetes mellitus duration**	5	0.0736	0.2558	-0.7403 to 0.8875	0.7923	0.0	98.1
**HbA1c**	5	0.3673	1.1459	-3.2796 to 4.0142	0.7696	0.0	94.3
**Obesity (%)**	5	-0.0268	0.0495	-0.1844 to 0.1307	0.6254	0.0	90.0
**Mean age**	10	-0.0134	0.1315	-0.3166 to 0.2898	0.9211	0.0	97.3
**JBI score (%)**	10	-0.0169	0.0185	-0.0596 to 0.0259	0.3894	0.0	96.6
**Region**	10	-0.9795	1.6071	-4.7798 to 2.8208	0.5615	32.5	94.4
**Risk of bias**	10	-1.6112	0.9156	-3.7763 to 0.5539	0.1219	23.4	95.2
**Study design**	10	0.4057	1.0196	-1.9454 to 2.7568	0.7011	0.0	96.8
**Diagnostic modality**	10	-1.4258	1.0328	-3.8075 to 0.9558	0.2048	10.0	96.7
**Publication year**	10	-0.0219	0.0878	-0.2243 to 0.1805	0.8093	0.0	97.1

Diabetic foot ulcer

Publication bias of diabetic foot ulcer prevalence [Figure S3 X]

The funnel plot displays marked visual asymmetry around the central pooled effect estimate (vertical dashed line at approximately -2.18 logit prevalence), indicating potential publication bias, small-study effects, or substantial clinical heterogeneity:

- Asymmetry and Outlier Distribution: A large majority of the included studies fall entirely outside the pseudo-95% confidence bounds (white triangular area). High-precision studies (smaller standard errors near the top) show notable scatter on either side of the average effect, ranging from -3.95 on the left to -0.75 on the right.
- Extreme Outliers and Lower Precision Studies: Studies with larger standard errors (lower portion of the vertical axis) are widely dispersed across both extremes. Notably, one severe extreme outlier is positioned on the far right at an observed logit outcome of approximately +1.65, while several low-precision studies cluster on the far left between -3.2 and -3.7.

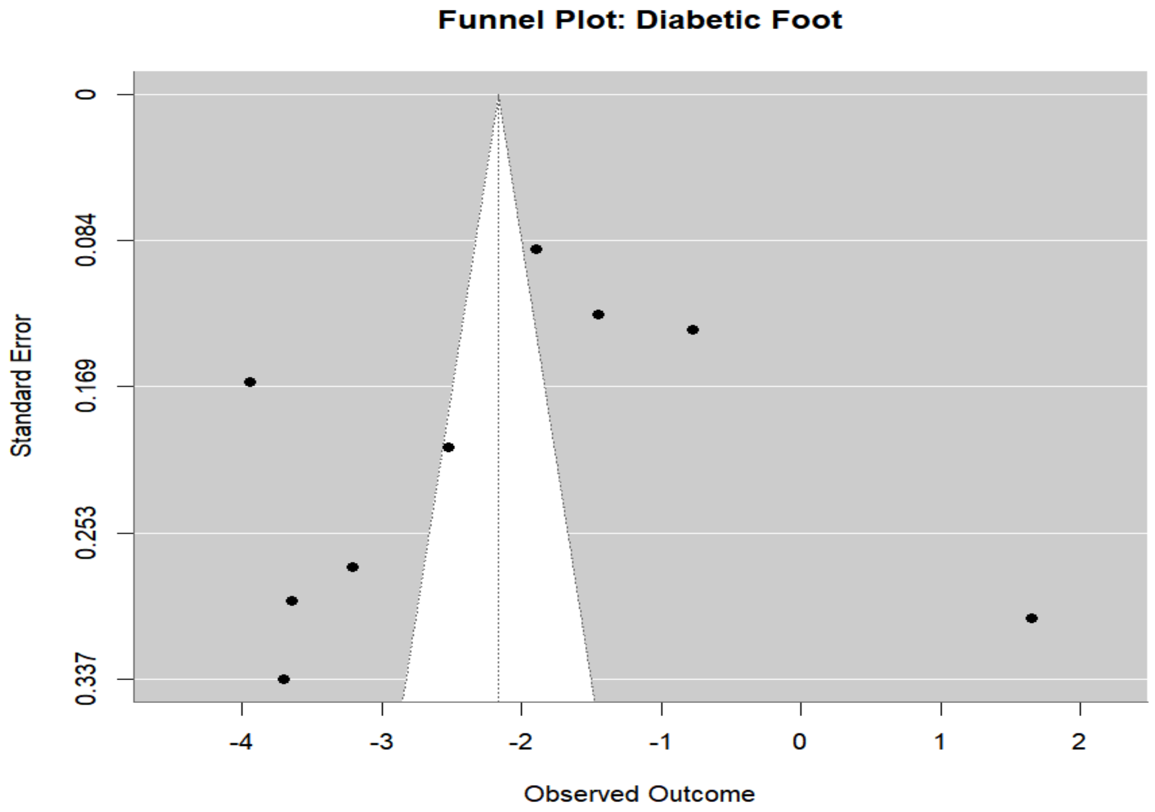

Figure S7 X: Funnel plot included studies assessing the pooled prevalence of diabetic foot among patients with type 2 diabetes mellitus

Subgroup analysis of diabetic foot ulcer prevalence **[Figure S7 S]**

Subgroup analyses were conducted to evaluate the prevalence of diabetic foot in Saudi Arabia based on risk of bias, study design, geographic region, and diagnostic criteria:

Risk of Bias (Supplementary Figure S7 A): The pooled prevalence of diabetic foot was higher in studies with a low risk of bias at 14% (95% CI: 4%–38%) compared to those with a moderate risk of bias at 3% (95% CI: 2%–5%). These subgroup differences were statistically significant ($\chi^2 = 5.35$, df = 1, P = 0.0208).

Study Design (Supplementary Figure S7 B): Cross-sectional studies reported a pooled prevalence of 12% (95% CI: 4%–31%), whereas retrospective cohort studies reported 2% (95% CI: 1%–3%). The test for subgroup differences between study designs was statistically significant ($\chi^2 = 10.44$, df = 1, P = 0.0012).

Geographic Region (Supplementary Figure S7 C): Prevalence varied across regions, ranging from 8% (95% CI: 2%–26%) in Riyadh (Central region) to 31% (95% CI: 26%–38%) in Makkah (Western region), with multi-region studies reporting 13% (95% CI: 11%–15%). Regional subgroup differences were statistically significant (χ^2 = 48.87, df = 2, P < 0.0001).

Diagnostic Criteria (Supplementary Figure S7 D): Studies assessing a broader diabetic-foot phenotype yielded a higher pooled prevalence of 47% (95% CI: 7%–91%) compared to those specifically evaluating active ulcers at 6% (95% CI: 3%–13%). The test for subgroup differences bordered on statistical significance (χ^2 = 3.82, df = 1, P = 0.0505).

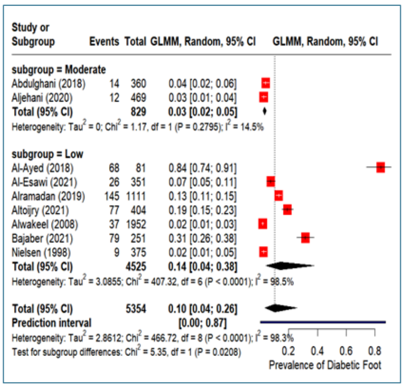

Supplementary figure 7 A

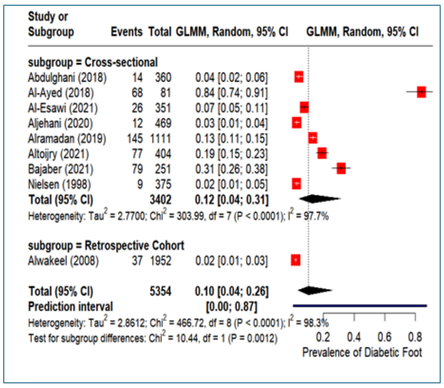

Supplementary figure 7 B

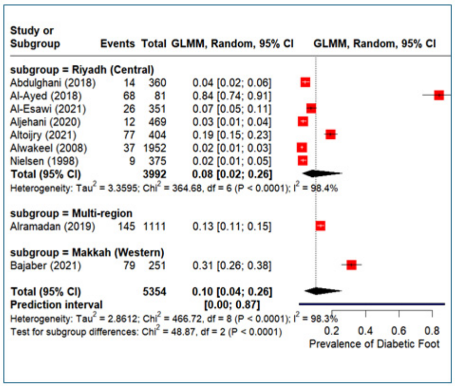

Supplementary figure 7 C

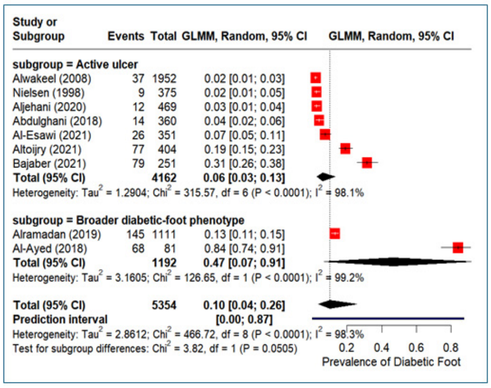

Supplementary figure 7 D

Figure S7 A-C. Subgroup analyses peripheral arterial disease complications prevalence by (A) risk of bias, (B) study design, (C) geographic region, and diagnostic criteria (D)

Sensitivity analysis of diabetic foot prevalence

The Baujat plot identifies Study 2 (Al-Ayed, 2018) as the dominant outlier and primary contributor to overall statistical heterogeneity and overall influence on the pooled outcome:

- Primary Driver of Heterogeneity and Influence:
  - Study 2 (Al-Ayed, 2018) exhibits by far the highest contribution to statistical heterogeneity (squared Pearson residual ≈ 4.5) and the greatest overall influence on the pooled result (influence score ≈ 1.2).
- Secondary Influential Studies:
  - Study 7 (Alwakeel, 2008) shows a moderate contribution to heterogeneity (squared Pearson residual ≈ 1.0) with a minor influence score (≈ 0.14).
  - Study 8 (Bajaber, 2021) and Study 9 (Nielsen, 1998) display minor contributions with squared Pearson residuals around 0.7 to 0.8.
- Origin Cluster:
  - The remaining studies—including Study 1 (Abdulghani, 2018), Study 3 (Al-Esawi, 2021), Study 4 (Aljehani, 2020), Study 5 (Alramadan, 2019), and Study 6 (Altoijry, 2021)—cluster tightly near the origin (squared Pearson residuals < 0.5, influence near 0.0), indicating negligible individual impact on overall model heterogeneity and estimation. **Supplementary figure S7 E**

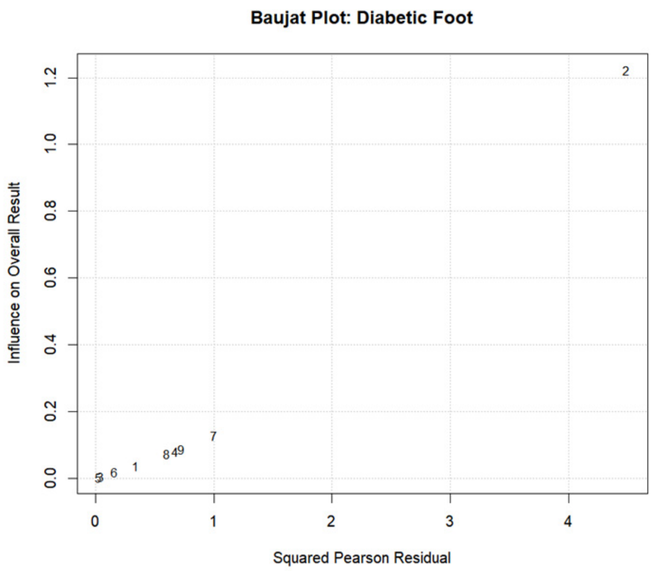

Figure S7 E: Baujat plot included studies assessing the pooled prevalence of diabetic foot among patients with type 2 diabetes mellitus

The diagnostic influence plots confirm that Study 2 (Al-Ayed, 2018) is by far the most influential study and primary statistical outlier in the meta-analysis:

Residuals & Influence Metrics (rstudent, dffits, cook.d):

Study 2 (Al-Ayed, 2018) records an extreme peak in Cook's distance (≈ 0.60), accompanied by a very high positive studentized residual (≈ 3.3) and the highest DFFITS value (≈ 1.15), significantly exceeding standard thresholds. The remaining studies (1 through 9) exhibit low Cook's distances (< 0.20) and studentized residuals well within the standard ±2.0 boundaries.

Heterogeneity & Variance Impact (tau2.del, QE.del, cov.r):

Omitting Study 2 leads to a dramatic drop in total residual variance (τ^2 plummets from > 30 down to ≈ 14) and results in the lowest covariance ratio (cov.r ≈ 0.55). Similarly, omitting Study 2 (and alternatively Study 7 [Alwakeel, 2008]) produces the largest reductions in Cochrane's Q test statistic (QE.del drops from ≈ 450 down to ≈ 320 and ≈ 305, respectively).

Leverage & Weight Distribution (hat, weight):

Leverage values (hat ≈ 0.11) and study weights (weight ≈ 11.0) are entirely uniform and balanced across all 9 included studies. This confirms that the overwhelming influence of Study 2 is driven entirely by its divergent reported effect size rather than disproportionate sample size weighting. **Supplementary Figure S7 F.**

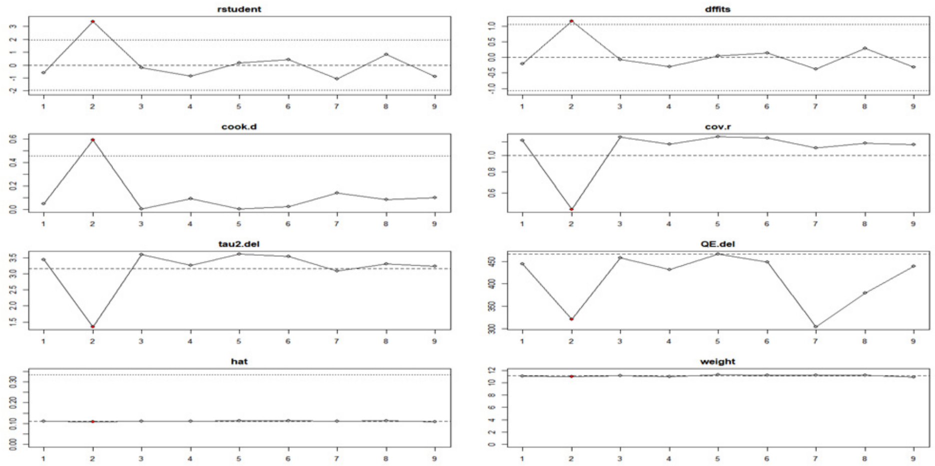

Figure S7 F: Influence analysis and diagnostic plots for meta-analysis of diabetic foot prevalence in type 2 diabetes mellitus

The leave-one-out sensitivity plot demonstrates the stability and robustness of the pooled diabetic foot prevalence estimate when sequentially excluding individual studies from the baseline model (dashed red line at approximately -2.18 logit prevalence):

- Primary Influential Shifts:
  - Omitting Al-Ayed (2018) produces the most pronounced downward shift in the recalculated logit prevalence (to approximately -2.62), confirming that this study reported a significantly higher diabetic foot prevalence that pulled the overall pooled estimate upward.
  - Omitting Bajaber (2021) results in a moderate downward shift in logit prevalence (to approximately -2.35).
- Upward and Neutral Shifts:
  - Omitting Aljehani (2020) and Alwakeel (2008) leads to slight upward shifts in the recalculated logit prevalence (to around -1.95 and -1.92, respectively).

- Sequentially omitting other studies—such as Abdulghani (2018), Al-Esawi (2021), Alramadan (2019), Altoijry (2021), and Nielsen (1998)—leaves the recalculated point estimate closely aligned with the overall baseline.

- Overall Robustness: Broadly overlapping 95% confidence intervals across all iterations confirm that the primary pooled prevalence estimate remains stable and robust despite the influence of individual study outliers. **Supplementary figure S7 G**

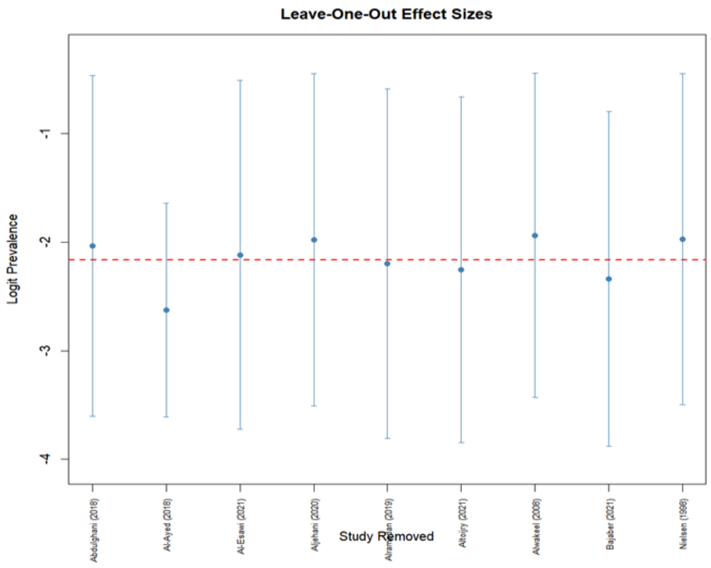

Figure S7 G: Leave-one-out sensitivity analysis of the pooled logit prevalence of Diabetic foot

The leave-one-out I^2 plot evaluates changes in overall statistical heterogeneity upon sequentially excluding individual studies relative to the baseline level (dashed blue line at $I^2 = 99.09\%$):

- **Primary Driver of Heterogeneity:**
  - Omitting **Al-Ayed (2018)** causes the sharpest and most pronounced drop in statistical heterogeneity, reducing I^2 from the baseline down to approximately 98.05%.
- **Minor Fluctuations and Increases:**

- Omitting Alramadan (2019) shows a slight decrease to around 98.94%.
- Sequentially omitting Abdulghani (2018), Al-Esawi (2021), Aljehani (2020), and Nielsen (1998) leads to very minor upward shifts in I^2 (ranging up to approximately 99.23%), indicating that these studies report prevalence variations that slightly stretch model variance.
- Omitting Altoijry (2021), Alwakeel (2008), and Bajaber (2021) leaves I^2 virtually identical to the baseline.

- **Extreme Systemic Heterogeneity:** Across all leave-one-out iterations, I^2 remains exceptionally high (> 98.0%). This confirms that massive statistical heterogeneity is a systemic property of the diabetic foot literature—driven by clinical, methodological, and geographic variations in ulcer definition and screening—rather than being an artifact of any single study. **Supplementary figure S6 H**

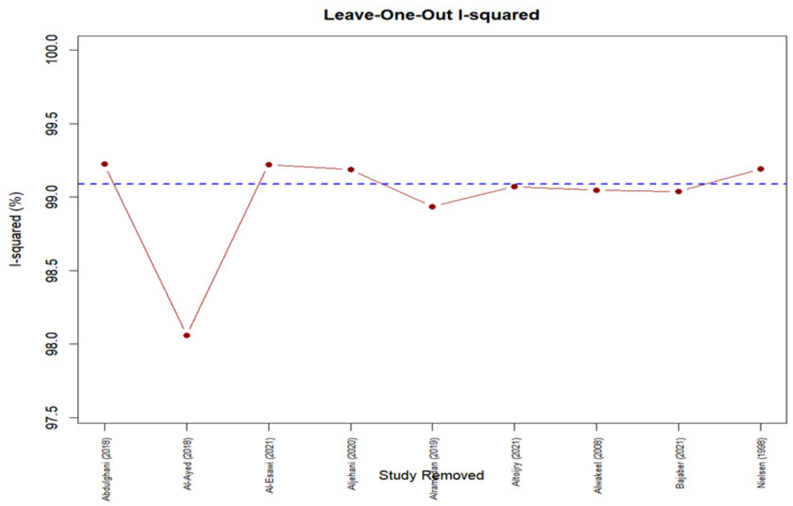

Figure S6 H: Leave-one-out sensitivity analysis of heterogeneity (I-squared) for the pooled prevalence of diabetic foot among patients with type 2 diabetes mellitus.

Meta-regression analysis of diabetic foot prevalence

The meta-regression scatter plots illustrate the relationships between diabetic foot prevalence (%) and continuous study-level covariates, with bubble sizes reflecting study sample size weighting:

- Positive Associations / Trends:
    - HbA1c (%) & Smoking (%): Both display upward linear regression slopes, indicating that study populations with higher glycemic levels and higher smoking rates report increased prevalence rates of diabetic foot complications.
    - DM Duration (years) & Hypertension (HTN %): Show positive regression trends, suggesting higher reported diabetic foot prevalence in cohorts with longer diabetes duration and greater comorbid hypertension.
    - Mean Age (years) & Publication Year: Demonstrate slight positive upward slopes, reflecting higher reported prevalence in older cohorts and more recently published studies.
- Inverse / Negative Trends:
    - Obesity (%) & Dyslipidemia (%): Exhibit negative downward regression slopes, indicating lower reported diabetic foot prevalence in patient cohorts with higher rates of obesity and dyslipidemia.
- Neutral Trends:
    - JBI Score (%): Displays a completely flat regression line, indicating that methodological quality scores do not systematically bias or alter the reported prevalence of diabetic foot across the included studies. **Supplementary figure S7 I**

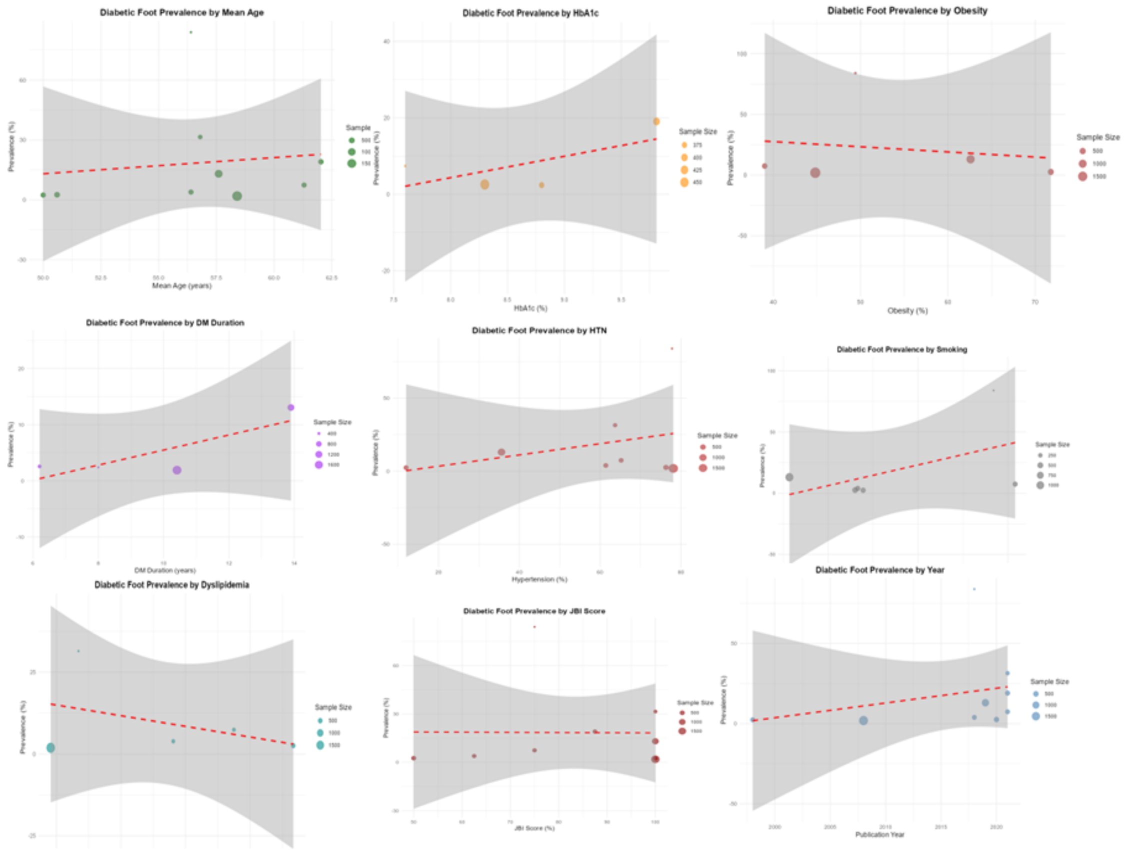

Figure S7 I: Meta-Regression analysis of diabetic foot by study-level moderators (publication year, smoking, obesity, JBI Score, hypertension, HbA1c, DM duration, and mean age) in type 2 diabetes mellitus

Meta-regression was performed to investigate whether study-level characteristics explained heterogeneity in the effect estimates for diabetic foot. None of the evaluated moderators demonstrated a statistically significant association with the pooled effect estimate. Diagnostic modality showed the strongest potential association, with studies using a broader diabetic-foot phenotype demonstrating a higher effect estimate (β = 2.5832, 95% CI −0.2239 to 5.3904; P = 0.0660). This moderator explained 31.7% of the between-study variance; however, the confidence interval included zero and the association did not reach statistical significance.

No significant associations were observed for publication year, mean age, female proportion, diabetes duration, HbA1c, hypertension, obesity, smoking, dyslipidemia, geographical region, study design, JBI score, or risk of bias. The residual heterogeneity remained very high across the analyses, with residual I2 ranging from 91.9% to 99.4%. These findings suggest that the study-level characteristics examined did not adequately

explain the substantial heterogeneity observed in the diabetic-foot outcome. The possible contribution of diagnostic modality warrants consideration, although the current evidence does not establish a statistically significant association. **Supplementary table S7**

Table S7: Univariate meta-regression of potential predictors of diabetic foot

Moderator	Comparison	N	β	SE	95% CI	P value	R^2 (%)	τ^2 residual	I^2 residual (%)
**Year**	Year	9	0.1036	0.0774	−0.0793 to 0.2866	0.2223	9.6	2.8502	99.0
**Mean Age**	Mean Age	9	0.1318	0.1579	−0.2415 to 0.5051	0.4313	0.0	3.2843	99.1
**Female %**	Female %	9	−0.0049	0.0300	−0.0758 to 0.0661	0.8758	0.0	3.6054	99.2
**DM Duration**	DM Duration	4	0.2260	0.1274	−0.3220 to 0.7741	0.2180	41.7	0.5265	91.9
**HbA1c**	HbA1c	4	0.5621	0.7022	−2.4591 to 3.5834	0.5074	0.0	1.2590	94.2
**Hypertension %**	Hypertension %	8	0.0205	0.0322	−0.0583 to 0.0993	0.5473	0.0	3.8789	99.0
**Obesity %**	Obesity %	5	−0.0266	0.0950	−0.3291 to 0.2758	0.7976	0.0	6.4233	99.4
**Smoking %**	Smoking %	6	0.1020	0.0882	−0.1430 to 0.3469	0.3122	5.8	3.7369	98.3
**Dyslipidemia %**	Dyslipidemia %	5	−0.0209	0.0445	−0.1626 to 0.1208	0.6708	0.0	1.9650	98.1
**Region**	Multi-region	9	−1.1184	2.7827	−7.9274 to 5.6907	0.7017	0.0	3.8403	98.8
**Region**	Riyadh (Central)	9	−1.6252	2.1065	−6.7797 to 3.5292	0.4697	0.0	3.8403	98.8
**Study Design**	Retrospective Cohort	9	−2.0076	1.8836	−6.4618 to 2.4465	0.3219	1.9	3.0928	99.0

**JBI Score (%)**	JBI Score	9	0.0063	0.0362	−0.0794 to 0.0919	0.8675	0.0	3.6005	99.2
**Risk of Bias**	Moderate	9	−1.6152	1.4195	−4.9719 to 1.7414	0.2926	3.7	3.0360	99.1
**Diagnostic Modality**	Broader diabetic-foot phenotype	9	2.5832	1.1871	−0.2239 to 5.3904	0.0660	31.7	2.1536	98.3

List of abbreviations:

ABI Ankle-Brachial Index

CAD Coronary Artery Disease

CHD Coronary Heart Disease

CI Confidence Interval

DF Diabetic Foot

DFFITS Difference in Fits (influence diagnostic)

DM Diabetes Mellitus

DN Diabetic Nephropathy

DN4 Douleur Neuropathique-4 (neuropathy questionnaire)

DPN Diabetic Peripheral Neuropathy

DR Diabetic Retinopathy

EMG/NCV Electromyography / Nerve Conduction Velocity

eGFR Estimated Glomerular Filtration Rate

GLMM Generalized Linear Mixed Model

HbA1c Glycated Hemoglobin

HTN Hypertension

I^2 I-squared (heterogeneity statistic)

JBI Joanna Briggs Institute

LOO Leave-One-Out

MI Myocardial Infarction

MNSI Michigan Neuropathy Screening Instrument

mTCN Modified Toronto Clinical Neuropathy Score

N/A Not Applicable

NDS Neuropathy Disability Score

NPDR Non-Proliferative Diabetic Retinopathy

PAD Peripheral Arterial Disease

PDR Proliferative Diabetic Retinopathy

QE Cochrane's Q Test Statistic

R^2 R-squared (proportion of between-study variance explained)

REML Restricted Maximum Likelihood

SD Standard Deviation

SE Standard Error

TBI Toe-Brachial Index

T^2 Tau-squared (between-study variance)

WBC White Blood Cell

WHO World Health Organization

Reference:

1. Al-Ayed, M.Y.; Ababneh, M.; Robert, A.A.; Salman, A.; Al Saeed, A.; Al Dawish, M.A. Evaluation of Risk Factors Associated with Diabetic Foot Ulcers in Saudi Arabia. *Curr. Diabetes Rev.* **2019**, *15*, 224–232, doi:10.2174/1573399814666180816165848.

2. Alshaya, O.A.; Korayem, G.B.; Alghwainm, M.; Alyami, W.; Alotaibi, A.; Alyami, M.S.; Almohammed, O.A. The Prevalence of Cardiovascular Diseases, Chronic Kidney Disease, and Obesity in Patients with Type 2 Diabetes Mellitus and the Description of Concurrent Treatments: A Two-Center Retrospective Cross-Sectional Study in Saudi Arabia. *Saudi Pharmaceutical Journal* **2024**, *32*, 102054, doi:10.1016/j.jsps.2024.102054.

3. AL-Zamil, W. Hospital Prevalence of Retinopathy in Patients with Newly-Diagnosed Type 2 Diabetes. *Saudi J. Med. Med. Sci.* **2017**, *5*, 26, doi:10.4103/1658-631X.194248.

4. Bajaber, B.; Alshareef, M. Correlates of Diabetic Retinopathy in Type 2 Diabetes Mellitus Patients in Makkah Al-Mukarramah, Saudi Arabia. *J. Family Community Med.* **2021**, *28*, 8, doi:10.4103/jfcm.JFCM_334_20.

5. Al-Rubeaan, K.; Youssef, A.M.; Subhani, S.N.; Ahmad, N.A.; Al-Sharqawi, A.H.; Al-Mutlaq, H.M.; David, S.K.; AlNaqeb, D. Diabetic Nephropathy and Its Risk Factors in a Society with a Type 2 Diabetes Epidemic: A Saudi National Diabetes Registry-Based Study. *PLoS One* **2014**, *9*, e88956, doi:10.1371/journal.pone.0088956.

6. Al-Rubeaan, K.; Abu El-Asrar, A.M.; Youssef, A.M.; Subhani, S.N.; Ahmad, N.A.; Al-Sharqawi, A.H.; Alguwaihes, A.; Alotaibi, M.S.; Al-Ghamdi, A.; Ibrahim, H.M. Diabetic Retinopathy and Its Risk Factors in a Society with a Type 2 Diabetes Epidemic: A <scp>S</Scp> Audi <scp>N</Scp> Ational <scp>D</Scp> Iabetes <scp>R</Scp> Egistry-based Study. *Acta Ophthalmol.* **2015**, *93*, doi:10.1111/aos.12532.

7. Alaboud, A.F.; Tourkmani, A.M.; Alharbi, T.J.; Alobikan, A.H.; Abdelhay, O.; Batal, S.M. Al; Alkhashan, H.I.; Mohammed, U.Y. Microvascular and Macrovascular Complications of Type 2 Diabetic Mellitus in Central, Kingdom of Saudi Arabia. *Saudi Med. J.* **2016**, *37*, 1408–1411, doi:10.15537/smj.2016.12.17062.

8. Assiri, A.A.; Alahmari, K.A.; Alqhtani, N.S.; AlShamrani, N.A.; Mubarak, A.S.; Alshehri, K.M.; Alselmi, A.A. Prevalence of Cardiovascular Events among Patients with Type 2 Diabetes in the West Region of Saudi Arabia. *Saudi Pharmaceutical Journal* **2022**, *30*, 1825–1829, doi:10.1016/j.jsps.2022.10.012.

9. Alnaim, M.M.; Alrasheed, A.; Alkhateeb, A.A.; Aljaafari, M.M.; Alismail, A. Prevalence of Microvascular Complications among Patients with Type 2 Diabetes Mellitus Who Visited Diabetes Clinics in Saudi Arabia. *Saudi Med. J.* **2023**, *44*, 211–217, doi:10.15537/smj.2023.44.2.20220719.

10. Al-Esawi, H.; A Amer, S. Prevalence of Complications among Saudi Males Type 2 Diabetic Patients in Riyadh Primary Health Care Centers, 2019. *Diabetes Updates* **2021**, *7*, doi:10.15761/DU.1000158.

11. Abdulghani, H.M.; AlRajeh, A.S.; AlSalman, B.H.; AlTurki, L.S.; AlNajashi, N.S.; Irshad, M.; Alharbi, K.H.; AlBalawi, Y.E.; AlSuliman, Y.A.; Ahmad, T. Prevalence of Diabetic Comorbidities and Knowledge and Practices of Foot Care among Diabetic Patients: A Cross-Sectional Study. *Diabetes Metab. Syndr. Obes.* **2018**, *Volume 11*, 417–425, doi:10.2147/DMSO.S171526.

12. AlSidr, K.S.; Alotaibi, M.A.K. Diabetic Foot and Its Clinical Characteristics among Uncontrolled Diabetic Patients in Ad-Dawadmi General Hospital, Riyadh Province, KSA. *Saudi Journal of Biomedical Research* **2019**, *4*, 248–253.

13. Alramadan, M.J.; Magliano, D.J.; Alhamrani, H.A.; Alramadan, A.J.; Alameer, S.M.; Amin, G.M.; Alkharras, W.A.; Bayaseh, N.A.; Billah, B. Lifestyle Factors and Macro- and Micro-Vascular Complications among People with Type 2 Diabetes in Saudi Arabia. *Diabetes & Metabolic Syndrome: Clinical Research & Reviews* **2019**, *13*, 484–491, doi:10.1016/j.dsx.2018.11.007.

14. Vesti Nielsen, J. Peripheral Neuropathy, Hypertension, Foot Ulcers and Amputations among Saudi Arabian Patients with Type 2 Diabetes. *Diabetes Res. Clin. Pract.* **1998**, *41*, 63–69, doi:10.1016/S0168-8227(98)00059-X.

15. Ewid, M.; Algoblan, A.S.; Elzaki, E.M.; Muqresh, M.A.; Al Khalifa, A.R.; Alshargabi, A.M.; Alotaibi, S.A.; Alfayez, A.S.; Naguib, M. Factors Associated with Glycemic Control and Diabetes Complications in a Group of Saudi Patients with Type 2 Diabetes. *Medicine* **2023**, *102*, e35212, doi:10.1097/MD.0000000000035212.

16. Aljehani, M.; Mohammed, A.; Alhassan, F. Prevalence of Diabetic Complication in Relation to Risk Factors among Patients with Type 2 Diabetes at University Hospital in Riyadh, Saudi Arabia . *J. Family Community Med.* **2020**, *27*, 28–35.

17. Alguwaihes, A.M.; Alhozali, A.; Yahia, M.M.; Abdel-Nabi, T.; Hassan Hatahet, M.; Albalkhi, N.I.; Al Sifri, S. The Prevalence of Cardiovascular Disease in Adults with Type 2 Diabetes Mellitus in Saudi Arabia - CAPTURE Study. *Saudi Med. J.* **2023**, *44*, 57–66, doi:10.15537/smj.2023.44.1.20220402.

18. Ghabban, S.J.; Althobaiti, B.; Farouk, I.; Al Hablany, M.; Ghabban, A.; Alghbban, R.; Harbi, S.; Albalawi, A.E. Diabetic Complications and Factors Affecting Glycemic Control Among Patients With Type II Diabetes Mellitus Attending the Chronic Illness Clinics at Tabuk, Saudi Arabia. *Cureus* **2020**, doi:10.7759/cureus.11683.

19. Alsenany, S.; Al Saif, A. Incidence of Diabetes Mellitus Type 2 Complications among Saudi Adult Patients at Primary Health Care Center. *J. Phys. Ther. Sci.* **2015**, *27*, 1727–1730, doi:10.1589/jpts.27.1727.

20. Alwakeel, J.S.; Sulimani, R.; Al-Asaad, H.; Al-Harbi, A.; Tarif, N.; Al-Suwaida, A.; Al-Mohaya, S.; Isnani, A.C.; Alam, A.; Hammad, D. Diabetes Complications in 1952 Type 2 Diabetes Mellitus Patients Managed in a Single Institution. *Ann. Saudi Med.* **2008**, *28*, 260–266, doi:10.5144/0256-4947.2008.260.

21. Aljarid, J.S.; Alazmi, A.M.; Almaeen, A.H.; Alruwaili, A.O.; Ragheb, M.M.; Almousara, N. mousara Prevalence and Pattern of Peripheral Arterial Disease among Diabetic Individual in Al-Jouf Region. *Egypt. J. Hosp. Med.* **2018**, *73*, 6641–6645, doi:10.21608/ejhm.2018.28675.

22. Ahmed, A.A.; Algamdi, S.A. Risk Factors for Diabetic Foot Ulceration among Patients Attending Primary Health Care Services . *The Journal of Diabetic Foot Complications* **2014**, *6*, 40–47.

23. Alfadda, A.; Abdulrahman, K.A. Bin Assessment of Care for Type 2 Diabetic Patients at the Primary Care Clinics of a Referral Hospital. *J. Family Community Med.* **2006**, *13*, 13–18.

24. Altoijry, A.; AlGhofili, H.; Alanazi, S.N.; AlHindawi, D.A.; AlAkeel, N.S.; Julaidan, B.S.; AlHamzah, M.; Altuwaijri, T. Diabetic Foot and Peripheral Arterial Disease. *Saudi Med. J.* **2021**, *42*, 49–55, doi:10.15537/smj.2021.1.25640.
